# Supplementary material for: Temporal shifts in HIV-related risk factors among cohorts of adolescent girls and young women enrolled in DREAMS programming: evidence from Kenya, Malawi and Zambia
Source: BMJ Open. 2022 Feb 1;12(2):e047843. doi: 10.1136/bmjopen-2020-047843 (PMC8808410; doi:10.1136/bmjopen-2020-047843)
Supplement: Supplementary data [file bmjopen-2020-047843supp001.pdf]

Supplemental Table 1. Loss-to-follow-up analysis of adolescent girls and young women interviewed at Round 1 in Kenya, Malawi, and Zambia

Characteristics/Outcomes	Kenya				Malawi				Zambia			
	15-19		20-24		15-19		20-24		15-19		20-24	
	Followed n=389	Lost n=85	Followed n=347	Lost n=93	Followed n=371	Lost n=159	Followed n=883	Lost n=250	Followed n=487	Lost n=98	Followed n=398	Lost n=81
**Age**												
Mean (SD)	16.6 (1.3)	16.7 (1.3)	21.6 (1.4)	21.4 (1.4)	18.0 (1.2)	17.9 (1.2)	21.9 (1.4)	22.0 (1.5)	16.8 (1.4)	16.7 (1.4)	21.6 (1.3)	21.5 (1.3)
**Marital Status**												
Ever Married	4.4	4.7	44.4	35.5	62.3	55.4	88.9	86.8	2.5	2.0	8.5	6.2
Never married	95.6	95.3	55.6	64.5	37.7	44.6	11.1	13.2	97.5	98.0	91.5	93.8
**Current Schooling Status**	*											
In school	84.3	75.3	32.9	28.0	-	-	-	-	77.6	82.7	61.6	60.5
Out of school	15.7	24.7	67.1	72.0	-	-	-	-	22.4	17.3	38.4	39.5
**Orphanhood**	**											
Both parents alive	62.5	45.9	42.4	37.6	70.9	74.8	61.4	61.4	64.9	61.2	55.5	51.9
Lost at least one parent	37.5	54.1	57.6	62.4	29.1	25.2	38.6	38.8	35.1	38.8	44.5	48.1
**Site**					**		*		**			
Location 1	49.6	56.5	47.8	55.9	33.7	19.5	31.7	23.2	37.0	55.1	64.6	71.6
Location 2	50.4	43.5	52.2	44.1	66.3	80.5	68.3	76.8	63.0	44.9	35.4	28.4
**HIV Service Use**												
HIV test in last 12 months	**											
No	18.5	5.9	3.7	0.0	14.6	14.1	17.5	19.4	52.6	53.1	32.4	33.3
Yes	81.5	94.1	96.3	100.0	85.4	85.9	82.5	80.6	47.4	46.9	67.6	66.7
**Sexual Behavior**												

Experience of Sexually transmitted infection symptoms									*			
No	85.6	82.3	76.9	78.5	74.4	69.2	68.2	65.6	87.3	78.6	84.2	82.7
Yes	14.4	17.7	23.1	21.5	25.6	30.8	31.8	34.4	12.7	21.4	15.8	17.3
Number of sex partners									***			
	n=126	n=35	n=317	n=87	n=329	n=130	n=876	n=245	n=106	n=32	n=254	n=46
0	11.1	8.6	5.1	4.6	20.4	21.5	23.5	24.5	7.6	6.3	15.0	8.7
1	73.0	80.0	79.8	80.5	70.2	67.7	70.3	72.6	85.8	62.5	72.0	73.9
2+	15.9	11.4	15.1	14.9	9.4	10.8	6.2	2.9	6.6	31.3	13.0	17.4
Consistent condom use												
	n=59	n=19	n=218	n=59	n=238	n=92	n=684	n=184	n=40	n=8	n=97	n=16
No	47.5	47.4	69.3	66.1	88.2	91.3	95.0	94.0	57.5	50.0	57.7	56.3
Yes	52.5	52.6	30.7	33.9	11.8	8.7	5.0	6.0	42.5	50.0	42.3	43.8
Condom use at last sex												
	n=59	n=19	n=218	n=59	n=238	n=92	n=684	n=184	n=40	n=8	n=97	n=16
No	25.4	26.3	53.7	44.1	79.8	76.1	86.0	84.2	40.0	25.0	47.4	43.8
Yes	74.6	73.7	46.3	55.9	20.2	23.9	14.0	15.8	60.0	75.0	52.6	56.3
Transactional sex									*			
	n=126	n=35	n=317	n=87	n=329	n=130	n=876	n=245	n=107	n=32	n=254	n=46
No	90.5	85.7	94.3	94.3	97.6	96.2	96.1	96.7	99.1	90.6	97.6	97.8
Yes	9.5	14.3	5.7	5.7	2.4	3.9	3.9	3.3	0.9	9.4	2.4	2.2
**Violence perpetrated by an intimate partner**	n=197	n=48	n=257	n=72	n=338	n=143	n=878	n=244	n=296	n=65	n=347	n=68

Experience of sexual violence from partners							*		*			
No	80.2	87.5	79.8	81.9	83.1	81.1	83.6	77.1	82.4	69.2	81.6	72.1
Yes	19.8	12.5	20.2	18.1	16.9	18.9	16.4	22.9	17.6	30.8	18.4	27.9
Experience of physical violence from partners												
No	84.8	83.3	68.9	70.8	86.1	86.0	82.8	85.3	76.7	75.4	84.4	76.5
Yes	15.2	16.7	31.1	29.2	13.9	14.0	17.2	14.8	23.3	24.6	15.6	23.5
**Violence perpetrated by a non-partner**												
Experience of sexual violence from non-partners			*									
No	78.9	78.8	69.5	81.7	90.0	88.1	91.5	91.6	87.5	84.7	76.6	76.5
Yes	21.1	21.2	30.5	18.3	10.0	12.0	8.5	8.4	12.5	15.3	23.4	23.5

*p≤0.05; **p≤0.01; ***p≤0.001
