# Supplementary material for: Temporal shifts in HIV-related risk factors among cohorts of adolescent girls and young women enrolled in DREAMS programming: evidence from Kenya, Malawi and Zambia
Source: BMJ Open. 2022 Feb 1;12(2):e047843. doi: 10.1136/bmjopen-2020-047843 (PMC8808410; doi:10.1136/bmjopen-2020-047843)
Supplement: Author's manuscript [file bmjopen-2020-047843.draft_revisions.pdf]

BMJ Open

BMJ Open is committed to open peer review. As part of this commitment we make the peer review history of every article we publish publicly available.

When an article is published we post the peer reviewers' comments and the authors' responses online. We also post the versions of the paper that were used during peer review. These are the versions that the peer review comments apply to.

The versions of the paper that follow are the versions that were submitted during the peer review process. They are not the versions of record or the final published versions. They should not be cited or distributed as the published version of this manuscript.

BMJ Open is an open access journal and the full, final, typeset and author-corrected version of record of the manuscript is available on our site with no access controls, subscription charges or pay-per-view fees (http://bmjopen.bmj.com).

If you have any questions on BMJ Open's open peer review process please email info.bmjopen@bmj.com

BMJ Open

Examining temporal shifts in HIV-related risk factors among cohorts of adolescent girls and young women enrolled in DREAMS programming in Kenya, Malawi, and Zambia

Journal:	*BMJ Open*
Manuscript ID	bmjopen-2020-047843
Article Type:	Original research
Date Submitted by the Author:	11-Dec-2020
Complete List of Authors:	Mathur, Sanyukta; Population Council<br>Heck, Craig ; Population Council<br>Kishor Patel, Sangram; Population Council India<br>Okal, Jerry; Population Council Kenya, HIV/AIDS<br>Chipeta , Effie; University of Malawi College of Medicine, Centre for Reproductive Health<br>mwapasa, victor; University of Malawi College of Medicine, Centre for Reproductive Health<br>Chimwaza, Wanangwa; University of Malawi College of Medicine, Centre for Reproductive Health<br>Musheke, Maurice; Independent Consultant<br>Mahapatra, Bidhubhusan ; Population Council India<br>Pulerwitz, Julie ; Population Council<br>Pilgrim, Nanlesta; ViiV Healthcare US
Keywords:	Public health < INFECTIOUS DISEASES, HIV & AIDS < INFECTIOUS DISEASES, Epidemiology < INFECTIOUS DISEASES

SCHOLARONE™
Manuscripts

I, the Submitting Author has the right to grant and does grant on behalf of all authors of the Work (as defined in the below author licence), an exclusive licence and/or a non-exclusive licence for contributions from authors who are: i) UK Crown employees; ii) where BMJ has agreed a CC-BY licence shall apply, and/or iii) in accordance with the terms applicable for US Federal Government officers or employees acting as part of their official duties; on a worldwide, perpetual, irrevocable, royalty-free basis to BMJ Publishing Group Ltd ("BMJ") its licensees and where the relevant Journal is co-owned by BMJ to the co-owners of the Journal, to publish the Work in this journal and any other BMJ products and to exploit all rights, as set out in our licence.

The Submitting Author accepts and understands that any supply made under these terms is made by BMJ to the Submitting Author unless you are acting as an employee on behalf of your employer or a postgraduate student of an affiliated institution which is paying any applicable article publishing charge ("APC") for Open Access articles. Where the Submitting Author wishes to make the Work available on an Open Access basis (and intends to pay the relevant APC), the terms of reuse of such Open Access shall be governed by a Creative Commons licence – details of these licences and which Creative Commons licence will apply to this Work are set out in our licence referred to above.

Other than as permitted in any relevant BMJ Author's Self Archiving Policies, I confirm this Work has not been accepted for publication elsewhere, is not being considered for publication elsewhere and does not duplicate material already published. I confirm all authors consent to publication of this Work and authorise the granting of this licence.

For peer review only

Examining temporal shifts in HIV-related risk factors among cohorts of adolescent girls and young women enrolled in DREAMS programming in Kenya, Malawi, and Zambia

Authors:

Sanyukta Mathur[1]*, Craig J. Heck[2], Sangram Kishor Patel[3], Jerry Okal[4], Effie Chipeta[5], Victor Mwapasa[5], Wanangwa Chimwaza[5], Maurice Musheke[6], Bidhubhusan Mahapatra[3], Julie Pulerwitz[1], Nanlesta Pilgrim[7]

Affiliations:

1. Population Council, Washington, DC, United States of America
2. Population Council, New York, NY, United States of America
3. Population Council, New Delhi, India
4. Population Council, Nairobi, Kenya
5. Centre for Reproductive Health, College of Medicine, Blantyre, Malawi
6. Independent consultant, Lusaka, Zambia
7. ViiV Healthcare, Research Triangle Park, NC, United States of America

Corresponding author:

Sanyukta Mathur
4301 Connecticut Avenue, NW
Suite 280
Washington, DC 20008
E-mail: smathur@popcouncil.org
Telephone: +1 202–237–9447
Fax: +1 202-237-8410

Keywords: HIV prevention, longitudinal analysis, trend decomposition, multi-sectoral, risk behaviors

Word count: 5039 (inclusive of in-text citations)

For peer review only

Abstract (297/300)

Objectives: To assess temporal shifts in HIV risk factors among adolescent girls (AG, aged 15-19 years) and young women (YW, aged 20-24 years) in Kenya, Malawi, and Zambia.

Design: Prospective cohorts with two timepoints (Kenya: 2016, 2018; Malawi: 2017, 2018; Zambia: 2016/2017, 2018)

Setting: Community-based programming

Participants: 1247 AG (Kenya: 389, Malawi: 371, Zambia: 487) and 1628 YW (Kenya: 347, Malawi: 883, Zambia: 398)

Intervention: DREAMS, a multi-sectoral approach to reduce AGYW's HIV vulnerability by delivering a package of tailored, multi-layered activities and services

Primary and secondary outcome measures: HIV testing, sexual debut, STI symptom experience, number of sexual partners, condom use (consistently, at last sex), transactional sex, experience of physical violence (from intimate partners) and sexual violence (from intimate partners and strangers/non-partners)

Results: Changes in HIV-related risk behaviors among DREAMS participants varied by age group and country. Among AG, HIV testing increased, more were sexually active (Kenya and Zambia), and sexual violence from partners (in Kenya and Malawi) and non-partners (in Malawi) decreased. Among YW, HIV testing increased and STI experience decreased in Malawi; consistent condom use decreased in Kenya; transactional sex increased in Kenya and Zambia; and physical violence (in Malawi) and sexual violence from partners (in Kenya and Malawi) and non-partners (all 3 countries) decreased over time. Getting married and/or leaving school

For peer review only

contributed to shifts in sexual debut (Kenyan and Zambian AG), inconsistent condom use (Kenyan YW), and non-partner sexual violence (Zambian YW).

Conclusions: Improvements in HIV testing and reductions in experiences of sexual violence were coupled with variable shifts in HIV-related risk behaviors among DREAMS participants in Kenya, Malawi, and Zambia. Life transitions (marriage and leaving school) can affect AGYW's risk circumstances, highlighting the need for further tailoring HIV prevention programming to address the risk heterogeneity among AG and YW across different contexts.

For peer review only

Strengths and limitations of this study

- Longitudinal cohorts of adolescent girls and young women in three sub-Saharan countries were followed across two time points, over a 12-16-month time period
- Comprehensive quantitative survey captured knowledge, attitudes, and HIV-related risk behaviors and exposure to a community-based multi-sectoral HIV prevention program
- Multivariate analyses examined change over time in factors associated with HIV acquisition
- Decomposition analysis was used to assess if sociodemographic shifts influenced temporal changes
- Lack of a comparison group limits the generalizability of our findings

For peer review only

INTRODUCTION

Four decades into the HIV epidemic, adolescent girls and young women (AGYW, aged 15–24 years) remain at high risk for HIV. Globally, over 7,000 AGYW seroconvert weekly, and even though they comprise one-tenth of the population, 20% of sub-Saharan Africa's (SSA) seroconversions occur amongst AGYW.[1] In eastern and southern Africa, which has the world's highest regional prevalence (7.0% [5.9%-7.9%]),[2] the HIV epidemic is magnified by gendered disparities. AGYW's prevalence and incidence rates in Kenya, Malawi, and Zambia are double that (or more) of their male counterparts,[3-5] and when compared with all AGYW in SSA, all three countries have a prevalence that is equal to or greater than the regional average.[6] Though adolescent girls (AG, aged 15-19 years) and YW (YW, aged 20-24 years) are, collectively, a priority population, the breadth, severity, and context of their HIV risk is not homogenous, for among females aged 15-29, HIV incidence is highest among AG in Kenya and YW in Malawi and Zambia.[6]

Underscoring these disproportionate HIV rates, a range of behavioral, biological, psycho-social, and structural factors fuel AGYW's vulnerability to HIV.[6] For instance, AGYW who have multiple sex partners are at higher risk for HIV due to increased exposure, [7 8] and experience of STI symptoms has been linked to risky behaviors and biological vulnerability.[7 9] Physical and sexual violence, which is known to cause physical and psychological trauma, is associated with AGYW's HIV risk when perpetrated by partners[10-13] and non-partners.[11 13] In addition, non-marital partnerships are associated with age-disparate relationships and inconsistent condom use among AGYW and sexual concurrency among their male partners.[14-16] Further, economic disenfranchisement and limited social capital push some AGYW to seek out sexual partners for financial support; these transactional arrangements, often, include relationships with older men[17 18] and increase AGYW's HIV risk.[19-21] Negative life and social transitions—such as experiencing paternal loss,[22-25] divorce/widowhood,[7 26] and school dropout[7 18 27]—

For peer review only

have also been associated with heighted engagement in risky sexual behaviors and precarious decision-making.

AGYW's HIV risk factors are multi-faceted and ecological and, thus, are difficult to positively change using siloed, uni-component interventions.[28] In response, the U.S. President's Emergency Plan for AIDS Relief (PEPFAR), the Bill & Melinda Gates Foundation, Girl Effect, Johnson & Johnson, Gilead Sciences, and ViiV Healthcare initiated the Determined, Resilient, Empowered, AIDS-free, Mentored and Safe (DREAMS) Partnership in 2015, with the goal to reduce HIV acquisition among AGYW in 14 sub-Saharan countries (including Kenya, Malawi, and Zambia) and Haiti. DREAMS delivers, through public and private sectors, a comprehensive package of evidence-based strategies that aimed to reach vulnerable AGYW, and provide them with knowledge, life skills, and access to social protection interventions (e.g. educational support), as well as engage parents and the broader community to create an enabling environment to support AGYW health and development.[29-32] However, there is limited evidence about how this AGYW-focused approach, which extends beyond behavioral or health sector interventions, is influencing HIV-related risk among AGYW.

To contribute to the growing body of literature around comprehensive multi-sectoral HIV prevention programs, we assess temporal shifts in factors that have been previously linked to HIV-acquisition among DREAMS participants across three country contexts. Using an age-stratified (AG and YW) approach to illuminate risk heterogeneities, we examine change over time in proximal behavioral, biological, and experiential HIV-related risk factors to assess if participation in the DREAMS program positively influenced participants' vulnerability. To strengthen our investigation, we use decomposition analysis to elucidate if shifts in sociodemographic characteristics attribute to the observed changes in HIV-related risk factors. This study provides initial insight on the change over time in HIV-related risk factors among cohorts of AG and YW DREAMS participants in Kenya, Malawi, and Zambia, with the aim to inform the refinement or development of comprehensive multi-sectoral HIV prevention efforts.

For peer review only

METHODS

Program description

The DREAMS program aims to empower AGYW and reduce their risk of HIV and gender-based violence by delivering a package of tailored, multi-layered activities, services, and interventions.[29-32] In brief, the DREAMS program promotes positive sexual health behaviors; imparts knowledge and skills to increase self-efficacy; provides education subsidies and combination socioeconomic approaches; increases AGYW's access to sexual and reproductive health services; and creates an enabling environment for AGYW by supporting caregivers and addressing harmful community norms and practices. The program uses a safe spaces platform to engage AGYW in the DREAMS activities. At the safe spaces, AGYW socialize with peers and mentors, participate in facilitated instruction on health and life skills, and receive referrals to youth-friendly health services and social protection interventions.

PEPFAR determined the target DREAMS program areas, with input from local officials and other stakeholders, based on their high HIV prevalence and additional factors, such as high poverty levels, population density, and/or population growth. In each site, implementing partners (IP) used systematic selection processes (e.g., visiting and screening households in their community catchment area[33] to identify, recruit, and enroll eligible AGYW beneficiaries.[34-39] In Malawi, an additional eligibility criterion was out-of-school status. In each country context, a primary package of interventions[37 39 40] was delivered, with the expectation that all DREAMS participants would receive this primary package and additional interventions, depending on individual need or vulnerability.

Study sites

In Kenya and Zambia, the sites for this study were purposively selected, in consultation with PEPFAR, local stakeholders, and implementing partners, to be representative of key geographic characteristics (e.g.,

For peer review only

urban/peri-urban/rural) of DREAMS program communities in each country. In Malawi, the study sites were selected from each of the two districts involved in DREAMS programming at the time. In total, the study was conducted in two sites in Kisumu County, Kenya; four sites in Malawi's Zomba and Machinga districts; and two sites in Lusaka and Ndola, Zambia. At round one, study participants were identified using an age-stratified random sample from the DREAMS program beneficiary rosters (Kenya [n=5997], Malawi [proportionate to the differently sized districts]) prepared by the program IPs, whereas a census of all AGYW enrolled in DREAMS was conducted in Zambia. Using a conservative estimate (i.e., 50%) of baseline prevalence for the primary outcomes (e.g., HIV testing), the samples were powered at 80% to detect a minimal 10-20% percentage change.

Study population

Eligible survey participants were females aged 15–24 years residing in the study catchment area who was enrolled in the DREAMS program and intended to stay in the area for the subsequent year. Round one data was collected in 2016/2017. In Kenya, 474 AG and 440 YW were interviewed from October 2016 to February 2017. In Malawi, 528 AG and 1134 YW were interviewed from July 2017 to September 2017. In Zambia, 585 AG and 479 YW were interviewed from November 2016 to April 2017. Twenty respondents in Kenya, 33 in Zambia, and 3 in Malawi refused to participate due to lack of parental consent, in ability to locate the participant or limited time availability/other events that prohibited participation.

A second round of data was collected with round one respondents in 2018 after approximately 12-16 months of exposure to DREAMS. At round 2, 80.5% of Kenya's respondents (389 AG and 347 YW) were re-interviewed from April-June 2018, 75.5% of Malawi's respondents (371 AG and 883 YW) were re-interviewed from September-November 2018, and 79.8% of Zambia's respondents (487 AG and 398 YW) were re-interviewed from April-May 2018. Overall, loss to follow-up occurred due to extended or permanent out-migration from the study communities, as confirmed by the program team and through

For peer review only

repeated attempts to recontact participants by the research team. Attriters in Kenya were more likely to be out of school ($p<0.05$) and orphaned ($p<0.01$) than who remained in the study, while those lost to follow up in Malawi were more likely to be never married ($p<0.01$) and from Machinga ($p<0.001$). For Zambia, those not re-interviewed had re-located to towns outside study settings due to family, business or school reasons ($p<0.001$).

Study procedures

Respondent consent (or parent/guardian consent and minor assent) was obtained before each round of data collection. Using a tablet-based survey instrument in the respondent's chosen language (Kiswahili, and Luo [Kenya]; Chichewa and Yao [Malawi]; Bemba and Nyanja [Zambia]; or English [all countries]), female interviewers captured respondents' sociodemographic characteristics; program engagement and exposure; and knowledge, attitudes, and behaviors related to HIV/AIDS. Interviews were conducted in private yet convenient locations to the respondents (e.g., room in respondent's home, nearby field, or nearby community center), and out of earshot of parents, guardians, or other community members.

Measures

Cohorts' characteristics

The study cohorts only included respondents who provided information at both round one and two. We explored the cohorts' socio-demographic characteristics—such age (15-19 vs. 20-24) and marital (ever [currently, formerly] vs. never married), schooling (in school vs. out of school, Kenya and Zambia only), and orphanhood (both parents alive vs. at least one parent deceased) status, and using round 2 data only, we examined their participation in social asset building activities and exposure to (i.e., being offered the service/activity by DREAMS staff) various DREAMS program components—such as HIV testing services (HTS), health services (condoms, PrEP [where available], post-violence care, contraception, STI testing, pregnancy consultation), educational support (money for fees, uniforms, transport, or other help

For peer review only

with schooling expenses [Kenya, Zambia] or back to school support [Malawi]), and economic support (cash transfer [Kenya, Zambia] or food support [Malawi]). We did not change the study site (location 1 vs. location 2) variable (Kenya: Nyalenda A, Kolwa East; Malawi: Zomba, Machinga; Zambia: Lusaka, Ndola).

We assessed engagement in DREAMS' primary intervention by asking respondents if they participated in the safe space groups (yes vs. no) by round 2. Additionally, to examine the level of exposure to all DREAMS activities, we asked participants about the frequency and duration (short [<1 week, 1 week] vs. long [1 month, several months]) of their program interruptions. Among those that experienced periods of non-participation, we used the median number of interruptions (3 in all countries) to classify respondents as experiencing few (<median) or many (≥median) interruptions.

Outcomes

We focused on factors that are known to facilitate or mitigate AGYW's HIV risk. HIV testing in the last 12 months, sexual debut, and experiencing STI symptoms (genital warts, painful urination, vaginal discharge, genital ulcer) in the last 6 months were coded as binary (yes vs. no) variables. Among those who had sexually debuted, we truncated respondents' number of sexual partners in the last 12 months into a categorical variable (0, 1, 2+). We considered respondents to be consistent condom users if they were currently in a sexual relationship and in the last 12 months, they always used condoms with their primary and (if applicable) secondary partner; using this same schema, we also assessed condom use at last sex. For Zambia only, the condom use variables were only asked of respondents who were currently sexually active with their primary and (if applicable) secondary partner.

Using a binary (yes vs. no) variable, we identified respondents as transactional sex engagers if they had engaged in sex with a stranger or casual partner to receive cash or kind; somewhere to stay; support or money for their children or family; drugs, food, cosmetics, clothes, a cellphone, airtime, transportation;

For peer review only

somewhere to sleep for the night; financial support for bills or school fees; or anything else the they could not afford by themselves in the past 12 months.[41-43]

We measured intimate partner violence (IPV) among all respondents who reported having a sexual partner in the last 12 months and classified respondents as having experienced sexual IPV if they disclosed that in the last 12 months, they had sex or performed other sexual acts against their will because a current or previous boyfriend or partner used physical coercion, threats and intimation, or force. We coded respondents as having experienced physical IPV if they had been slapped or had something thrown at them; pushed or shoved; hit with a fist or another dangerous object; kicked, dragged, beaten, choked, or burnt; or threatened with a gun, knife, or other weapon by a current or previous boyfriend or partner in the last 12 months.[11]

Lastly, questions on non-partner sexual violence were asked of all AGYW. We classified respondents as having experienced non-partner sexual violence if they disclosed that in the last 12 months, a person other than their boyfriend or partner perpetrated unwanted sex by using persuasion or force—whether successful or unsuccessful; forced sex while they were under the influence of drugs or alcohol and too impaired to refuse; or forced sex by two or more men—when the respondent was either sober or under the influence of drugs or alcohol.[11]

Ethics

At each survey round, we collected written informed consent from adult participants (those above the age of consent in each country) and emancipated minors (when applicable). If the participant was considered a minor, we obtained parental consent and participant assent. The protocols for these studies were reviewed and approved by Population Council's Institutional Review Board (protocol # 744 and 745); Kenya's Kenyatta National Hospital/University of Nairobi Ethics (P384/05/2016) and Research Committee and National Commission for Science, Technology, and Innovation; Malawi's College of Medicine Research and Ethics Committee at the University of Malawi (PP.01/17/2095); and Zambia's

For peer review only

ERES Converge Institutional Review Board (2016- May-016) and National Health Research Authority. Participants were compensated (300 Kenyan Shilling [~USD $3], 1500 Malawian Kwacha [~ USD $2], 50 Zambian Kwacha [~USD $5]) for their time, per local ethical guidelines. Participation in this study did not influence access to or participation in any of the DREAMS interventions.

Data analysis

We conducted bivariate chi-squared analyses to examine the change in outcome measures between data collection rounds. To account for within-group correlation, we used generalized estimating equations (with a Poisson distribution) to examine temporal changes, control for cohorts' round one characteristics (interruptions in program participation [round two only], study site, and marital, schooling [Kenya and Zambia only], and orphanhood status), and report measures of association (Adjusted Incident Rate Ratio [95% Confidence Interval]). Additionally, we performed multivariate decomposition analyses to assess if shifts in key socio-demographic characteristics—schooling (Kenya and Zambia only), marital, and orphanhood status—attributed to the observed change in outcome measures over time. Standard two-component multivariate decomposition models assessed change in characteristics between two points in time among AGYW enrolled in DREAMS. We ran all analyses using Stata (version 15.0).

Patients and public involvement

This study actively engaged the study community and program implementing partners in the research process. Approval for the study was sought from community leaders and representatives in each setting during study planning. Research questions and tools were vetting with program implementing partners prior to administration. Post data collection, dissemination meetings were conducted with program implementing partners and community leaders to share findings and implications for HIV prevention and other programming for adolescent girls and young women in the study communities. Short study results briefs and presentation slide decks were developed and

For peer review only

shared with program implementing partners and other collaborators for dissemination of research results in each setting with key leaders and decision-makers.

RESULTS

Cohorts' characteristics

Table 1 presents the socio-demographic characteristics of the cohorts in Kenya, Malawi, and Zambia. Overall, the mean baseline age was between 17 (Kenya, Zambia) and 18 (Malawi) for AG and about 22 for YW (all countries). At round one, most (>50%) Kenyan and Zambian respondents were unmarried. Conversely, marriage was common among Malawian AG (62%) and YW (89%) at round one. The majority of AG in Kenya and Zambia were in school (>60%) at round one, and over 50% of Zambian YW were still in school at Round 2. Across all socio-demographic characteristics, there were modest shifts over time, as only a few age groups in certain countries had significant increases in out-of-school (Kenyan AG, Zambian AG and YW) and ever-married (Malawian AG and YW) status.

Respondents had nearly universal exposure to DREAMS' primary social asset building intervention: safe space groups, and exposure to HTS was high among Kenyan AG (96.1%) and YW (91.4%) but lower among AG and YW in Malawi and Zambia. With the exception of Kenya, where approximately ≥50% of AG and YW were exposed to educational support or economic support, exposure to other health services, educational support, and economic support was low (e.g. no YW in Malawi and 17% in Zambia had received some economic support).

On average, 75% of respondents experienced some form of program interruption, with just under 2 in 3 reporting many (i.e., ≥3) interruptions that last less than a week or more than a month. In Zambia for instance, 75% of AG noted there were times when they were not able to participate in DREAMS with 29% reporting at least 5 interruptions lasting for a month or more. The main reasons for periods of non-participation included being away at school (Kenyan AG [58.9%]), sickness (Malawian AG [51.5%] and

For peer review only - http://bmjopen.bmj.com/site/about/guidelines.xhtml

294 YW [47.1%]) and being away from the community (Kenyan YW [32.9%] and Zambian AG [29.0%] and
295 YW [37.4%]).
296
297

For peer review only

Table 1: Characteristics of cohorts in Kenya, Malawi, and Zambia

Characteristics	Kenya				Malawi				Zambia			
	15-19 n=389		20-24 n=347		15-19 n=371		20-24 n=883		15-19 n=487		20-24 n=398	
	Round 1	Round 2	Round 1	Round 2	Round 1	Round 2	Round 1	Round 2	Round 1	Round 2	Round 1	Round 2
**Mean Age (SD)**	16.6 (1.3)	18.3 (1.8)	21.6 (1.4)	22.6 (1.9)	18.0 (1.2)	19.3 (1.3)	21.9 (1.4)	23.1 (1.6)	16.8 (1.4)	18.1 (1.6)	21.6 (1.3)	22.5 (1.4)
**Marital Status**					***		***					
Ever Married	4.4	6.4	44.4	49.6	62.3	74.4	88.9	93.7	2.5	3.1	8.5	11.8
Never married	95.6	93.6	55.6	50.4	37.7	25.6	11.1	6.3	97.5	96.9	91.5	88.2
**Current Schooling Status**	**								***		**	
In school	84.3	75.3	32.9	27.1	-	-	-	-	77.6	60.6	61.6	52.3
Out of school	15.7	24.7	67.1	72.9	-	-	-	-	22.4	39.4	38.4	47.7
**Orphanhood**												
Both parents alive	62.5	58.1	42.4	38.9	70.9	69.5	61.4	60.8	64.9	62.6	55.5	54.3
Lost at least one parent	37.5	41.9	57.6	61.1	29.1	30.5	38.6	39.2	35.1	37.4	44.5	45.7
**Program Participation/Exposure**												
Social Asset Building	-	91.3	-	92.2	-	96.5	-	97.3	-	90.4	-	91.5
Offered HTS	-	96.1	-	91.4	-	62.0	-	65.1	-	80.9	-	73.9
Offered other health service	-	40.2	-	44.4	-	29.1	-	30.5	-	36.2	-	34.4
Educational Support	-	66.8	-	57.6	-	1.4	-	1.1	-	46.4	-	21.9
Economic Support	-	49.6	-	57.6	-	1.0	-	0.0	-	25.1	-	16.6
**Interruptions**												
None	-	27.5	-	26.5	-	21.0	-	24.0	-	25.1	-	29.4
<3 interruptions of ≤1 week	-	6.9	-	6.3	-	9.7	-	7.1	-	5.5	-	6.5
<3 interruptions of ≥1 month	-	7.7	-	4.6	-	3.2	-	3.4	-	3.5	-	3.3
≥3 interruptions of ≤1 week	-	23.1	-	28.8	-	22.1	-	28.0	-	37.0	-	30.4
≥3 interruptions of ≥1 month	-	34.7	-	33.7	-	43.9	-	37.5	-	28.9	-	30.4

*p≤0.05; **p≤0.01; ***p≤0.001 for marital, schooling, and orphanhood status

Other health services includes condoms, PrEP (where available), post-violence care, contraception, STI testing, pregnancy consultation

Educational support includes money for fees, uniforms, transport or other help with schooling expenses (Kenya, Zambia) or back to school support (Malawi)

Economic support includes cash transfer (Kenya, Zambia) and food support (Malawi)

Program participation/exposure and interruptions measured only at Round 2

Malawi's sample only included out-of-school participants; hence, current schooling status is not reported for Malawi's cohorts

For peer review only

302 # Unadjusted and adjusted transitions in AG's HIV risk factors

303 Figure 1 displays the unadjusted changes in factors associated with HIV acquisition among AG. Across
304 all three countries, HIV testing increased, with Zambian AG reporting the greatest increase (47% to 74%
305 [p≤0.001]), and among Kenyan and Zambian AG, sexual debut increased over time (32% to 45%
306 [p≤0.001], 22% to 33% [p≤0.001], respectively). Aside from Malawian AG reporting fewer multiple
307 partnerships (9% to 2% [p≤0.001]), there were no significant shifts related to STI experience, number of
308 sex partners, condom use, and transactional sex. There were significantly fewer reports of sexual IPV
309 among AG in Kenya (20% to 6% [p≤0.001]) and Malawi (18% to 6% [p≤0.001]), and there were no
310 significant changes in physical IPV experience. Although non-partner sexual violence decreased across
311 all three countries, Malawian AG was the only group to report significantly fewer incidents (10% to 4%
312 [p≤0.01]).

313 **Table 2: Adjusted shifts in behavioral, biological, and experiential factors**
314 **related to adolescent girls' HIV risk**

	Kenya	Malawi	Zambia
**HIV Service Use**			
HIV Testing in last year	**1.18 (1.02-1.37)**	1.11 (0.95-1.29)	**1.56 (1.34-1.82)**
**Sexual Behavior**			
Sexually debuted	**1.37 (1.16-1.62)**	1.04 (0.95-1.14)	**1.52 (1.28-1.81)**
STI Symptoms	1.02 (0.71-1.47)	0.87 (0.67-1.14)	0.90 (0.64-1.26)
Number of sex partners	0.99 (0.81-1.23)	1.01 (0.86-1.17)	0.91 (0.73-1.16)
Consistent condom use	0.78 (0.52-1.18)	0.60 (0.32-1.11)	0.81 (0.44-1.51)
Condom use at last sex	0.80 (0.54-1.17)	1.01 (0.68-1.51)	0.85 (0.51-1.44)
Transactional sex	0.89 (0.46-1.71)	1.39 (0.63-3.11)	3.57 (0.42-30.31)
**Violence Perpetrated by an Intimate Partner**			
Sexual	**0.32 (0.18-0.56)**	**0.35 (0.21-0.57)**	1.03 (0.72-1.46)
Physical	0.96 (0.61-1.49)	0.80 (0.55-1.16)	1.03 (0.77-1.36)
**Violence Perpetrated by a Non-partner**			
Sexual	0.93 (0.69-1.25)	**0.43 (0.25-0.75)**	0.87 (0.61-1.23)

Adjusted Incidence Rate Ratios (adjusting for continuity of program exposure, location, and marital, schooling [except in Malawi], and orphanhood status) and 95% Confidence Intervals are presented in each cell

Bolded cells indicate p≤0.05

315

For peer review only

The adjusted changes in AGs' HIV risk factors are presented in Table 2. Over time, HIV testing increased among Kenyan and Zambian AG (1.18 [1.02-1.37] and 1.56 [1.34-1.82], respectively). Along with testing, sexual debut increased among AGs in Kenya (1.37 [1.16-1.62]) and Zambia (1.52 [1.28-1.81]), and the decrease in sex partners among Malawian AG lost statistical significance. In Kenya and Malawi, sexual IPV experiences decreased among AG (0.32 [0.18-0.56] and 0.35 [0.21-0.57], respectively), while Zambia had null findings; AG in Kenya and Zambia reported null findings for physical IPV experience, as well. Alternatively, non-partner sexual violence significantly decreased among Malawian AG (0.43 [0.24-0.75]).

Unadjusted and adjusted changes in YW's HIV risk factors

Figure 2 presents the unadjusted shifts in factors related to YW's HIV risk. HIV testing in the last 12 months increased among Malawian (82% to 92% [p≤0.001]) and Zambian (67 to 76% [p≤0.01]) YW; in Malawi, a slight uptick in sexual debut (99.2% to 99.9% [p≤0.05]) was coupled with a decrease in STI experience (32% to 22% [p≤0.001]) and multiple partnerships (6% to 3% [p≤0.001]). Except for the decrease in consistent condom use among Kenyan YW (31% to 19% [p≤0.01]), there were no significant shifts in condom use, and though transactional sex slightly decreased among Malawian YW, it significantly increased among YW in Kenya (6% to 10% [p≤0.05]) and Zambia (2% to 6% [p≤0.05]). YW in Kenya and Malawi reported significantly fewer sexual IPV incidents (20% to 11% [p≤0.001], 16% to 5% [p≤0.001], respectively), and Malawian YW also reported fewer cases of physical IPV (17% to 12% [p≤0.001]). Among YW, non-partner sexual violence significantly decreased (p≤0.001) in all three countries (Kenya: 31% to 14%, Malawi: 9% to 2%, Zambia: 23% to 14%).

Table 3: Adjusted shifts in behavioral, biological, and experiential factors related to young women's HIV risk

	Kenya	Malawi	Zambia
**HIV Service Use**			
HIV Testing in last year	1.01 (0.88-1.15)	**1.11 (1.01-1.22)**	1.13 (0.99-1.28)
**Sexual Behavior**			

For peer review only - http://bmjopen.bmj.com/site/about/guidelines.xhtml

Sexually debuted	1.01 (0.90-1.14)	1.01 (0.93-1.09)	1.09 (0.98-1.21)
STI Symptoms	0.91 (0.68-1.22)	**0.69 (0.60-0.81)**	1.01 (0.75-1.35)
Number of sex partners	1.00 (0.87-1.15)	1.08 (0.98-1.18)	0.94 (0.81-1.10)
Consistent condom use	**0.57 (0.40-0.81)**	0.92 (0.59-1.43)	0.74 (0.48-1.13)
Condom use at last sex	0.77 (0.59-1.00)	1.05 (0.81-1.37)	0.84 (0.58-1.19)
Transactional sex	**1.73 (1.03-2.89)**	0.76 (0.47-1.23)	**2.62 (1.02-6.75)**
**Violence Perpetrated by an Intimate Partner**			
Sexual	**0.51 (0.34-0.75)**	**0.27 (0.19-0.39)**	0.78 (0.55-1.11)
Physical	0.78 (0.60-1.02)	**0.68 (0.55-0.85)**	0.75 (0.52-1.09)
**Violence Perpetrated by a Non-partner**			
Sexual	**0.44 (0.32-0.61)**	**0.21 (0.13-0.36)**	**0.60 (0.44-0.82)**

Adjusted Incidence Rate Ratios (adjusting for continuity of program exposure, location, and marital, schooling [except in Malawi], and orphanhood status) and 95% Confidence Intervals are presented in each cell

Bolded cells indicate p≤0.05

For peer review only

The adjusted transitions in YW's HIV risk factors are presented in Table 3. Between survey rounds, HIV testing increased and experience of STI symptoms decreased among Malawian YW (1.11 [1.01-1.22] and 0.69 [0.60-0.81], respectively). Consistent condom use decreased among Kenyan YW (0.57 [0.40-0.81]), and the decrease in sex partners among Malawian YW became statistically null. By round 2, Kenyan and Zambian YW reported higher transactional sex engagement (1.73 [1.03-2.89] and 2.62 [1.02-6.75], respectively). Sexual IPV experiences decreased among YW in Kenya (0.51 [0.34-0.75]) and Malawi (0.27 [0.19-0.39]). Malawian YW's physical IPV experience decreased significantly (0.68 [0.55-0.85]), too. Respondents also reported large shifts in non-partner sexual violence; it significantly decreased among YW in Kenya (0.44 [0.32-0.61]), Malawi (0.21 [0.13-0.36]), and Zambia (0.60 [0.44-0.82]).

Trend decomposition

As per the decomposition analysis (Supplemental Table 1), changes in martial (transitioning from never to ever married) and schooling (shifting from in school to out of school) status contributed to the significant uptick of sexual debut among Kenyan and Zambian AG and the significant decrease in consistent condom use among Kenyan YW. Change in marital status contributed to the significant

decrease in non-partner sexual violence among Zambian YW, too. The examined socio-demographic factors did not contribute to shifts in HIV testing, experience of STI symptoms, transactional sex engagement, or sexual or physical IPV experience. Change in vulnerability due to parental loss did not significantly contribute to the shifts over time in HIV-related risk factors. Since program participation was only assessed at round 2, we were unable to assess trends associated with program participation.

DISCUSSION

We present one of the first multi-country analyses examining temporal changes in behaviors and experiences associated with HIV acquisition among cohorts of AG and YW enrolled in DREAMS in Kenya, Malawi, and Zambia. Our analysis uses prospective data and presents stratified analyses by age group. Our findings illuminate heterogeneous shifts in HIV related risk factors between AG and YW, and across countries. Over time, AG who were part of DREAMS report increases in HIV testing (Kenya and Zambia), initiated sexual activity (Kenya and Zambia) and decreases in sexual IPV (Kenya and Malawi) and non-partner sexual violence (Malawi). HIV testing increased and experience of STIs decreased among YW in Malawi. Additionally, YW report increases in transactional sex (Kenya and Zambia) and decreases in consistent condom use (Kenya). YW engaged in DREAMS also report lower sexual IPV (Kenya and Malawi), lower physical IPV (Malawi), and non-partner sexual violence (Kenya, Malawi, and Zambia). Some emerging research in Uganda report that DREAMS programming contributed to the reduction of sexual risk behaviors; though, the effects differed by age group.[44] While the DREAMS programming models included considerations for participant age (e.g. providing education support to AG, and livelihoods support to YW), our findings indicate that additional tailoring of comprehensive HIV prevention packages may be needed to address the risk heterogeneity of AG and YW across different contexts.[43]

Increases in HIV testing rates among Kenyan and Zambian AG and Malawian YW are promising as HIV testing is the first step in the HIV care cascade and remains a key prevention strategy. Our data show that close to 75% (or greater) of AG and YW self-reported testing for HIV in the last year. In comparison,

For peer review only

recent national averages of self-reported HIV testing are much lower (Kenya: 35% of AG, 64% of YW; Malawi: 28% of AG, 52% of YW; Zambia: 29% of AG, 53% of YW).[3 4 45] These results likely point to PEPFAR, local national HIV programs and policies, and DREAMS' focus on expanded access to HIV testing services to AGYW in the DREAMS study communities.[29] Though only observed within one group, it is also encouraging to see that Malawian YW reported fewer STI symptoms over time, as STIs increase AGs' and YW's biological vulnerability to HIV.[46 47] Within DREAMS, establishing, providing, and strengthening youth-friendly health services was a key part of the intervention package. AG and YW were primarily linked to these services through discussions and referrals provided in the safe space groups. This community-based platform may have been particularly helpful in bringing services to marginalized AGYW, who would otherwise be hard to reach with HIV and reproductive health programming.[48]

Decreases in experience of sexual IPV over time are also promising. Previous research supports the direct relationship between HIV acquisition and sexual IPV, primarily because it facilitates other risk behaviors and mechanisms (e.g., decrease in self-efficacy and increases in multiple partnerships, transactional sex, substance abuse, negative mental health outcomes, and biological vulnerability due to trauma).[10 11 49 50] Compared with data from nationally-representative surveys among ever-married women aged 15-49, the baseline prevalence for sexual IPV among AGYW in our cohorts are high.[45 51] The DREAMS programs were conducted in high-risk communities with the most-at-risk AGYW, which may account for the higher experiences of sexual violence than the national average. Over time, we find that AG and YW in Kenya and Malawi reported significantly fewer incidents of sexual IPV over time. Since it is also perpetrated by a trusted individual, physical IPV has similar mediating pathways.[10 52] In, almost, all countries and age groups, physical IPV reduced, yet the reduction was only significant among Malawian YW and could be because most Malawian YW were ever married at round one and, therefore, had the greatest opportunity for positive change; they also had the highest mean age, which could have translated into greater agency and relationship power. Additional evidence is needed to support or refute these hypotheses. Zambian AG

For peer review only

and YW reported no significant shifts related to either IPV outcome; this warrants further investigation as to how IPV is perceived among Zambian AGYW and how programs are attempting to address it.

Sexual violence perpetrated by non-partners (assault and rape) is understudied; in our analysis, the baseline prevalence of non-partner sexual violence ranged from 10.0% to 21.1% among AG and 8.5% to 30.5% among YW. These totals are likely underestimated due to the topic's sensitive nature, which can trigger recollections of sadness and trauma; regardless, compared with other non-partner sexual violence metrics (9.1% prevalence among Johannesburg AG,[13] 11.9% [8.5%-15.3%] lifetime prevalence among African women[52]), these baseline totals are high. Other studies have found that surviving non-partner sexual violence can lead to a myriad of behaviors or outcomes that heighten HIV risk (e.g., substance abuse, poorer sexual and reproductive health and mental health outcomes, and increased experience of STIs).[11 13 52] In our study, it is reassuring that we observed appreciable drops in non-partner sexual violence among DREAMS participants across all age groups and countries, which hopefully signifies community-wide shifts in accountability around violence and safer spaces for AGYW. DREAMS' multi-sectoral approach could explain some of the notable decrease in intimate and non-partner violence. Gender-inequitable social norms and patriarchal power structures, often, create environments where men are emboldened to perpetrate violence, both against their intimate and strangers/casual partners. To disrupt these norms, DREAMS programming made a concerted effort to create an enabling environment for AGYW by challenging existing power dynamics and addressing gender-based violence—both within the safe space groups and the broader programming community.[29-32] However, since all girls were exposed to anti-violence programming and our study does not include any data from men or other community members, this study cannot disentangle its effects from other activities. To strengthen our understanding, future research should consider collecting attitudes and behaviors related to non-partner violence from various ecological strata, and further examination is required to unpack the specific programming components and pathways that may have contributed to these significant changes.

For peer review only

We do not find substantial shifts in other preventive behaviors (i.e., increase in condom use, decrease in multiple partnerships), even though a number of respondents—primarily Kenyan and Zambian AG—sexually debuted over time. On average, consistent condom use and condom use at last sex decreased between survey rounds, with the greatest decrease occurring among Kenyan YW. While previous research shows that proper condom use is protective against HIV acquisition,[53-55] condom uptake among adolescent and youth populations is consistently suboptimal,[53 56-59] a pattern that is reinforced by our findings. Our trend decomposition analysis confirms prior research that condom use decreased as AG and YW entered martial partnerships. Previous literature has demonstrated that low condom use among AG and YW could be a product of failed negotiations fueled by low self-efficacy, relationship power, and gender-inequitable norms and practices—highlighting the need for additional female-controlled HIV prevention methods.[6 18 43 60 61]

Among Kenyan and Zambian YW, we found significantly higher reports of transactional sex over time. Previous research shows that transactional sex can increase women's HIV risk.[19 20 41 62 63] To avert poverty, homelessness, and food insecurity, some may turn to transactional sex to survive or to obtain desired commodities.[17 18 64] AGYW's economic disenfranchisement is characterized by a constellation of factors—including gendered discrimination and lack of social and business capital[65]—that make it difficult to AGYW to access the economic resources or earn a living wage. DREAMS provided some economic activities (e.g. financial literacy and education) and strengthened access to some economic resources (e.g. access to school bursary programs or savings accounts); it is possible that broader partnerships or deeper investments are needed to make gainful employment/entrepreneurship possible for AGYW. Other structural intervention programs have reported reductions in economically-driven sex, risky sexual practices (e.g., condomless sex, multiple sex partners).[66-69] These positive shifts show that intersectional approaches can lead to meaningful change and should continue be considered by future HIV prevention programs for AGYW.[70-72]

For peer review only

While we found some promising results, this study is not without limitations. Power was slightly reduced as 17-25% of the respondents could not be re-interviewed at round two; however, this level of attrition is consistent with other community-based safe space programs.[73] It is also likely that some of the most at-risk AGYW were not reached by our study or DREAMS programming. In Zambia, we could only assess condom use among a specific subset of sexually active respondents, thus our findings should not be generalized to all sexually active AGYW. All the data were self-reported, which is prone to social desirability and recall bias. An additional challenge was the lack of a control group; since the DREAMS program was implemented over time, it continued to enroll AGYW from nearby communities and catchment areas, making it hard to find an appropriate counterfactual group.

CONCLUSION

Recent large-scale HIV prevention efforts are currently underway to reduce the disproportionate burden of HIV among AGYW in sub-Saharan Africa. Our longitudinal analysis assessed temporal change in key behaviors and experiences associated with HIV acquisition among Kenyan, Malawian, and Zambian AGYW cohorts nested within DREAMS, a multi-sectoral HIV prevention program. These analyses found a positive effect on some HIV risk factors, such as HIV testing, non-partner sexual violence, and physical and sexual intimate partner violence, but variable improvements in sexual risk behaviors. Our findings contribute to the growing body of evidence that is assessing the impact of community-based multi-sectoral HIV prevention programming for AGYW.

For peer review only

Acknowledgements

The authors would like to acknowledge the implementing partners in Kenya (Aphia Plus/PATH, I Choose Life, Make Me Smile), Malawi (OneCommunity), and Zambia (PACT, CMMB, Marie Stopes Zambia), and our research team coordinators and data collectors for their support during the data collection. We would like to acknowledge support from Population Council colleagues who provided support in the preparation of this manuscript, and our deepest gratitude goes to the study participants. This work would not have been possible without the active support of the United States Agency for International Development Mission teams in Kenya, Malawi, and Zambia.

Contributors

SM, NP, and JP conceptualized the study. SM, NP, CJH, SKP, and BM devised and implemented the analysis plan. EC, VM, WC, JO, and MM supported the study design and led the data collection. SM and CJH led the manuscript writing. All authors reviewed and revised the manuscript.

Funding

Funding support for the Kenya and Zambia studies was provided by the Bill & Melinda Gates Foundation (OPP1136778, DREAMS Implementation Science Research). Funding for the Malawi study was provided by the generous support of the American people through the United States President's Emergency Plan for AIDS Relief (PEPFAR) and the United States Agency for International Development (USAID) under Project SOAR (Cooperative Agreement AID–OAA–A–14–00060). The contents of this manuscript are the sole responsibility of the authors and do not necessarily reflect the views of PEPFAR, USAID, or the United States Government.

Competing interests

NP works full-time for ViiV Healthcare. ViiV Healthcare did not fund the study. The contents of the manuscript do not reflect the views of ViiV Healthcare. SM, CJH, SKP, JO, EC, VM, WC, MM, BM, and JP declare no conflicts of interest.

For peer review only

Patient consent for publication

Not required

Provenance and peer review

Not commissioned, externally peer reviewed

Data availability statement

All the underlying data relevant to the study are fully available on Dataverse.

For peer review only

REFERENCES

1. UNAIDS. Women and HIV: A Spotlight on Adolescent Girls and Young Women: UNAIDS, 2019.

2. UNAIDS. People Living With HIV: HIV Prevalence 2018 [Available from: https://aidsinfo.unaids.org/ accessed June 12 2020.

3. Ministry of Health Zambia. Zambia Population-based HIV Impact Assessment (ZAMPHIA) 2016: Final Report: Ministry of Health, 2019.

4. Ministry of Health Malawi. Malawi Population-Based HIV Impact Assessment (MPHIA) 2015-2016: Final Report: Ministry of Health, 2018.

5. Ministry of Health Kenya: National AIDS Control Council. Kenya HIV Estimates: Report 2018, 2018.

6. UNAIDS. HIV prevention among adolescent girls and young women. Geneva, Switzerland, 2016.

7. Santelli JS, Edelstein ZR, Mathur S, et al. Behavioral, biological, and demographic risk and protective factors for new HIV infections among youth, Rakai, Uganda. *J Acquir Immune Defic Syndr* 2013;63(3):393.

8. Mabaso M, Sokhela Z, Mohlabane N, et al. Determinants of HIV infection among adolescent girls and young women aged 15–24 years in South Africa: a 2012 population-based national household survey. *BMC Public Health* 2018;18(1):183.

9. Pettifor AE, Rees HV, Kleinschmidt I, et al. Young people's sexual health in South Africa: HIV prevalence and sexual behaviors from a nationally representative household survey. *AIDS* 2005;19(14):1525-34.

10. Jewkes RK, Dunkle K, Nduna M, et al. Intimate partner violence, relationship power inequity, and incidence of HIV infection in young women in South Africa: a cohort study. *Lancet* 2010;376(9734):41-48.

11. Mathur S, Okal J, Musheke M, et al. High rates of sexual violence by both intimate and non-intimate partners experienced by adolescent girls and young women in Kenya and Zambia: Findings around violence and other negative health outcomes. *PLoS One* 2018;13(9)

For peer review only

12. Li Y, Marshall CM, Rees HC, et al. Intimate partner violence and HIV infection among women: a systematic review and meta-analysis. *J Int AIDS Soc* 2014;17(1):18845.

13. Decker MR, Peitzmeier S, Olumide A, et al. Prevalence and health impact of intimate partner violence and non-partner sexual violence among female adolescents aged 15–19 years in vulnerable urban environments: a multi-country study. *J Adolesc Health* 2014;55(6):S58-S67.

14. Balkus JE, Brown E, Palanee T, et al. An empiric HIV risk scoring tool to predict HIV-1 acquisition in African women. *J Acquir Immune Defic Syndr* 2016;72(3):333.

15. Mathur S, Wei Y, Zhong X, et al. Partner characteristics associated with HIV acquisition among youth in Rakai, Uganda. *J Acquir Immune Defic Syndr* 2015;69(1):75.

16. Harrison A, Cleland J, Frohlich J. Young people's sexual partnerships in KwaZulu-Natal, South Africa: patterns, contextual influences, and HIV risk. *Stud Fam Plann* 2008;39(4):295-308.

17. Wamoyi J, Buller AM, Nyato D, et al. "Eat and you will be eaten": a qualitative study exploring costs and benefits of age-disparate sexual relationships in Tanzania and Uganda: implications for girls' sexual and reproductive health interventions. *Reprod Health* 2018;15(1):207.

18. Chapman J, do Nascimento N, Mandal M. Role of Male Sex Partners in HIV Risk of Adolescent Girls and Young Women in Mozambique. *Glob Health Sci Pract* 2019;7(3):435-46.

19. Jewkes R, Dunkle K, Nduna M, et al. Transactional sex and HIV incidence in a cohort of young women in the stepping stones trial. *J AIDS Clin Res* 2012;3(5)

20. Kilburn K, Ranganathan M, Stoner MC, et al. Transactional sex and incident HIV infection in a cohort of young women from rural South Africa. *AIDS* 2018;32(12):1669.

21. Price JT, Rosenberg NE, Vansia D, et al. Predictors of HIV, HIV risk perception, and HIV worry among adolescent girls and young women in Lilongwe, Malawi. *J Acquir Immune Defic Syndr* 2018;77(1):53.

22. Birdthistle IJ, Floyd S, Machingura A, et al. From affected to infected? Orphanhood and HIV risk among female adolescents in urban Zimbabwe. *AIDS* 2008;22(6):759-66.

For peer review only

562 23. Karim QA, Kharsany AB, Leask K, et al. Prevalence of HIV, HSV-2 and pregnancy among high school students in rural KwaZulu-Natal, South Africa: a bio-behavioural cross-sectional survey. *Sex Transm Infect* 2014;90(8):620-26.

565 24. Nyirenda M, McGrath N, Newell M-L. Gender differentials in the impact of parental death: adolescent's sexual behaviour and risk of HIV infection in rural South Africa. *Vulnerable Child Youth Stud* 2010;5(3):284-96.

568 25. Operario D, Pettifor A, Cluver L, et al. Prevalence of parental death among young people in South Africa and risk for HIV infection. *J Acquir Immune Defic Syndr* 2007;44(1):93-98.

570 26. Nyato D, Materu J, Kuringe E, et al. Prevalence and correlates of partner violence among adolescent girls and young women: Evidence from baseline data of a cluster randomised trial in Tanzania. *PLoS One* 2019;14(10)

573 27. Hargreaves JR, Bonell CP, Boler T, et al. Systematic review exploring time trends in the association between educational attainment and risk of HIV infection in sub-Saharan Africa. *AIDS* 2008;22(3):403-14.

576 28. Toska E, Gittings L, Hodes R, et al. Resourcing resilience: social protection for HIV prevention amongst children and adolescents in Eastern and Southern Africa. *Afr J AIDS Res* 2016;15(2):123-40.

579 29. Saul J, Bachman G, Allen S, et al. The DREAMS core package of interventions: a comprehensive approach to preventing HIV among adolescent girls and young women. *PLoS One* 2018;13(12):e0208167.

582 30. Gourlay A, Birdthistle I, Mthiyane NT, et al. Awareness and uptake of layered HIV prevention programming for young women: analysis of population-based surveys in three DREAMS settings in Kenya and South Africa. *BMC Public Health* 2019;19(1):1417.

585 31. Office of the U.S. Global AIDS Coordinator and Health Diplomacy. Preventing HIV in adolescent girls and young women: guidance for PEPFAR country teams on the DREAMS partnership: President’s Emergency Plan for AIDS Relief, 2016.

For peer review only

32. Office of the U.S. Global AIDS Coordinator and Health Diplomacy. DREAMS core package of interventions summary: President's Emergency Plan for AIDS Relief, 2017.

33. The Girl Roster: A Practical Tool for Strengthening Girl-Centered Programming. New York, NY: The Population Council, 2019.

34. The United States Department of State. Kenya: DREAMS Overview (FY 2016-2021), 2020.

35. The United States Department of State. Malawi: DREAMS Overview (FY 2016-2021), 2020.

36. The United States Department of State. Zambia: DREAMS Overview (FY 2016-2021), 2020.

37. PATH. The Power of Dreams: HIV Prevention for AGYW in Western Kenya, 2019.

38. USAID. USAID/Zambia Health Office: Zambia Community HIV Prevention Project (Z-CHPP) 2020 [Available from: https://www.usaid.gov/zambia/fact-sheets/usaidzambia-health-office-zambia-community-hiv-prevention-project-z.

39. One Community. What We Do: DREAMS 2016 [Available from: http://www.onecommunitymw.org/index.php/what-we-do/dreams.

40. Pact. DREAMS and OVC, 2020.

41. Dunkle KL, Jewkes RK, Brown HC, et al. Transactional sex among women in Soweto, South Africa: prevalence, risk factors and association with HIV infection. *Soc Sci Med* 2004;59(8):1581-92.

42. Jewkes R, Nduna M, Levin J, et al. A cluster randomized-controlled trial to determine the effectiveness of Stepping Stones in preventing HIV infections and promoting safer sexual behaviour amongst youth in the rural Eastern Cape, South Africa: trial design, methods and baseline findings. *Trop Med Int Health* 2006;11(1):3-16.

43. Mathur S, Pilgrim N, Patel SK, et al. HIV vulnerability among adolescent girls and young women: a multi-country latent class analysis approach. *Int J Public Health* 2020:1-13.

44. Hadijja Nakawooya, Victor Ssempijja, Dorean Nabukalu, et al. PEPFAR DREAMS Intervention Among Adolescent Girls and Young Women in Rakai, Uganda 2020 [Available from: https://www.croiconference.org/abstract/pepfar-dreams-intervention-among-adolescent-girls-and-young-women-in-rakai-uganda/.

For peer review only

45. Kenya National Bureau of Statistics, Ministry of Health/Kenya, National AIDS Control Council/Kenya, et al. Kenya Demographic and Health Survey 2014. Rockville, MD, USA, 2015.

46. Kalichman SC, Pellowski J, Turner C. Prevalence of sexually transmitted co-infections in people living with HIV/AIDS: systematic review with implications for using HIV treatments for prevention. *Sex Transm Infect* 2011;87(3):183-90.

47. Mathur S, Mullinax M, Santelli JS. Prevention of sexually transmitted infections among adolescents. In: Cherry A., Baltag V., M. D, eds. International handbook on adolescent health and development: Springer 2017:183-205.

48. Denno DM, Chandra-Mouli V, Osman M. Reaching youth with out-of-facility HIV and reproductive health services: a systematic review. *J Adolesc Health* 2012;51(2):106-21.

49. Jewkes R, Gibbs A, Jama-Shai N, et al. Stepping Stones and Creating Futures intervention: shortened interrupted time series evaluation of a behavioural and structural health promotion and violence prevention intervention for young people in informal settlements in Durban, South Africa. *BMC Public Health* 2014;14(1):1325.

50. Stockman JK, Lucea MB, Campbell JC. Forced sexual initiation, sexual intimate partner violence and HIV risk in women: a global review of the literature. *AIDS Behav* 2013;17(3):832-47.

51. National Statistical Office/Malawi, ICF. Malawi Demographic and Health Survey 2015-16. Zomba, Malawi: National Statistical Office and ICF, 2017.

52. García-Moreno C, Pallitto C, Devries K, et al. Global and regional estimates of violence against women: prevalence and health effects of intimate partner violence and non-partner sexual violence: World Health Organization, 2013.

53. Thurman T, Kidman R, Carton T, et al. Psychological and behavioral interventions to reduce HIV risk: evidence from a randomized control trial among orphaned and vulnerable adolescents in South Africa. *AIDS Care* 2016;28(sup1):8-15.

54. Hallman K, Mubayiwa R, Madya S, et al. Program versus Comparison Endline Survey of the Mabinti Tushike Hatamu! (Girls Lets Be Leaders!) Programme in Tanzania: Population Council, The

For peer review only

640 CSR Group Africa Limited, Restless Development Tanzania, UNICEF Tanzania, Tanzania AIDS
641 Commission, 2016.
642 55. Davis KR, Weller SC. The effectiveness of condoms in reducing heterosexual transmission of HIV.
643 *Fam Plann Perspect* 1999:272-79.
644 56. Erulkar AS, Ettyang LI, Onoka C, et al. Behavior change evaluation of a culturally consistent
645 reproductive health program for young Kenyans. *Int Fam Plan Perspect* 2004:58-67.
646 57. Erulkar A, Chong E. Evaluation of a savings & micro-credit program for vulnerable young women in
647 Nairobi. Nairobi, Kenya, 2005.
648 58. Jewkes R, Nduna M, Levin J, et al. Impact of stepping stones on incidence of HIV and HSV-2 and
649 sexual behaviour in rural South Africa: cluster randomised controlled trial. *BMJ* 2008;337:a506.
650 59. George G, Maughan-Brown B, Beckett S, et al. Coital frequency and condom use in age-disparate
651 partnerships involving women aged 15 to 24: evidence from a cross-sectional study in KwaZulu-
652 Natal, South Africa. *BMJ Open* 2019;9(3):e024362.
653 60. Pulerwitz J, Mathur S, Woznica D. How empowered are girls/young women in their sexual
654 relationships? Relationship power, HIV risk, and partner violence in Kenya. *PLoS One*
655 2018;13(7)
656 61. Kalichman SC, Simbayi LC, Cain D, et al. Sexual assault, sexual risks and gender attitudes in a
657 community sample of South African men. *AIDS Care* 2007;19(1):20-27.
658 62. Rositch A, Cherutich P, Brentlinger P, et al. HIV infection and sexual partnerships and behaviour
659 among adolescent girls in Nairobi, Kenya. *Int J STD AIDS* 2012;23(7):468-74.
660 63. Ranganathan M, Heise L, Pettifor A, et al. Transactional sex among young women in rural South
661 Africa: prevalence, mediators and association with HIV infection. *J Int AIDS Soc*
662 2016;19(1):20749.
663 64. Gichane MW, Moracco KE, Pettifor AE, et al. Socioeconomic Predictors of Transactional Sex in a
664 Cohort of Adolescent Girls and Young Women in Malawi: A Longitudinal Analysis. *AIDS Behav*
665 2020

For peer review only

65. Stoebenau K, Wamoyi J, Fielding-Miller R, et al. Transactional sex and HIV risk, 2017.

66. Cluver LD, Orkin MF, Yakubovich AR, et al. Combination social protection for reducing HIV-risk behavior amongst adolescents in South Africa. *J Acquir Immune Defic Syndr* 2016;72(1):96.

67. Dunbar MS, Dufour M-SK, Lambdin B, et al. The SHAZ! project: results from a pilot randomized trial of a structural intervention to prevent HIV among adolescent women in Zimbabwe. *PLoS One* 2014;9(11):e113621.

68. Cluver L, Toska E, Orkin F, et al. Achieving equity in HIV-treatment outcomes: can social protection improve adolescent ART-adherence in South Africa? *AIDS Care* 2016;28(sup2):73-82.

69. Bandiera O, Buehren N, Burgess R, et al. Women's empowerment in action: evidence from a randomized control trial in Africa: World Bank, 2018.

70. STRIVE Research Consortium. Why STRIVE?: London School of Hygiene & Tropical Medicine, 2016.

71. STRIVE Research Consortium. About STRIVE 2020 [Available from: http://strive.lshtm.ac.uk/about.

72. Delany-Moretlwe S, Hargreaves J, Stangly A, et al. Biomedical and structural prevention: STRIVE in the era of 'cascades', 2018.

73. Temin M, Heck CJ. Close to Home: Evidence on the Impact of Community-Based Girl Groups. *Glob Health Sci Pract* 2020;8(2):300-24.

For peer review only

Figure 1: Bivariate Analyses of Change in Adolescent Girls' Behavioral, Biological, and Experiential HIV Risk Factors between Rounds 1 and 2

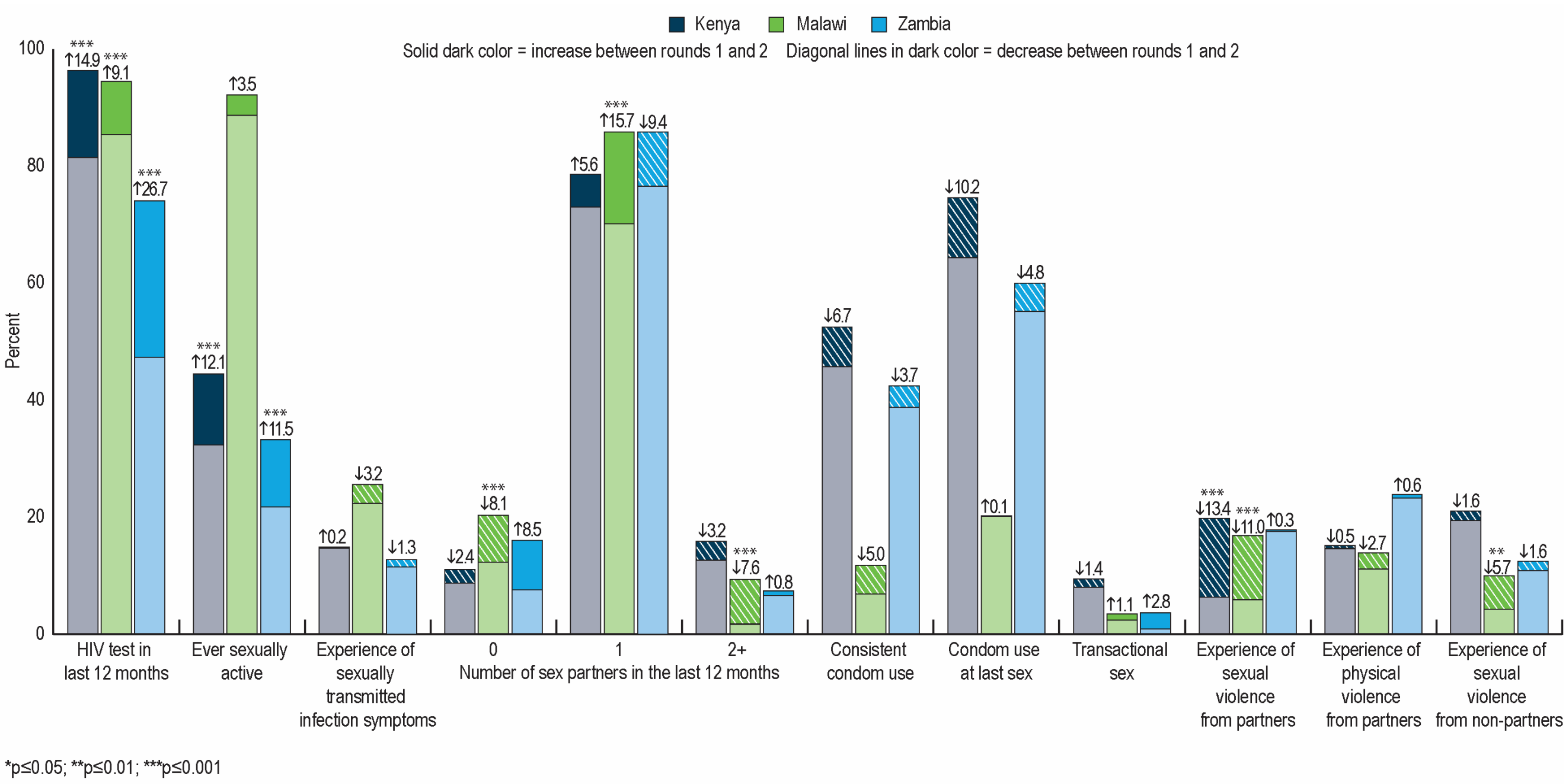

The number and notation atop each column indicate the direction and percentage-point change between Rounds 1 and 2.

Figure 2: Bivariate Analyses of Change in Young Women's Behavioral, Biological, and Experiential HIV Risk Factors between Rounds 1 and 2

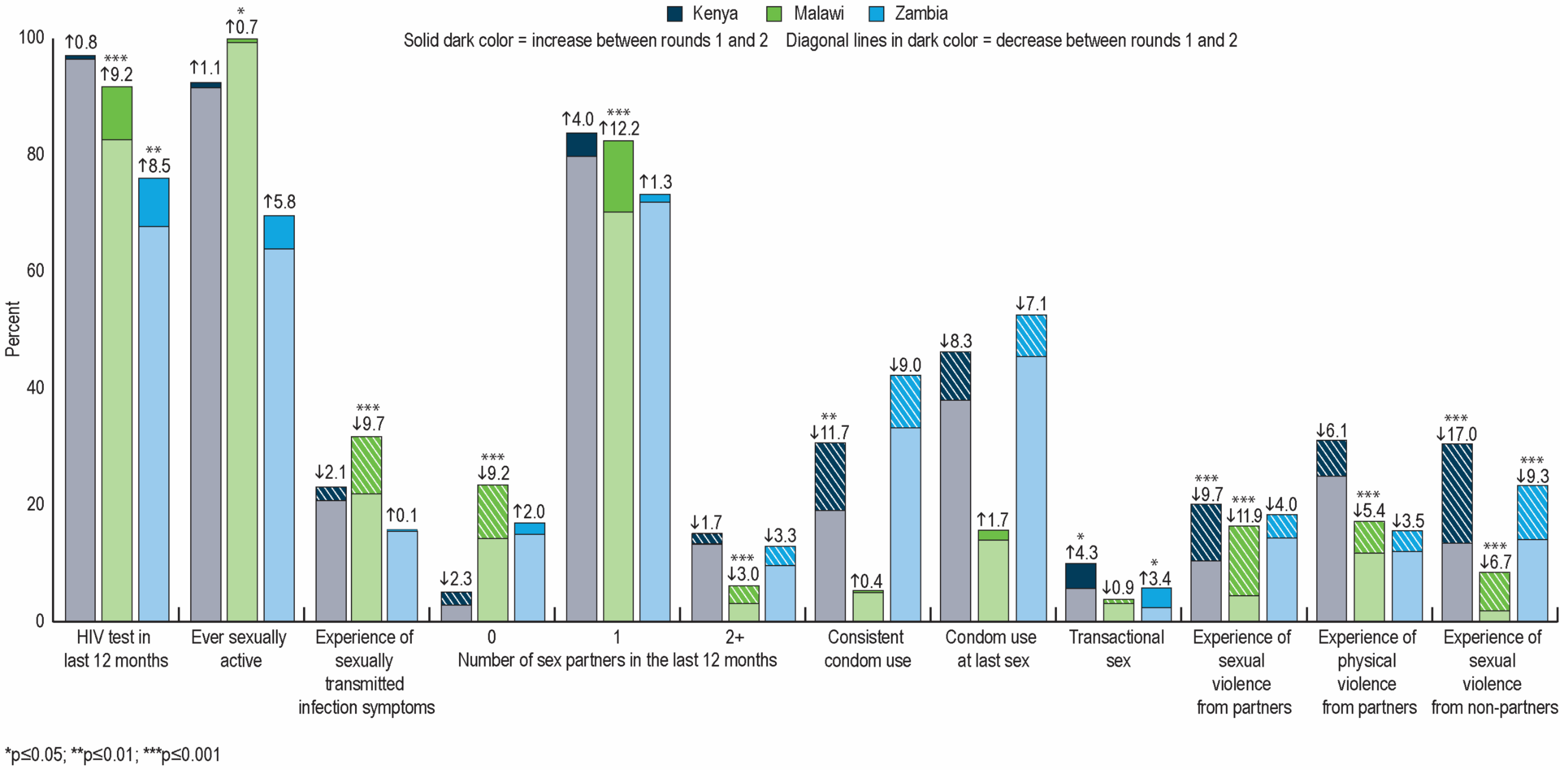

The number and notation atop each column indicate the direction and percentage-point change between Rounds 1 and 2.

Supplemental Table 1: Contribution of socio-demographic characteristics to the temporal changes in the outcome variables among AGYW in Kenya, Malawi, and Zambia

	Kenya		Malawi		Zambia	
	15-19	20-24	15-19	20-24	15-19	20-24
**HIV Service Use**						
HIV Testing in last year	-			-	-	
**Sexual Behavior**						
Sexually debuted	M, S				M, S	
STI Symptoms				-		
Number of sex partners						
Consistent condom use		M, S				
Condom use at last sex						
Transactional sex		-				-
**Violence Perpetrated by an Intimate Partner**						
Sexual	-	-	-	-		
Physical				-		
**Violence Perpetrated by a Non-partner**						
Sexual		-	-	-		M

Only decomposition findings for significant multivariable associations are presented

A dash (-) indicates that the multivariable association was significant, but the decomposition findings were null.

Key characteristics include marital, schooling (Kenya and Zambia only), and orphanhood status

M = change in marital status, S = change in schooling status, O = Change in orphanhood status

For peer review only

STROBE Statement—Checklist of items that should be included in reports of ***cohort studies***

	Item No	Recommendation	Page No
**Title and abstract**	1	(*a*) Indicate the study's design with a commonly used term in the title or the abstract	Title
		(*b*) Provide in the abstract an informative and balanced summary of what was done and what was found	1-2
**Introduction**			
Background/rationale	2	Explain the scientific background and rationale for the investigation being reported	4-5
Objectives	3	State specific objectives, including any prespecified hypotheses	5
**Methods**			
Study design	4	Present key elements of study design early in the paper	6
Setting	5	Describe the setting, locations, and relevant dates, including periods of recruitment, exposure, follow-up, and data collection	6-7
Participants	6	(*a*) Give the eligibility criteria, and the sources and methods of selection of participants. Describe methods of follow-up<br>(*b*) For matched studies, give matching criteria and number of exposed and unexposed	7-8
Variables	7	Clearly define all outcomes, exposures, predictors, potential confounders, and effect modifiers. Give diagnostic criteria, if applicable	8-10
Data sources/ measurement	8*	For each variable of interest, give sources of data and details of methods of assessment (measurement). Describe comparability of assessment methods if there is more than one group	8-10
Bias	9	Describe any efforts to address potential sources of bias	
Study size	10	Explain how the study size was arrived at	7-8
Quantitative variables	11	Explain how quantitative variables were handled in the analyses. If applicable, describe which groupings were chosen and why	8-10
Statistical methods	12	(*a*) Describe all statistical methods, including those used to control for confounding<br>(*b*) Describe any methods used to examine subgroups and interactions<br>(*c*) Explain how missing data were addressed<br>(*d*) If applicable, explain how loss to follow-up was addressed<br>(*e*) Describe any sensitivity analyses	11
**Results**			
Participants	13*	(a) Report numbers of individuals at each stage of study—eg numbers potentially eligible, examined for eligibility, confirmed eligible, included in the study, completing follow-up, and analysed<br>(b) Give reasons for non-participation at each stage<br>(c) Consider use of a flow diagram	12-14
Descriptive data	14*	(a) Give characteristics of study participants (eg demographic, clinical, social) and information on exposures and potential confounders<br>(b) Indicate number of participants with missing data for each variable of interest<br>(c) Summarise follow-up time (eg, average and total amount)	12-14
Outcome data	15*	Report numbers of outcome events or summary measures over time	15-17

For peer review only - http://bmjopen.bmj.com/site/about/guidelines.xhtml

Main results	16	(*a*) Give unadjusted estimates and, if applicable, confounder-adjusted estimates and their precision (eg, 95% confidence interval). Make clear which confounders were adjusted for and why they were included<br>(*b*) Report category boundaries when continuous variables were categorized<br>(*c*) If relevant, consider translating estimates of relative risk into absolute risk for a meaningful time period	15-17
Other analyses	17	Report other analyses done—eg analyses of subgroups and interactions, and sensitivity analyses	17-18
**Discussion**			
Key results	18	Summarise key results with reference to study objectives	18
Limitations	19	Discuss limitations of the study, taking into account sources of potential bias or imprecision. Discuss both direction and magnitude of any potential bias	22
Interpretation	20	Give a cautious overall interpretation of results considering objectives, limitations, multiplicity of analyses, results from similar studies, and other relevant evidence	18-21
Generalisability	21	Discuss the generalisability (external validity) of the study results	18-21
**Other information**			
Funding	22	Give the source of funding and the role of the funders for the present study and, if applicable, for the original study on which the present article is based	23

*Give information separately for exposed and unexposed groups.

Note: An Explanation and Elaboration article discusses each checklist item and gives methodological background and published examples of transparent reporting. The STROBE checklist is best used in conjunction with this article (freely available on the Web sites of PLoS Medicine at http://www.plosmedicine.org/, Annals of Internal Medicine at http://www.annals.org/, and Epidemiology at http://www.epidem.com/). Information on the STROBE Initiative is available at http://www.strobe-statement.org.

For peer review only

BMJ Open

Temporal shifts in HIV-related risk factors among cohorts of adolescent girls and young women enrolled in DREAMS programming: Evidence from Kenya, Malawi, and Zambia

Journal:	*BMJ Open*
Manuscript ID	bmjopen-2020-047843.R1
Article Type:	Original research
Date Submitted by the Author:	09-Aug-2021
Complete List of Authors:	Mathur, Sanyukta; Population Council<br>Heck, Craig ; Population Council<br>Kishor Patel, Sangram; Population Council India<br>Okal, Jerry; Population Council Kenya, HIV/AIDS<br>Chipeta , Effie; University of Malawi College of Medicine, Centre for Reproductive Health<br>mwapasa, victor; University of Malawi College of Medicine, Centre for Reproductive Health<br>Chimwaza, Wanangwa; University of Malawi College of Medicine, Centre for Reproductive Health<br>Musheke, Maurice; Independent Consultant<br>Mahapatra, Bidhubhusan ; Population Council India<br>Pulerwitz, Julie ; Population Council<br>Pilgrim, Nanlesta; ViiV Healthcare US
**Primary Subject Heading**:	HIV/AIDS
Secondary Subject Heading:	Epidemiology, Public health, Global health
Keywords:	Public health < INFECTIOUS DISEASES, HIV & AIDS < INFECTIOUS DISEASES, Epidemiology < INFECTIOUS DISEASES

SCHOLARONE™
Manuscripts

I, the Submitting Author has the right to grant and does grant on behalf of all authors of the Work (as defined in the below author licence), an exclusive licence and/or a non-exclusive licence for contributions from authors who are: i) UK Crown employees; ii) where BMJ has agreed a CC-BY licence shall apply, and/or iii) in accordance with the terms applicable for US Federal Government officers or employees acting as part of their official duties; on a worldwide, perpetual, irrevocable, royalty-free basis to BMJ Publishing Group Ltd ("BMJ") its licensees and where the relevant Journal is co-owned by BMJ to the co-owners of the Journal, to publish the Work in this journal and any other BMJ products and to exploit all rights, as set out in our licence.

The Submitting Author accepts and understands that any supply made under these terms is made by BMJ to the Submitting Author unless you are acting as an employee on behalf of your employer or a postgraduate student of an affiliated institution which is paying any applicable article publishing charge ("APC") for Open Access articles. Where the Submitting Author wishes to make the Work available on an Open Access basis (and intends to pay the relevant APC), the terms of reuse of such Open Access shall be governed by a Creative Commons licence – details of these licences and which Creative Commons licence will apply to this Work are set out in our licence referred to above.

Other than as permitted in any relevant BMJ Author's Self Archiving Policies, I confirm this Work has not been accepted for publication elsewhere, is not being considered for publication elsewhere and does not duplicate material already published. I confirm all authors consent to publication of this Work and authorise the granting of this licence.

For peer review only

Temporal shifts in HIV-related risk factors among cohorts of adolescent girls and young women enrolled in DREAMS programming: Evidence from Kenya, Malawi, and Zambia

Authors:

Sanyukta Mathur[1]*, Craig J. Heck[2], Sangram Kishor Patel[3], Jerry Okal[4], Effie Chipeta[5], Victor Mwapasa[5], Wanangwa Chimwaza[5], Maurice Musheke[6], Bidhubhusan Mahapatra[3], Julie Pulerwitz[1], Nanlesta Pilgrim[7]

Affiliations:

1. Population Council, Washington, DC, United States of America
2. Population Council, New York, NY, United States of America
3. Population Council, New Delhi, India
4. Population Council, Nairobi, Kenya
5. Centre for Reproductive Health, College of Medicine, Blantyre, Malawi
6. Independent consultant, Lusaka, Zambia
7. ViiV Healthcare, Research Triangle Park, NC, United States of America

Corresponding author:

Sanyukta Mathur
4301 Connecticut Avenue, NW
Suite 280
Washington, DC 20008
E-mail: smathur@popcouncil.org
Telephone: +1 202–237–9447
Fax: +1 202-237-8410

For peer review only

Abstract (299/300)

Objectives: To assess temporal shifts in HIV risk factors among adolescent girls (AG, aged 15-19 years) and young women (YW, aged 20-24 years) in Kenya, Malawi, and Zambia.

Design: Prospective cohorts with two timepoints (Kenya: 2016, 2018; Malawi: 2017, 2018; Zambia: 2016/2017, 2018)

Setting: Community-based programming

Participants: 1247 AG (Kenya: 389, Malawi: 371, Zambia: 487) and 1628 YW (Kenya: 347, Malawi: 883, Zambia: 398)

Intervention: DREAMS (Determined, Resilient, Empowered, AIDS-free, Mentored and Safe), a multi-sectoral approach to reduce AGYW's HIV vulnerability by delivering a package of tailored, multi-layered activities and services

Primary and secondary outcome measures: HIV testing, sexually transmitted infection (STI) symptom experience, number of sexual partners, condom use (consistently, at last sex), transactional sex, experience of physical violence (from intimate partners) and sexual violence (from intimate partners and strangers/non-partners)

Results: Changes in HIV-related risk behaviors among DREAMS participants varied by age group and country. Among AG, HIV testing increased, more were sexually active (Kenya and Zambia), and sexual violence from partners (in Kenya and Malawi) and non-partners (in Malawi) decreased. Among YW, HIV testing increased and STI experience decreased in Malawi; consistent condom use decreased in Kenya; transactional sex increased in Kenya and Zambia; and physical violence (in Malawi) and sexual violence from partners (in Kenya and Malawi) and

For peer review only

non-partners (all 3 countries) decreased over time. Getting married and/or leaving school contributed to shifts in inconsistent condom use (Kenyan YW) and non-partner sexual violence (Zambian YW).

Conclusions: Improvements in HIV testing and reductions in experiences of sexual violence were coupled with variable shifts in HIV-related risk behaviors among DREAMS participants in Kenya, Malawi, and Zambia. Additional consideration of AGYW's risk circumstances during key life transitions (leaving school, marriage, entering the labor force) may be needed to address the risk heterogeneity among AG and YW across different contexts.

For peer review only

54 # Strengths and limitations of this study

55 - Longitudinal cohorts of adolescent girls and young women in three sub-Saharan countries 56 were followed across two time points, over a 12-16-month time period
57 - Comprehensive quantitative survey captured knowledge, attitudes, and HIV-related risk 58 behaviors and exposure to a community-based multi-sectoral HIV prevention program
59 - Multivariate analyses examined change over time in factors associated with HIV 60 acquisition
61 - Due to a lack of a comparison group, our findings could have been confounded by 62 unobserved changes or secular trends in the study sites and/or the aging of the cohort

For peer review only

INTRODUCTION

Four decades into the HIV epidemic, adolescent girls and young women (AGYW, aged 15–24 years) remain at high risk for HIV. Globally, over 7,000 AGYW seroconvert weekly, and even though they comprise one-tenth of the population, 20% of sub-Saharan Africa's (SSA) seroconversions occur amongst AGYW.[1] In eastern and southern Africa, which has the world's highest regional prevalence (7.0% [5.9%-7.9%]),[2] the HIV epidemic is magnified by gendered disparities. AGYW's prevalence and incidence rates in Kenya, Malawi, and Zambia are double that (or more) of their male counterparts,[3-5] and when compared with all AGYW in SSA, all three countries have a prevalence that is equal to or greater than the regional average.[6] Though adolescent girls (AG, aged 15-19 years) and YW (YW, aged 20-24 years) are a priority population, the breadth, severity, and context of their HIV risk is not homogenous, for among females aged 15-29, HIV incidence is highest among AG in Kenya and YW in Malawi and Zambia.[6]

Underscoring these disproportionate HIV rates, a range of behavioral, biological, psycho-social, and structural factors fuel AGYW's vulnerability to HIV.[6] For instance, AGYW who have multiple sex partners are at higher risk for HIV due to increased exposure, [7 8] and experience of sexually transmitted infection (STI) symptoms has been linked to risky behaviors and biological vulnerability.[7 9] Physical and sexual violence, which is known to cause physical and psychological trauma, is associated with AGYW's HIV risk when perpetrated by partners[10-13] and non-partners.[11 13] In addition, non-marital partnerships are associated with age-disparate relationships and inconsistent condom use among AGYW and sexual concurrency among their male partners.[14-16] Further, economic disenfranchisement and limited social capital push some AGYW to seek out sexual partners for financial support; these transactional arrangements often include relationships with older men[17 18] and increase AGYW's HIV risk.[19-21] Negative life and social transitions—such as experiencing paternal loss,[22-25] divorce/widowhood,[7 26] and school dropout[7 18 27]—have also been associated with heighted engagement in risky sexual behaviors and precarious decision-making.

For peer review only

AGYW's HIV risk factors are multi-faceted and ecological, making them difficult to change using siloed, uni-component interventions.[28] In response, the U.S. President's Emergency Plan for AIDS Relief (PEPFAR), the Bill & Melinda Gates Foundation, Girl Effect, Johnson & Johnson, Gilead Sciences, and ViiV Healthcare initiated the Determined, Resilient, Empowered, AIDS-free, Mentored and Safe (DREAMS) Partnership in 2015, with the goal to reduce HIV acquisition among AGYW in 14 sub-Saharan countries (including Kenya, Malawi, and Zambia) and Haiti. Using public and private delivery channels, DREAMS delivers a comprehensive package of evidence-based activities that aim to provide vulnerable AGYW with knowledge, life skills, and access to social protection interventions (e.g., educational support). DREAMS also aspires to create an enabling environment that supports AGYW's health and development by offering events and services that engage parents and the broader community.[29-32] However, there is limited evidence about how this AGYW-focused approach, which extends beyond behavioral or health sector interventions, is influencing HIV-related risk among AGYW.

To contribute to the growing body of literature around comprehensive multi-sectoral HIV prevention programs, we assess temporal shifts in factors that have been previously linked to HIV-acquisition among DREAMS participants across three country contexts. Using an age-stratified approach to illuminate risk heterogeneities, we examine change over time in proximal behavioral, biological, and experiential HIV-related risk factors to assess if participation in the DREAMS program positively influenced participants' vulnerability. This study provides initial insight on the change over time in HIV-related risk factors among cohorts of AG and YW DREAMS participants in Kenya, Malawi, and Zambia, with the aim to inform the refinement or development of comprehensive multi-sectoral HIV prevention efforts.

METHODS

Program description

For peer review only

The DREAMS program aims to empower AGYW and reduce their risk of HIV and gender-based violence by delivering a package of tailored, multi-layered activities, services, and interventions.[29-32] The DREAMS approach has been described in detail elsewhere.[29] Briefly, the core package of interventions includes four components: (1) empowering AGYW – including condom promotion, PrEP provision (only approved and provided in Kenya at the time of study), post-violence care, HIV testing services, increasing access to voluntary family planning services, and social asset building (e.g. knowledge and skills to increase self-efficacy); (2) strengthening families through parenting/caregiver programs, education subsidies and combination socioeconomic approaches; (3) mobilizing communities to address harmful community norms and practices, and school based HIV and violence prevention; and (4) reducing risk of AGYW's sexual partners by increasing access to HIV prevention and care services. DREAMS implementing partners were expected to reach ½ of all vulnerable AGYW in their study communities during the study implementation time period. [29] Country teams recruited AGYW based on a range of factors that constituted vulnerability (e.g., being out-of-school, orphaned, history of violence experience, STI experience, etc.). The program uses designated spaces within the community, where AGYW engage, often weekly, in the DREAMS activities. The safe spaces, which act as a programmatic hub and link AGYW peers and mentors, facilitated instruction on health and life skills, and referrals to youth-friendly health services and social protection interventions.

PEPFAR determined the target DREAMS program areas, with input from local officials and other stakeholders, based on their high HIV prevalence and additional factors, such as high poverty levels, population density, and/or population growth. In each site, implementing partners (IP) used systematic selection processes (e.g., visiting and screening households in their community catchment area[33] to identify, recruit, and enroll eligible AGYW beneficiaries.[34-39] In Malawi, an additional eligibility criterion was out-of-school status. In each country context, a primary package of interventions[37 39 40] was delivered, with the expectation that all DREAMS participants would receive this primary package and additional interventions, depending on individual need or vulnerability.

For peer review only

Study sites

In Kenya and Zambia, the sites for this study were purposively selected, in consultation with PEPFAR, local stakeholders, and implementing partners, to be representative of key geographic characteristics (e.g., urban/peri-urban/rural) of DREAMS program communities in each country. In Malawi, the study sites were selected from each of the two districts involved in DREAMS programming at the time. In total, the study was conducted in two sites in Kisumu County, Kenya; four sites in Malawi's Zomba and Machinga districts; and two sites in Lusaka and Ndola, Zambia.

Study population

Eligible survey participants were females aged 15–24 years residing in the study catchment area who were enrolled in the DREAMS program and intended to stay in the area for the subsequent year. Round one data was collected in 2016/2017. At round one, study participants were identified using an age-stratified random sample from the DREAMS program beneficiary rosters (Kenya [n=5997], Malawi [proportionate to the differently sized districts, n=4738]) prepared by the program IPs, whereas a census of all AGYW enrolled in DREAMS was conducted in Zambia. Potential participants were approached by study staff and invite to take part in the study. Using a conservative estimate (i.e., 50%) of baseline prevalence for the primary outcomes (e.g., HIV testing), the samples were powered at 80% to detect a minimal 10-20% percentage change. In Kenya, 474 AG and 440 YW were interviewed from October 2016 to February 2017. In Malawi, 528 AG and 1134 YW were interviewed from July 2017 to September 2017. In Zambia, 585 AG and 479 YW were interviewed from November 2016 to April 2017. Twenty respondents in Kenya, 33 in Zambia, and 3 in Malawi refused to participate due to lack of parental consent, in ability to locate the participant or limited time availability/other events that prohibited participation.

For peer review only

A second round of data was collected with round one respondents in 2018 after approximately 12-16 months of exposure to DREAMS. At round 2, 80.5% of Kenya's respondents (389 AG and 347 YW) were re-interviewed from April-June 2018, 75.5% of Malawi's respondents (371 AG and 883 YW) were re-interviewed from September-November 2018, and 79.8% of Zambia's respondents (487 AG and 398 YW) were re-interviewed from April-May 2018. Overall, loss to follow-up occurred due to extended or permanent out-migration from the study communities, as confirmed by the program team and through repeated attempts to recontact participants by the research team. Those not re-interviewed had re-located to towns outside study settings due to family, business or school reasons. Comparison of baseline characteristics and outcome values between respondents who were followed-up and those who were lost show some differences by age group or country, but do not show any systematic patterns of loss (see Supplemental Table 1). For instance, Kenyan AG who could not be re-interviewed were more likely to be out of school ($p \leq 0.05$) and orphaned ($p \leq 0.01$), but this was not the case for YW in Kenya or AG and YW in Malawi and Zambia.

Study procedures

Respondent consent (or parent/guardian consent and minor assent) was obtained before each round of data collection. Using a tablet-based survey instrument in the respondent's chosen language (Kiswahili and Luo in Kenya, Chichewa and Yao in Malawi, Bemba and Nyanja in Zambia, or English in all countries), female interviewers captured respondents' sociodemographic characteristics; program engagement and exposure; and knowledge, attitudes, and behaviors related to HIV/AIDS. Interviews were conducted in private yet convenient locations to the respondents (e.g., room in respondent's home, nearby field, or nearby community center), and out of earshot of parents, guardians, or other community members.

Measures

For peer review only

Cohorts' characteristics

The study cohorts only included respondents who provided information at both round one and two. We explored the cohorts' socio-demographic characteristics—such age (15-19 vs. 20-24) and marital (ever [currently, formerly] vs. never married), schooling (in school vs. out of school), and orphanhood (both parents alive vs. at least one parent deceased) status, and using round 2 data only, we examined their participation in social asset building activities and exposure to (i.e., being offered the service/activity by DREAMS staff) various DREAMS program components—such as HIV testing services (HTS), health services (condoms, PrEP, post-violence care, contraception, STI testing, pregnancy consultation), educational support (money for fees, uniforms, transport, or other help with schooling expenses in Kenya and Zambia; back to school support in Malawi), and economic support (cash transfer in Kenya and Zambia, food support in Malawi). We did not change the study site (location 1 vs. location 2) variable (Kenya: Nyalenda A, Kolwa East; Malawi: Zomba, Machinga; Zambia: Lusaka, Ndola).

We assessed engagement in DREAMS' primary intervention by asking respondents if they participated in the safe space groups (yes vs. no) by round 2. Additionally, to examine the level of exposure to all DREAMS activities, we asked participants about the frequency and duration (short [<1 week, 1 week] vs. long [1 month, several months]) of their program interruptions. Among those that experienced periods of non-participation, we used the median number of interruptions (3 in all countries) to classify respondents as experiencing few (<median) or many (≥median) interruptions.

Outcomes

We focused on factors that are known to facilitate or mitigate AGYW's HIV risk. HIV testing in the last 12 months and experiencing STI symptoms (genital warts, painful urination, vaginal discharge, genital ulcer) in the last 6 months were coded as binary (yes vs. no) variables. Among those who had sexually debuted, we truncated respondents' number of sexual partners in the last 12 months into a categorical variable (0, 1, 2+). We considered respondents to be consistent condom users if they were currently in a

For peer review only

sexual relationship and in the last 12 months, they always used condoms with their primary and (if applicable) secondary partner; using this same schema, we also assessed condom use at last sex. For Zambia only, the condom use variables were only asked of respondents who were currently sexually active with their primary and (if applicable) secondary partner.

Using a binary (yes vs. no) variable, we identified respondents as transactional sex engagers if they had engaged in sex with a stranger or casual partner to receive cash or kind; somewhere to stay; support or money for their children or family; drugs, food, cosmetics, clothes, a cellphone, airtime, transportation; somewhere to sleep for the night; financial support for bills or school fees; or anything else the they could not afford by themselves in the past 12 months.[41-43]

We measured intimate partner violence (IPV) among all respondents who reported having a sexual partner in the last 12 months and classified respondents as having experienced sexual IPV if they disclosed that in the last 12 months, they had sex or performed other sexual acts against their will because a current or previous boyfriend or partner used physical coercion, threats and intimation, or force. We coded respondents as having experienced physical IPV if they had been slapped or had something thrown at them; pushed or shoved; hit with a fist or another dangerous object; kicked, dragged, beaten, choked, or burnt; or threatened with a gun, knife, or other weapon by a current or previous boyfriend or partner in the last 12 months.[11]

Lastly, questions on non-partner sexual violence were asked of all AGYW. We classified respondents as having experienced non-partner sexual violence if they disclosed that in the last 12 months, a person other than their boyfriend or partner perpetrated unwanted sex by using persuasion or force—whether successful or unsuccessful; forced sex while they were under the influence of drugs or alcohol and too impaired to refuse; or forced sex by two or more men—when the respondent was either sober or under the influence of drugs or alcohol.[11]

Data analysis

For peer review only - http://bmjopen.bmj.com/site/about/guidelines.xhtml

We conducted bivariate chi-squared analyses to examine the change in outcome measures between data collection rounds. To account for intra-individual correlation, we used generalized estimating equations (with a Poisson distribution) to examine temporal changes, control for characteristics (interruptions in program participation, study site, and round one's marital, schooling, and orphanhood status), and report measures of association (Adjusted Incident Rate Ratio [95% Confidence Interval]). We ran all analyses using Stata (version 15.0).

Patients and public involvement

This study actively engaged the study community and program implementing partners in the research process. Approval for the study was sought from community leaders and representatives in each setting during study planning. Research questions and tools were vetting with program implementing partners prior to administration. Post data collection, dissemination meetings were conducted with program implementing partners and community leaders to share findings and implications for HIV prevention and other programming for adolescent girls and young women in the study communities. Short study results briefs and presentation slide decks were developed and shared with program implementing partners and other collaborators for dissemination of research results in each setting with key leaders and decision-makers.

RESULTS

Cohorts' characteristics

Table 1 presents the socio-demographic characteristics of the cohorts in Kenya, Malawi, and Zambia. Overall, the mean baseline age was between 17 (Kenya, Zambia) and 18 (Malawi) for AG and about 22 for YW (all countries). At round one, most (>50%) Kenyan and Zambian respondents were unmarried. Conversely, marriage was common among Malawian AG (62%) and YW (89%) at round one. The

For peer review only

majority of AG in Kenya and Zambia were in school (>60%) at round one, and over 50% of Zambian YW were still in school at Round 2. Across all socio-demographic characteristics, there were modest shifts over time, as only a few age groups in certain countries had significant increases in out-of-school (Kenyan AG, Zambian AG and YW) and ever-married (Malawian AG and YW) status.

Respondents had nearly universal exposure to DREAMS' primary social asset building intervention: safe space groups, and exposure to HTS was high among Kenyan AG (96.1%) and YW (91.4%) but lower among AG and YW in Malawi and Zambia. With the exception of Kenya, where approximately ≥50% of AG and YW were exposed to educational support or economic support, exposure to other health services, educational support, and economic support was low (e.g., no YW in Malawi and 17% in Zambia had received some economic support).

On average, 75% of respondents experienced some form of program interruption, with just under 2 in 3 reporting many (i.e., ≥3) interruptions that last less than a week or more than a month. In Zambia for instance, 75% of AG noted there were times when they were not able to participate in DREAMS with 29% reporting at least 5 interruptions lasting for a month or more. The main reasons for periods of non-participation included being away at school (Kenyan AG [58.9%]), sickness (Malawian AG [51.5%] and YW [47.1%]) and being away from the community (Kenyan YW [32.9%] and Zambian AG [29.0%] and YW [37.4%]).

For peer review only

Table 1: Characteristics of cohorts in Kenya, Malawi, and Zambia

Characteristics	Kenya				Malawi				Zambia			
	15-19 n=389		20-24 n=347		15-19 n=371		20-24 n=883		15-19 n=487		20-24 n=398	
	Round 1	Round 2	Round 1	Round 2	Round 1	Round 2	Round 1	Round 2	Round 1	Round 2	Round 1	Round 2
**Mean Age (SD)**	16.6 (1.3)	18.3 (1.8)	21.6 (1.4)	22.6 (1.9)	18.0 (1.2)	19.3 (1.3)	21.9 (1.4)	23.1 (1.6)	16.8 (1.4)	18.1 (1.6)	21.6 (1.3)	22.5 (1.4)
**Marital Status**					***		***					
Ever Married	4.4	6.4	44.4	49.6	62.3	74.4	88.9	93.7	2.5	3.1	8.5	11.8
Never married	95.6	93.6	55.6	50.4	37.7	25.6	11.1	6.3	97.5	96.9	91.5	88.2
**Current Schooling Status**	**								***		**	
In school	84.3	75.3	32.9	27.1	-	-	-	-	77.6	60.6	61.6	52.3
Out of school	15.7	24.7	67.1	72.9	-	-	-	-	22.4	39.4	38.4	47.7
**Orphanhood**												
Both parents alive	62.5	58.1	42.4	38.9	70.9	69.5	61.4	60.8	64.9	62.6	55.5	54.3
Lost at least one parent	37.5	41.9	57.6	61.1	29.1	30.5	38.6	39.2	35.1	37.4	44.5	45.7
**Program Participation/Exposure**												
Social Asset Building	-	91.3	-	92.2	-	96.5	-	97.3	-	90.4	-	91.5
Offered HTS	-	96.1	-	91.4	-	62.0	-	65.1	-	80.9	-	73.9
Offered other health service	-	40.2	-	44.4	-	29.1	-	30.5	-	36.2	-	34.4
Educational Support	-	66.8	-	57.6	-	1.4	-	1.1	-	46.4	-	21.9
Economic Support	-	49.6	-	57.6	-	1.0	-	0.0	-	25.1	-	16.6
**Interruptions**												
None	-	27.5	-	26.5	-	21.0	-	24.0	-	25.1	-	29.4
<3 interruptions of ≤1 week	-	6.9	-	6.3	-	9.7	-	7.1	-	5.5	-	6.5
<3 interruptions of ≥1 month	-	7.7	-	4.6	-	3.2	-	3.4	-	3.5	-	3.3
≥3 interruptions of ≤1 week	-	23.1	-	28.8	-	22.1	-	28.0	-	37.0	-	30.4
≥3 interruptions of ≥1 month	-	34.7	-	33.7	-	43.9	-	37.5	-	28.9	-	30.4

*p≤0.05; **p≤0.01; ***p≤0.001 for marital, schooling, and orphanhood status

Other health services includes condoms, PrEP (where available), post-violence care, contraception, STI testing, pregnancy consultation

Educational support includes money for fees, uniforms, transport or other help with schooling expenses (Kenya, Zambia) or back to school support (Malawi)

Economic support includes cash transfer (Kenya, Zambia) and food support (Malawi)

Program participation/exposure and interruptions measured only at Round 2

Malawi's sample only included out-of-school participants; hence, current schooling status is not reported for Malawi's cohorts

For peer review only

Kenya: Unadjusted and adjusted transitions in AG and YW's HIV risk factors

Table 2 displays the unadjusted and adjusted changes in HIV risk factors among Kenyan AG and YW. Among AG, HIV testing increased (81.5% to 96.4%, p≤0.001, aIRR=1.18 [1.02-1.37]) and reports of sexual IPV decreased (19.85% to 6.4%, p≤0.001, aIRR=0.32 [0.18-0.56). YW reported a decrease in consistent condom use (31% to 19%, p=0.004, aIRR= 0.57 [0.40-0.81]), sexual IPV (20.2% to 10.5%, p=0.001, aIRR=0.51 [0.34-0.75]), and non-partner sexual violence (30.6% to 13.5%, p≤0.001, aIRR= 0.44 [0.32-0.61]) and an increase in transactional sex (5.7% to 10.0%, p=0.044, aIRR= 1.73 [1.03-2.89]).

Table 2: Temporal shifts in behavioral, biological, and experiential HIV risk factors among AGYW in Kenya

	Aged 15-19 n=389				Aged 20-24 n=347			
	**Round 1**	**Round 2**	**p-value**	**aIRR (95% CI)**	**Round 1**	**Round 2**	**p-value**	**aIRR (95% CI)**
**HIV Service Use**								
HIV Testing in last year	81.5	96.4	**<0.001**	**1.18 (1.02-1.37)**	96.3	97.1	0.525	1.01 (0.88-1.15)
**Sexual Behavior**								
STI Symptoms	14.4	14.9	0.839	1.04 (0.72-1.50)	23.1	21.0	0.522	0.91 (0.68-1.22)
Number of sex partners			0.531	0.99 (0.81-1.23)			0.259	1.00 (0.87-1.15)
0	11.1	8.7			5.1	2.8		
1	73.0	78.6			79.8	83.8		
2+	15.9	12.7			15.1	13.4		
Consistent condom use	52.5	45.8	0.395	0.78 (0.52-1.18)	30.7	19.0	**0.004**	**0.57 (0.40-0.81)**
Condom use at last sex	74.6	64.4	0.172	0.80 (0.54-1.17)	46.3	38.0	0.071	0.77 (0.59-1.00)
Transactional sex	9.5	8.1	0.664	0.89 (0.46-1.71)	5.7	10.0	**0.044**	**1.73 (1.03-2.89)**
**Violence Perpetrated by an Intimate Partner**								
Sexual	19.8	6.4	**<0.001**	**0.32 (0.18-0.56)**	20.2	10.5	**0.001**	**0.51 (0.34-0.75)**
Physical	15.2	14.7	0.866	0.96 (0.61-1.49)	31.1	25.0	0.099	0.78 (0.60-1.02)
**Violence Perpetrated by a Non-partner**								
Sexual	21.1	19.5	0.593	0.93 (0.69-1.25)	30.5	13.5	**<0.001**	**0.44 (0.32-0.61)**

aIRR = Adjusted Incidence Rate Ratios, 95% CI = 95% Confidence Interval

aIRR adjusts for continuity of program exposure, location, and round one marital, schooling, and orphanhood status

Bolded cells indicate p≤0.05

For peer review only

Malawi: Unadjusted and adjusted changes in AG and YW's HIV risk factors

Table 3 contains the unadjusted and adjusted shifts in HIV risk factors among Malawian AG and YW. AG reported more HIV tests (85.4% to 94.5%, p≤0.001); they also reported fewer sex partners (2+ sex partners: 9.4% to 1.8%, p≤0.001) and experiences of sexual violence from partners (16.9% to 5.9%, p≤0.001, aIRR=0.35 [0.21-0.57]) and non-partners (10.0% to 4.3%, p=0.003, aIRR= 0.43 [0.25-0.75]). However, the associations for HIV testing and number of sexual partners became null in the multivariable models. Among YW, increases in HIV testing (82.5% to 91.7%, p≤0.001, aIRR= 1.11 [1.01-1.22]) were coupled with decreases in STI symptoms (31.8% to 22.1%, p≤0.001, aIRR= 0.69 [0.60-0.81]), number of sexual partners (2+ sex partners: 6.2% to 3.2%, p≤0.001), and experiences of sexual IPV (16.4% to 4.5%, p≤0.001, aIRR= 0.27 [0.19-0.39]), physical IPV (17.2% to 11.8%, p=0.001, IRR= 0.68 [0.55-0.85]), and non-partner sexual violence (8.5% to 1.8%, p≤0.001, aIRR= 0.21 [0.13-0.36]). In the multivariable models, the association for number of sexual partners became null.

Table 3: Longitudinal changes in behavioral, biological, and experiential HIV risk factors among Malawian AGYW

	**Aged 15-19 n=371**				**Aged 20-24 n=883**			
	**Round 1**	**Round 2**	**p-value**	**aIRR (95% CI)**	**Round 1**	**Round 2**	**p-value**	**aIRR (95% CI)**
**HIV Service Use**								
HIV Testing in last year	85.4	94.5	**<0.001**	1.11 (0.95-1.29)	82.5	91.7	**<0.001**	**1.11 (1.01-1.22)**
**Sexual Behavior**								
STI Symptoms	25.6	22.4	0.302	0.87 (0.67-1.14)	31.8	22.1	**<0.001**	**0.69 (0.60-0.81)**
Number of sex partners			**<0.001**	1.01 (0.86-1.17)			**<0.001**	1.08 (0.98-1.18)
0	20.4	12.3			23.5	14.3		
1	70.2	86.0			70.3	82.5		
2+	9.4	1.8			6.2	3.2		
Consistent condom use	11.8	6.8	0.056	0.60 (0.32-1.11)	5.0	5.4	0.743	0.92 (0.59-1.43)
Condom use at last sex	20.2	20.3	0.967	1.01 (0.68-1.51)	14.0	15.7	0.395	1.05 (0.81-1.37)
Transactional sex	2.4	3.5	0.412	1.39 (0.63-3.11)	3.9	3.0	0.281	0.76 (0.47-1.23)
**Violence Perpetrated by an Intimate Partner**								
Sexual	16.9	5.9	**<0.001**	**0.35 (0.21-0.57)**	16.4	4.5	**<0.001**	**0.27 (0.19-0.39)**
Physical	13.9	11.2	0.29	0.80 (0.55-1.16)	17.2	11.8	**0.001**	**0.68 (0.55-0.85)**
**Violence Perpetrated by a Non-partner**								
Sexual	10.0	4.3	**0.003**	**0.43 (0.25-0.75)**	8.5	1.8	**<0.001**	**0.21 (0.13-0.36)**

aIRR = Adjusted Incidence Rate Ratios, 95% CI = 95% Confidence Interval

aIRR adjusts for continuity of program exposure, location, and round one marital and orphanhood status. We did not control for schooling because out-of-school status was a participation requirement in Malawi.

For peer review only - http://bmjopen.bmj.com/site/about/guidelines.xhtml

Bolded cells indicate $p \leq 0.05$

For peer review only

Zambia: Unadjusted and adjusted shifts in AG and YW's HIV risk factors

Table 4 presents the unadjusted and adjusted transitions in HIV risk factors among Zambian AG and YW. For AG, HIV testing increased over time (47.4% to 74.1%, p≤0.001, aIRR=1.56 [1.34-1.82]). YW reported more HIV testing (67.6% to 76.1%, p=0.007) and transactional sex (2.4% to 5.8%, p=0.049, aIRR= 2.62 [1.02-6.75]) and fewer experiences of non-partner sexual violence (23.4% to 14.1%, p=0.001, aIRR= 0.60 [0.44-0.82]), but the increase in HIV testing became null in the multivariable models.

Table 4: Changes over time in behavioral, biological, and experiential HIV risk factors among AGYW in Zambia

	Aged 15-19 n=487				Aged 20-24 n=398			
	**Round 1**	**Round 2**	**p-value**	**aIRR (95% CI)**	**Round 1**	**Round 2**	**p-value**	**aIRR (95% CI)**
**HIV Service Use**								
HIV Testing in last year	47.4	74.1	**<0.001**	**1.56 (1.34-1.82)**	67.6	76.1	**0.007**	1.13 (0.99-1.28)
**Sexual Behavior**								
STI Symptoms	12.7	11.5	0.556	0.90 (0.65-1.25)	15.8	15.8	1.000	1.00 (0.75-1.34)
Number of sex partners			0.111	0.91 (0.73-1.16)			0.450	0.94 (0.81-1.10)
0	7.6	16.1			15.0	17.0		
1	85.9	76.5			72.1	73.3		
2+	6.6	7.4			13.0	9.8		
Consistent condom use	42.5	38.8	0.706	0.81 (0.44-1.51)	42.3	33.3	0.167	0.74 (0.48-1.13)
Condom use at last sex	60.0	55.2	0.629	0.85 (0.51-1.44)	52.6	45.5	0.287	0.84 (0.58-1.19)
Transactional sex	0.9	3.7	0.163	3.57 (0.42-30.31)	2.4	5.8	**0.049**	**2.62 (1.02-6.75)**
**Violence Perpetrated by an Intimate Partner**								
Sexual	17.6	17.9	0.923	1.03 (0.72-1.46)	18.4	14.4	0.144	0.78 (0.55-1.11)
Physical	23.3	23.9	0.859	1.03 (0.77-1.36)	15.6	12.1	0.172	0.75 (0.52-1.09)
**Violence Perpetrated by a Non-partner**								
Sexual	12.5	10.9	0.425	0.87 (0.61-1.23)	23.4	14.1	**0.001**	**0.60 (0.44-0.82)**

aIRR = Adjusted Incidence Rate Ratios, 95% CI = 95% Confidence Interval

aIRR adjusts for continuity of program exposure, location, and round one marital, schooling, and orphanhood status

Bolded cells indicate p≤0.05

For peer review only

DISCUSSION

We present one of the first multi-country analyses examining temporal changes in behaviors and experiences associated with HIV acquisition among cohorts of AG and YW enrolled in DREAMS in Kenya, Malawi, and Zambia. Our analysis uses prospective data and presents stratified analyses by age group. Our findings illuminate heterogeneous shifts in HIV related risk factors between AG and YW, and across countries. Over time, AG who were part of DREAMS report increases in HIV testing (Kenya and Zambia), initiated sexual activity (Kenya and Zambia) and decreases in sexual IPV (Kenya and Malawi) and non-partner sexual violence (Malawi). HIV testing increased and experience of STIs decreased among YW in Malawi. Additionally, YW report increases in transactional sex (Kenya and Zambia) and decreases in consistent condom use (Kenya). YW engaged in DREAMS also report lower sexual IPV (Kenya and Malawi), lower physical IPV (Malawi), and non-partner sexual violence (Kenya, Malawi, and Zambia). Some emerging research in Uganda report that DREAMS programming contributed to the reduction of sexual risk behaviors; though, the effects differed by age group.[44] While the DREAMS programming models included considerations for participant age (e.g. providing education support to AG, and livelihoods support to YW), our findings indicate that additional tailoring of comprehensive HIV prevention packages may be needed to address the risk heterogeneity of AG and YW across different contexts.[43]

Increases in HIV testing rates among Kenyan and Zambian AG and Malawian YW are promising as HIV testing is the first step in the HIV care cascade and remains a key prevention strategy. Our data show that close to 75% (or greater) of AG and YW self-reported testing for HIV in the last year. In comparison, recent national averages of self-reported HIV testing are much lower (Kenya: 35% of AG, 64% of YW; Malawi: 28% of AG, 52% of YW; Zambia: 29% of AG, 53% of YW).[3 4 45] These results likely point to PEPFAR, local national HIV programs and policies, and DREAMS' focus on expanded access to HIV testing services to AGYW in the DREAMS study communities.[29] Though only observed within one group, it is also encouraging to see that Malawian YW reported fewer STI symptoms over time, as STIs

For peer review only

increase AGs' and YW's biological vulnerability to HIV.[46 47] Within DREAMS, establishing, providing, and strengthening youth-friendly health services was a key part of the intervention package. AG and YW were primarily linked to these services through discussions and referrals provided in the safe space groups. This community-based platform may have been particularly helpful in bringing services to marginalized AGYW, who would otherwise be hard to reach with HIV and reproductive health programming.[48]

Decreases in experience of sexual IPV over time are also promising. Previous research supports the direct relationship between HIV acquisition and sexual IPV, primarily because it facilitates other risk behaviors and mechanisms (e.g., decrease in self-efficacy and increases in multiple partnerships, transactional sex, substance abuse, negative mental health outcomes, and biological vulnerability due to trauma).[10 11 49 50] Compared with data from nationally-representative surveys among ever-married women aged 15-49, the baseline prevalence for sexual IPV among AGYW in our cohorts are high.[45 51] The DREAMS programs were conducted in high-risk communities with the most-at-risk AGYW, which may account for the higher experiences of sexual violence than the national average. Over time, we find that AG and YW in Kenya and Malawi reported significantly fewer incidents of sexual IPV over time. Since it is also perpetrated by a trusted individual, physical IPV has similar mediating pathways.[10 52] In, almost, all countries and age groups, physical IPV reduced, yet the reduction was only significant among Malawian YW and could be because most Malawian YW were ever married at round one and, therefore, had the greatest opportunity for positive change; they also had the highest mean age, which could have translated into greater agency and relationship power. Additional evidence is needed to support or refute these hypotheses. Zambian AG and YW reported no significant shifts related to either IPV outcome; this warrants further investigation as to how IPV is perceived among Zambian AGYW and how programs are attempting to address it.

Sexual violence perpetrated by non-partners (assault and rape) is understudied; in our analysis, the baseline prevalence of non-partner sexual violence ranged from 10.0% to 21.1% among AG and 8.5% to 30.5% among YW. These totals are likely underestimated due to the topic's sensitive nature, which can

For peer review only

trigger recollections of sadness and trauma; regardless, compared with other non-partner sexual violence metrics (9.1% prevalence among Johannesburg AG,[13] 11.9% [8.5%-15.3%] lifetime prevalence among African women[52]), these baseline totals are high. Other studies have found that surviving non-partner sexual violence can lead to a myriad of behaviors or outcomes that heighten HIV risk (e.g., substance abuse, poorer sexual and reproductive health and mental health outcomes, and increased experience of STIs).[11 13 52] In our study, it is reassuring that we observed appreciable drops in non-partner sexual violence among DREAMS participants across all age groups and countries, which hopefully signifies community-wide shifts in accountability around violence and safer spaces for AGYW. DREAMS' multi-sectoral approach could explain some of the notable decrease in intimate and non-partner violence. Gender-inequitable social norms and patriarchal power structures, often, create environments where men are emboldened to perpetrate violence, both against their intimate and strangers/casual partners. To disrupt these norms, DREAMS programming made a concerted effort to create an enabling environment for AGYW by challenging existing power dynamics and addressing gender-based violence—both within the safe space groups and the broader programming community.[29-32] However, since all girls were exposed to anti-violence programming and our study does not include any data from men or other community members, this study cannot disentangle its effects from other activities. To strengthen our understanding, future research should consider collecting attitudes and behaviors related to non-partner violence from various ecological strata, and further examination is required to unpack the specific programming components and pathways that may have contributed to these significant changes.

We do not find substantial shifts in other preventive behaviors (i.e., increase in condom use, decrease in multiple partnerships). On average, consistent condom use and condom use at last sex decreased between survey rounds, with the greatest decrease occurring among Kenyan YW. While previous research shows that proper condom use is protective against HIV acquisition,[53-55] condom uptake among adolescent and youth populations is consistently suboptimal,[53 56-59] a pattern that is reinforced by our findings. A post-hoc trend decomposition analysis suggests that condom use behaviors may have shifted for YW as they

For peer review only

entered martial partnerships (results not shown). Previous literature suggests that low condom use among AG and YW could be a product of failed negotiations fueled by low self-efficacy, relationship power, and gender-inequitable norms and practices.[6 18 43 60 61] Alternatively, condom use could have decreased due to changes in contraceptive use—such as moving from condoms to hormonal contraceptives—or desires to have children with their spouse.[62-64] Multiple factors and motivations may be contributing to shifts in condom use among AG and YW, especially those in recent unions. For HIV prevention programming, it will be critical to consider approaches to ensure that AGYW and their partners employ strategies (e.g., couple HIV testing and counseling, safer conception practices) to mitigate HIV risk as they transition in and out of relationships and have children.

Among Kenyan and Zambian YW, we found significantly higher reports of transactional sex over time. Previous research shows that transactional sex can increase women's HIV risk.[19 20 41 65 66] To avert poverty, homelessness, and food insecurity, some may turn to transactional sex to survive or to obtain desired commodities.[17 18 67] AGYW's economic disenfranchisement is characterized by a constellation of factors—including gendered discrimination and lack of social and business capital[68]—that make it difficult to AGYW to access the economic resources or earn a living wage. While DREAMS provided some economic activities (e.g., financial literacy and education) and strengthened access to some economic resources (e.g., access to school bursary programs or savings accounts), it is possible that broader partnerships or deeper investments are needed to make gainful employment/entrepreneurship possible for AGYW. Additionally, it is possible that the implementation of the socio-economic interventions within DREAMS may have faltered. Research in South Africa on the DREAMS Partnership found that rollout of program components that required multi-sectoral collaborations (especially those beyond the health sector) were complex and challenging in a community-based setting.[69] Other structural intervention programs have reported reductions in economically-driven sex, risky sexual practices (e.g., condomless sex, multiple sex partners).[70-73] These positive shifts show that intersectional approaches can

For peer review only

lead to meaningful change and should continue be considered by future HIV prevention programs for AGYW.[74-76]

While we found some promising results, this study is not without limitations. Across the three countries, 17-25% of the respondents could not be re-interviewed at round two; however, this level of attrition is consistent with other community-based safe space programs.[77] While our original sample size calculations were estimated anticipating this loss to follow-up and the lost-to-follow-up analysis (Supplemental Table 1) shows similar distributions for most baseline characteristics and outcomes, this attrition could have introduced selection bias. To avoid this in future studies, robust retention techniques should be employed. In Zambia, we could only assess condom use among a specific subset of sexually active respondents, thus our findings should not be generalized to all sexually active AGYW. All the data were self-reported, which is prone to social desirability and recall bias. An additional challenge was the lack of a control group; since the DREAMS program was implemented over time, it continued to enroll AGYW from nearby communities and catchment areas, making it hard to find an appropriate counterfactual group. It also means our findings could be prone to confounding by secular trends and the aging of the cohorts in the study contexts. Subsequent analyses using modeling techniques using age as the timescale to create a control group could further help to reduce bias.

CONCLUSION

Recent large-scale HIV prevention efforts are currently underway to reduce the disproportionate burden of HIV among AGYW in sub-Saharan Africa. Our longitudinal analysis assessed temporal change in key behaviors and experiences associated with HIV acquisition among Kenyan, Malawian, and Zambian AGYW cohorts nested within DREAMS, a multi-sectoral HIV prevention program. These analyses found a positive effect on some HIV risk factors, such as HIV testing, non-partner sexual violence, and physical and sexual intimate partner violence, but variable improvements in sexual risk behaviors. Our findings

For peer review only

426 contribute to the growing body of evidence that is assessing the impact of community-based multi-

427 sectoral HIV prevention programming for AGYW.

For peer review only

For peer review only - http://bmjopen.bmj.com/site/about/guidelines.xhtml

Acknowledgements

The authors would like to acknowledge the implementing partners in Kenya (Aphia Plus/PATH, I Choose Life, Make Me Smile), Malawi (OneCommunity), and Zambia (PACT, CMMB, Marie Stopes Zambia), and our research team coordinators and data collectors for their support during the data collection. We would like to acknowledge support from Population Council colleagues who provided support in the preparation of this manuscript, and our deepest gratitude goes to the study participants. This work would not have been possible without the active support of the PEPFAR DREAMS teams in Kenya, Malawi, and Zambia.

Contributors

SM, NP, and JP conceptualized the study. SM, NP, CJH, SKP, and BM devised and implemented the analysis plan. EC, VM, WC, JO, and MM supported the study design and led the data collection. SM and CJH led the manuscript writing. All authors reviewed and revised the manuscript.

Funding

Funding support for the Kenya and Zambia studies was provided by the Bill & Melinda Gates Foundation (OPP1136778, DREAMS Implementation Science Research). Funding for the Malawi study was provided by the generous support of the American people through the United States President's Emergency Plan for AIDS Relief (PEPFAR) and the United States Agency for International Development (USAID) under Project SOAR (Cooperative Agreement AID–OAA–A–14–00060). The contents of this manuscript are the sole responsibility of the authors and do not necessarily reflect the views of PEPFAR, USAID, or the United States Government.

Competing interests

For peer review only

NP works full-time for ViiV Healthcare. ViiV Healthcare did not fund the study. The contents of the manuscript do not reflect the views of ViiV Healthcare. SM, CJH, SKP, JO, EC, VM, WC, MM, BM, and JP declare no conflicts of interest.

Patient consent for publication

Not required

Provenance and peer review

Not commissioned, externally peer reviewed

Data availability statement

All the underlying data relevant to the study are fully available on Dataverse.

Ethics statement

At each survey round, we collected written informed consent from adult participants (those above the age of consent in each country) and emancipated minors (when applicable). If the participant was considered a minor, we obtained parental consent and participant assent. The protocols for these studies were reviewed and approved by Population Council's Institutional Review Board (protocol # 744 and 745); Kenya's Kenyatta National Hospital/University of Nairobi Ethics (P384/05/2016) and Research Committee and National Commission for Science, Technology, and Innovation; Malawi's College of Medicine Research and Ethics Committee at the University of Malawi (PP.01/17/2095); and Zambia's ERES Converge Institutional Review Board (2016- May-016) and National Health Research Authority. Participants were compensated 300 Kenyan Shilling (~USD $3), 1500 Malawian Kwacha (~ USD $2), or

For peer review only

50 Zambian Kwacha (~USD $5) for their time, per local ethical guidelines. Participation in this study did not influence access to or participation in any of the DREAMS interventions.

For peer review only

REFERENCES

1. UNAIDS. Women and HIV: A Spotlight on Adolescent Girls and Young Women: UNAIDS, 2019.

2. UNAIDS. People Living With HIV: HIV Prevalence 2018 [Available from: https://aidsinfo.unaids.org/ accessed June 12 2020.

3. Ministry of Health Zambia. Zambia Population-based HIV Impact Assessment (ZAMPHIA) 2016: Final Report: Ministry of Health, 2019.

4. Ministry of Health Malawi. Malawi Population-Based HIV Impact Assessment (MPHIA) 2015-2016: Final Report: Ministry of Health, 2018.

5. Ministry of Health Kenya: National AIDS Control Council. Kenya HIV Estimates: Report 2018, 2018.

6. UNAIDS. HIV prevention among adolescent girls and young women. Geneva, Switzerland, 2016.

7. Santelli JS, Edelstein ZR, Mathur S, et al. Behavioral, biological, and demographic risk and protective factors for new HIV infections among youth, Rakai, Uganda. *J Acquir Immune Defic Syndr* 2013;63(3):393.

8. Mabaso M, Sokhela Z, Mohlabane N, et al. Determinants of HIV infection among adolescent girls and young women aged 15–24 years in South Africa: a 2012 population-based national household survey. *BMC Public Health* 2018;18(1):183.

9. Pettifor AE, Rees HV, Kleinschmidt I, et al. Young people's sexual health in South Africa: HIV prevalence and sexual behaviors from a nationally representative household survey. *AIDS* 2005;19(14):1525-34.

10. Jewkes RK, Dunkle K, Nduna M, et al. Intimate partner violence, relationship power inequity, and incidence of HIV infection in young women in South Africa: a cohort study. *Lancet* 2010;376(9734):41-48.

11. Mathur S, Okal J, Musheke M, et al. High rates of sexual violence by both intimate and non-intimate partners experienced by adolescent girls and young women in Kenya and Zambia: Findings around violence and other negative health outcomes. *PLoS One* 2018;13(9)

For peer review only

495 12. Li Y, Marshall CM, Rees HC, et al. Intimate partner violence and HIV infection among women: a
496 systematic review and meta-analysis. *J Int AIDS Soc* 2014;17(1):18845.
497 13. Decker MR, Peitzmeier S, Olumide A, et al. Prevalence and health impact of intimate partner violence
498 and non-partner sexual violence among female adolescents aged 15–19 years in vulnerable urban
499 environments: a multi-country study. *J Adolesc Health* 2014;55(6):S58-S67.
500 14. Balkus JE, Brown E, Palanee T, et al. An empiric HIV risk scoring tool to predict HIV-1 acquisition
501 in African women. *J Acquir Immune Defic Syndr* 2016;72(3):333.
502 15. Mathur S, Wei Y, Zhong X, et al. Partner characteristics associated with HIV acquisition among
503 youth in Rakai, Uganda. *J Acquir Immune Defic Syndr* 2015;69(1):75.
504 16. Harrison A, Cleland J, Frohlich J. Young people's sexual partnerships in KwaZulu-Natal, South
505 Africa: patterns, contextual influences, and HIV risk. *Stud Fam Plann* 2008;39(4):295-308.
506 17. Wamoyi J, Buller AM, Nyato D, et al. "Eat and you will be eaten": a qualitative study exploring costs
507 and benefits of age-disparate sexual relationships in Tanzania and Uganda: implications for girls'
508 sexual and reproductive health interventions. *Reprod Health* 2018;15(1):207.
509 18. Chapman J, do Nascimento N, Mandal M. Role of Male Sex Partners in HIV Risk of Adolescent Girls
510 and Young Women in Mozambique. *Glob Health Sci Pract* 2019;7(3):435-46.
511 19. Jewkes R, Dunkle K, Nduna M, et al. Transactional sex and HIV incidence in a cohort of young
512 women in the stepping stones trial. *J AIDS Clin Res* 2012;3(5)
513 20. Kilburn K, Ranganathan M, Stoner MC, et al. Transactional sex and incident HIV infection in a
514 cohort of young women from rural South Africa. *AIDS* 2018;32(12):1669.
515 21. Price JT, Rosenberg NE, Vansia D, et al. Predictors of HIV, HIV risk perception, and HIV worry
516 among adolescent girls and young women in Lilongwe, Malawi. *J Acquir Immune Defic Syndr*
517 2018;77(1):53.
518 22. Birdthistle IJ, Floyd S, Machingura A, et al. From affected to infected? Orphanhood and HIV risk
519 among female adolescents in urban Zimbabwe. *AIDS* 2008;22(6):759-66.

For peer review only

23. Karim QA, Kharsany AB, Leask K, et al. Prevalence of HIV, HSV-2 and pregnancy among high school students in rural KwaZulu-Natal, South Africa: a bio-behavioural cross-sectional survey. *Sex Transm Infect* 2014;90(8):620-26.

24. Nyirenda M, McGrath N, Newell M-L. Gender differentials in the impact of parental death: adolescent's sexual behaviour and risk of HIV infection in rural South Africa. *Vulnerable Child Youth Stud* 2010;5(3):284-96.

25. Operario D, Pettifor A, Cluver L, et al. Prevalence of parental death among young people in South Africa and risk for HIV infection. *J Acquir Immune Defic Syndr* 2007;44(1):93-98.

26. Nyato D, Materu J, Kuringe E, et al. Prevalence and correlates of partner violence among adolescent girls and young women: Evidence from baseline data of a cluster randomised trial in Tanzania. *PLoS One* 2019;14(10)

27. Hargreaves JR, Bonell CP, Boler T, et al. Systematic review exploring time trends in the association between educational attainment and risk of HIV infection in sub-Saharan Africa. *AIDS* 2008;22(3):403-14.

28. Toska E, Gittings L, Hodes R, et al. Resourcing resilience: social protection for HIV prevention amongst children and adolescents in Eastern and Southern Africa. *Afr J AIDS Res* 2016;15(2):123-40.

29. Saul J, Bachman G, Allen S, et al. The DREAMS core package of interventions: a comprehensive approach to preventing HIV among adolescent girls and young women. *PLoS One* 2018;13(12):e0208167.

30. Gourlay A, Birdthistle I, Mthiyane NT, et al. Awareness and uptake of layered HIV prevention programming for young women: analysis of population-based surveys in three DREAMS settings in Kenya and South Africa. *BMC Public Health* 2019;19(1):1417.

31. Office of the U.S. Global AIDS Coordinator and Health Diplomacy. Preventing HIV in adolescent girls and young women: guidance for PEPFAR country teams on the DREAMS partnership: President’s Emergency Plan for AIDS Relief, 2016.

For peer review only

546 32. Office of the U.S. Global AIDS Coordinator and Health Diplomacy. DREAMS core package of interventions summary: President's Emergency Plan for AIDS Relief, 2017.

548 33. The Girl Roster: A Practical Tool for Strengthening Girl-Centered Programming. New York, NY: The Population Council, 2019.

550 34. The United States Department of State. Kenya: DREAMS Overview (FY 2016-2021), 2020.

551 35. The United States Department of State. Malawi: DREAMS Overview (FY 2016-2021), 2020.

552 36. The United States Department of State. Zambia: DREAMS Overview (FY 2016-2021), 2020.

553 37. PATH. The Power of Dreams: HIV Prevention for AGYW in Western Kenya, 2019.

554 38. USAID. USAID/Zambia Health Office: Zambia Community HIV Prevention Project (Z-CHPP) 2020 [Available from: https://www.usaid.gov/zambia/fact-sheets/usaidzambia-health-office-zambia-community-hiv-prevention-project-z.

557 39. One Community. What We Do: DREAMS 2016 [Available from: http://www.onecommunitymw.org/index.php/what-we-do/dreams.

559 40. Pact. DREAMS and OVC, 2020.

560 41. Dunkle KL, Jewkes RK, Brown HC, et al. Transactional sex among women in Soweto, South Africa: prevalence, risk factors and association with HIV infection. *Soc Sci Med* 2004;59(8):1581-92.

562 42. Jewkes R, Nduna M, Levin J, et al. A cluster randomized-controlled trial to determine the effectiveness of Stepping Stones in preventing HIV infections and promoting safer sexual behaviour amongst youth in the rural Eastern Cape, South Africa: trial design, methods and baseline findings. *Trop Med Int Health* 2006;11(1):3-16.

566 43. Mathur S, Pilgrim N, Patel SK, et al. HIV vulnerability among adolescent girls and young women: a multi-country latent class analysis approach. *Int J Public Health* 2020:1-13.

568 44. Hadijja Nakawooya, Victor Ssempijja, Dorean Nabukalu, et al. PEPFAR DREAMS Intervention Among Adolescent Girls and Young Women in Rakai, Uganda 2020 [Available from: https://www.croiconference.org/abstract/pepfar-dreams-intervention-among-adolescent-girls-and-young-women-in-rakai-uganda/.

For peer review only

45. Kenya National Bureau of Statistics, Ministry of Health/Kenya, National AIDS Control Council/Kenya, et al. Kenya Demographic and Health Survey 2014. Rockville, MD, USA, 2015.

46. Kalichman SC, Pellowski J, Turner C. Prevalence of sexually transmitted co-infections in people living with HIV/AIDS: systematic review with implications for using HIV treatments for prevention. *Sex Transm Infect* 2011;87(3):183-90.

47. Mathur S, Mullinax M, Santelli JS. Prevention of sexually transmitted infections among adolescents. In: Cherry A., Baltag V., M. D, eds. International handbook on adolescent health and development: Springer 2017:183-205.

48. Denno DM, Chandra-Mouli V, Osman M. Reaching youth with out-of-facility HIV and reproductive health services: a systematic review. *J Adolesc Health* 2012;51(2):106-21.

49. Jewkes R, Gibbs A, Jama-Shai N, et al. Stepping Stones and Creating Futures intervention: shortened interrupted time series evaluation of a behavioural and structural health promotion and violence prevention intervention for young people in informal settlements in Durban, South Africa. *BMC Public Health* 2014;14(1):1325.

50. Stockman JK, Lucea MB, Campbell JC. Forced sexual initiation, sexual intimate partner violence and HIV risk in women: a global review of the literature. *AIDS Behav* 2013;17(3):832-47.

51. National Statistical Office/Malawi, ICF. Malawi Demographic and Health Survey 2015-16. Zomba, Malawi: National Statistical Office and ICF, 2017.

52. García-Moreno C, Pallitto C, Devries K, et al. Global and regional estimates of violence against women: prevalence and health effects of intimate partner violence and non-partner sexual violence: World Health Organization, 2013.

53. Thurman T, Kidman R, Carton T, et al. Psychological and behavioral interventions to reduce HIV risk: evidence from a randomized control trial among orphaned and vulnerable adolescents in South Africa. *AIDS Care* 2016;28(sup1):8-15.

54. Hallman K, Mubayiwa R, Madya S, et al. Program versus Comparison Endline Survey of the Mabinti Tushike Hatamu! (Girls Lets Be Leaders!) Programme in Tanzania: Population Council, The

For peer review only

CSR Group Africa Limited, Restless Development Tanzania, UNICEF Tanzania, Tanzania AIDS Commission, 2016.

55. Davis KR, Weller SC. The effectiveness of condoms in reducing heterosexual transmission of HIV. *Fam Plann Perspect* 1999:272-79.

56. Erulkar AS, Ettyang LI, Onoka C, et al. Behavior change evaluation of a culturally consistent reproductive health program for young Kenyans. *Int Fam Plan Perspect* 2004:58-67.

57. Erulkar A, Chong E. Evaluation of a savings & micro-credit program for vulnerable young women in Nairobi. Nairobi, Kenya, 2005.

58. Jewkes R, Nduna M, Levin J, et al. Impact of stepping stones on incidence of HIV and HSV-2 and sexual behaviour in rural South Africa: cluster randomised controlled trial. *BMJ* 2008;337:a506.

59. George G, Maughan-Brown B, Beckett S, et al. Coital frequency and condom use in age-disparate partnerships involving women aged 15 to 24: evidence from a cross-sectional study in KwaZulu-Natal, South Africa. *BMJ Open* 2019;9(3):e024362.

60. Pulerwitz J, Mathur S, Woznica D. How empowered are girls/young women in their sexual relationships? Relationship power, HIV risk, and partner violence in Kenya. *PLoS One* 2018;13(7)

61. Kalichman SC, Simbayi LC, Cain D, et al. Sexual assault, sexual risks and gender attitudes in a community sample of South African men. *AIDS Care* 2007;19(1):20-27.

62. Kosugi H, Shibanuma A, Kiriya J, et al. Consistent condom use among highly effective contraceptive users in an HIV-endemic area in rural Kenya. *PLoS One* 2019;14(5):e0216208.

63. Brahmbhatt H, Santelli J, Kaagayi J, et al. Pregnancy Incidence and Fertility Desires Among Couples by HIV Status in Rakai, Uganda. *JAIDS Journal of Acquired Immune Deficiency Syndromes* 2019;80(5):494-502.

64. Osei IF, Mayhew SH, Biekro L, et al. Fertility decisions and contraceptive use at different stages of relationships: windows of risk among men and women in accra. *International perspectives on sexual and reproductive health* 2014;40(3):135-43.

For peer review only

For peer review only - http://bmjopen.bmj.com/site/about/guidelines.xhtml

65. Rositch A, Cherutich P, Brentlinger P, et al. HIV infection and sexual partnerships and behaviour among adolescent girls in Nairobi, Kenya. *Int J STD AIDS* 2012;23(7):468-74.

66. Ranganathan M, Heise L, Pettifor A, et al. Transactional sex among young women in rural South Africa: prevalence, mediators and association with HIV infection. *J Int AIDS Soc* 2016;19(1):20749.

67. Gichane MW, Moracco KE, Pettifor AE, et al. Socioeconomic Predictors of Transactional Sex in a Cohort of Adolescent Girls and Young Women in Malawi: A Longitudinal Analysis. *AIDS Behav* 2020

68. Stoebenau K, Wamoyi J, Fielding-Miller R, et al. Transactional sex and HIV risk, 2017.

69. Chimbindi N, Birdthistle I, Floyd S, et al. Directed and target focused multi-sectoral adolescent HIV prevention: insights from implementation of the 'DREAMS Partnership'in rural South Africa. *J Int AIDS Soc* 2020;23:e25575.

70. Cluver LD, Orkin MF, Yakubovich AR, et al. Combination social protection for reducing HIV-risk behavior amongst adolescents in South Africa. *J Acquir Immune Defic Syndr* 2016;72(1):96.

71. Dunbar MS, Dufour M-SK, Lambdin B, et al. The SHAZ! project: results from a pilot randomized trial of a structural intervention to prevent HIV among adolescent women in Zimbabwe. *PLoS One* 2014;9(11):e113621.

72. Cluver L, Toska E, Orkin F, et al. Achieving equity in HIV-treatment outcomes: can social protection improve adolescent ART-adherence in South Africa? *AIDS Care* 2016;28(sup2):73-82.

73. Bandiera O, Buehren N, Burgess R, et al. Women's empowerment in action: evidence from a randomized control trial in Africa: World Bank, 2018.

74. STRIVE Research Consortium. Why STRIVE?: London School of Hygiene & Tropical Medicine, 2016.

75. STRIVE Research Consortium. About STRIVE 2020 [Available from: http://strive.lshtm.ac.uk/about.

76. Delany-Moretlwe S, Hargreaves J, Stangly A, et al. Biomedical and structural prevention: STRIVE in the era of 'cascades', 2018.

For peer review only

77. Temin M, Heck CJ. Close to Home: Evidence on the Impact of Community-Based Girl Groups. *Glob Health Sci Pract* 2020;8(2):300-24.

For peer review only

Supplemental Table 1. Loss-to-follow-up analysis of adolescent girls and young women interviewed at Round 1 in Kenya, Malawi, and Zambia

Characteristics/Outcomes	Kenya				Malawi				Zambia			
	15-19		20-24		15-19		20-24		15-19		20-24	
	Followed n=389	Lost n=85	Followed n=347	Lost n=93	Followed n=371	Lost n=159	Followed n=883	Lost n=250	Followed n=487	Lost n=98	Followed n=398	Lost n=81
**Age**												
Mean (SD)	16.6 (1.3)	16.7 (1.3)	21.6 (1.4)	21.4 (1.4)	18.0 (1.2)	17.9 (1.2)	21.9 (1.4)	22.0 (1.5)	16.8 (1.4)	16.7 (1.4)	21.6 (1.3)	21.5 (1.3)
**Marital Status**												
Ever Married	4.4	4.7	44.4	35.5	62.3	55.4	88.9	86.8	2.5	2.0	8.5	6.2
Never married	95.6	95.3	55.6	64.5	37.7	44.6	11.1	13.2	97.5	98.0	91.5	93.8
**Current Schooling Status**	*											
In school	84.3	75.3	32.9	28.0	-	-	-	-	77.6	82.7	61.6	60.5
Out of school	15.7	24.7	67.1	72.0	-	-	-	-	22.4	17.3	38.4	39.5
**Orphanhood**	**											
Both parents alive	62.5	45.9	42.4	37.6	70.9	74.8	61.4	61.4	64.9	61.2	55.5	51.9
Lost at least one parent	37.5	54.1	57.6	62.4	29.1	25.2	38.6	38.8	35.1	38.8	44.5	48.1
**Site**					**		*		**			
Location 1	49.6	56.5	47.8	55.9	33.7	19.5	31.7	23.2	37.0	55.1	64.6	71.6
Location 2	50.4	43.5	52.2	44.1	66.3	80.5	68.3	76.8	63.0	44.9	35.4	28.4
**HIV Service Use**												
HIV test in last 12 months	**											
No	18.5	5.9	3.7	0.0	14.6	14.1	17.5	19.4	52.6	53.1	32.4	33.3
Yes	81.5	94.1	96.3	100.0	85.4	85.9	82.5	80.6	47.4	46.9	67.6	66.7
**Sexual Behavior**												

For peer review only

Experience of Sexually transmitted infection symptoms									*			
No	85.6	82.3	76.9	78.5	74.4	69.2	68.2	65.6	87.3	78.6	84.2	82.7
Yes	14.4	17.7	23.1	21.5	25.6	30.8	31.8	34.4	12.7	21.4	15.8	17.3
Number of sex partners									***			
	n=126	n=35	n=317	n=87	n=329	n=130	n=876	n=245	n=106	n=32	n=254	n=46
0	11.1	8.6	5.1	4.6	20.4	21.5	23.5	24.5	7.6	6.3	15.0	8.7
1	73.0	80.0	79.8	80.5	70.2	67.7	70.3	72.6	85.8	62.5	72.0	73.9
2+	15.9	11.4	15.1	14.9	9.4	10.8	6.2	2.9	6.6	31.3	13.0	17.4
Consistent condom use												
	n=59	n=19	n=218	n=59	n=238	n=92	n=684	n=184	n=40	n=8	n=97	n=16
No	47.5	47.4	69.3	66.1	88.2	91.3	95.0	94.0	57.5	50.0	57.7	56.3
Yes	52.5	52.6	30.7	33.9	11.8	8.7	5.0	6.0	42.5	50.0	42.3	43.8
Condom use at last sex												
	n=59	n=19	n=218	n=59	n=238	n=92	n=684	n=184	n=40	n=8	n=97	n=16
No	25.4	26.3	53.7	44.1	79.8	76.1	86.0	84.2	40.0	25.0	47.4	43.8
Yes	74.6	73.7	46.3	55.9	20.2	23.9	14.0	15.8	60.0	75.0	52.6	56.3
Transactional sex									*			
	n=126	n=35	n=317	n=87	n=329	n=130	n=876	n=245	n=107	n=32	n=254	n=46
No	90.5	85.7	94.3	94.3	97.6	96.2	96.1	96.7	99.1	90.6	97.6	97.8
Yes	9.5	14.3	5.7	5.7	2.4	3.9	3.9	3.3	0.9	9.4	2.4	2.2
**Violence perpetrated by an intimate partner**	n=197	n=48	n=257	n=72	n=338	n=143	n=878	n=244	n=296	n=65	n=347	n=68

Experience of sexual violence from partners							*		*			
No	80.2	87.5	79.8	81.9	83.1	81.1	83.6	77.1	82.4	69.2	81.6	72.1
Yes	19.8	12.5	20.2	18.1	16.9	18.9	16.4	22.9	17.6	30.8	18.4	27.9
Experience of physical violence from partners												
No	84.8	83.3	68.9	70.8	86.1	86.0	82.8	85.3	76.7	75.4	84.4	76.5
Yes	15.2	16.7	31.1	29.2	13.9	14.0	17.2	14.8	23.3	24.6	15.6	23.5
**Violence perpetrated by a non-partner**												
Experience of sexual violence from non-partners			*									
No	78.9	78.8	69.5	81.7	90.0	88.1	91.5	91.6	87.5	84.7	76.6	76.5
Yes	21.1	21.2	30.5	18.3	10.0	12.0	8.5	8.4	12.5	15.3	23.4	23.5

*p≤0.05; **p≤0.01; ***p≤0.001

For peer review only

STROBE Statement—Checklist of items that should be included in reports of ***cohort studies***

	Item No	Recommendation	Page No
**Title and abstract**	1	(*a*) Indicate the study's design with a commonly used term in the title or the abstract	Title
		(*b*) Provide in the abstract an informative and balanced summary of what was done and what was found	1-3
**Introduction**			
Background/rationale	2	Explain the scientific background and rationale for the investigation being reported	5-6
Objectives	3	State specific objectives, including any prespecified hypotheses	6
**Methods**			
Study design	4	Present key elements of study design early in the paper	7
Setting	5	Describe the setting, locations, and relevant dates, including periods of recruitment, exposure, follow-up, and data collection	8-9
Participants	6	(*a*) Give the eligibility criteria, and the sources and methods of selection of participants. Describe methods of follow-up	8-9
		(*b*) For matched studies, give matching criteria and number of exposed and unexposed	
Variables	7	Clearly define all outcomes, exposures, predictors, potential confounders, and effect modifiers. Give diagnostic criteria, if applicable	9-11
Data sources/ measurement	8*	For each variable of interest, give sources of data and details of methods of assessment (measurement). Describe comparability of assessment methods if there is more than one group	9-11
Bias	9	Describe any efforts to address potential sources of bias	
Study size	10	Explain how the study size was arrived at	8-9
Quantitative variables	11	Explain how quantitative variables were handled in the analyses. If applicable, describe which groupings were chosen and why	9-11
Statistical methods	12	(*a*) Describe all statistical methods, including those used to control for confounding	12
		(*b*) Describe any methods used to examine subgroups and interactions	
		(*c*) Explain how missing data were addressed	
		(*d*) If applicable, explain how loss to follow-up was addressed	
		(*e*) Describe any sensitivity analyses	
**Results**			
Participants	13*	(a) Report numbers of individuals at each stage of study—eg numbers potentially eligible, examined for eligibility, confirmed eligible, included in the study, completing follow-up, and analysed	13-15
		(b) Give reasons for non-participation at each stage	
		(c) Consider use of a flow diagram	
Descriptive data	14*	(a) Give characteristics of study participants (eg demographic, clinical, social) and information on exposures and potential confounders	13-15
		(b) Indicate number of participants with missing data for each variable of interest	
		(c) Summarise follow-up time (eg, average and total amount)	
Outcome data	15*	Report numbers of outcome events or summary measures over time	16-21

For peer review only

For peer review only - http://bmjopen.bmj.com/site/about/guidelines.xhtml

Main results	16	(*a*) Give unadjusted estimates and, if applicable, confounder-adjusted estimates and their precision (eg, 95% confidence interval). Make clear which confounders were adjusted for and why they were included<br>(*b*) Report category boundaries when continuous variables were categorized<br>(*c*) If relevant, consider translating estimates of relative risk into absolute risk for a meaningful time period	16-21
Other analyses	17	Report other analyses done—eg analyses of subgroups and interactions, and sensitivity analyses	
**Discussion**			
Key results	18	Summarise key results with reference to study objectives	22
Limitations	19	Discuss limitations of the study, taking into account sources of potential bias or imprecision. Discuss both direction and magnitude of any potential bias	26
Interpretation	20	Give a cautious overall interpretation of results considering objectives, limitations, multiplicity of analyses, results from similar studies, and other relevant evidence	22-26
Generalisability	21	Discuss the generalisability (external validity) of the study results	22-26
**Other information**			
Funding	22	Give the source of funding and the role of the funders for the present study and, if applicable, for the original study on which the present article is based	27

*Give information separately for exposed and unexposed groups.

Note: An Explanation and Elaboration article discusses each checklist item and gives methodological background and published examples of transparent reporting. The STROBE checklist is best used in conjunction with this article (freely available on the Web sites of PLoS Medicine at http://www.plosmedicine.org/, Annals of Internal Medicine at http://www.annals.org/, and Epidemiology at http://www.epidem.com/). Information on the STROBE Initiative is available at http://www.strobe-statement.org.

For peer review only

BMJ Open

Temporal shifts in HIV-related risk factors among cohorts of adolescent girls and young women enrolled in DREAMS programming: Evidence from Kenya, Malawi, and Zambia

Journal:	*BMJ Open*
Manuscript ID	bmjopen-2020-047843.R2
Article Type:	Original research
Date Submitted by the Author:	05-Nov-2021
Complete List of Authors:	Mathur, Sanyukta; Population Council<br>Heck, Craig ; Population Council<br>Kishor Patel, Sangram; Population Council India<br>Okal, Jerry; Population Council Kenya, HIV/AIDS<br>Chipeta , Effie; University of Malawi College of Medicine, Centre for Reproductive Health<br>mwapasa, victor; University of Malawi College of Medicine, Centre for Reproductive Health<br>Chimwaza, Wanangwa; University of Malawi College of Medicine, Centre for Reproductive Health<br>Musheke, Maurice; Independent Consultant<br>Mahapatra, Bidhubhusan ; Population Council India<br>Pulerwitz, Julie ; Population Council<br>Pilgrim, Nanlesta; ViiV Healthcare US
**Primary Subject Heading**:	HIV/AIDS
Secondary Subject Heading:	Epidemiology, Public health, Global health
Keywords:	Public health < INFECTIOUS DISEASES, HIV & AIDS < INFECTIOUS DISEASES, Epidemiology < INFECTIOUS DISEASES

SCHOLARONE™
Manuscripts

I, the Submitting Author has the right to grant and does grant on behalf of all authors of the Work (as defined in the below author licence), an exclusive licence and/or a non-exclusive licence for contributions from authors who are: i) UK Crown employees; ii) where BMJ has agreed a CC-BY licence shall apply, and/or iii) in accordance with the terms applicable for US Federal Government officers or employees acting as part of their official duties; on a worldwide, perpetual, irrevocable, royalty-free basis to BMJ Publishing Group Ltd ("BMJ") its licensees and where the relevant Journal is co-owned by BMJ to the co-owners of the Journal, to publish the Work in this journal and any other BMJ products and to exploit all rights, as set out in our licence.

The Submitting Author accepts and understands that any supply made under these terms is made by BMJ to the Submitting Author unless you are acting as an employee on behalf of your employer or a postgraduate student of an affiliated institution which is paying any applicable article publishing charge ("APC") for Open Access articles. Where the Submitting Author wishes to make the Work available on an Open Access basis (and intends to pay the relevant APC), the terms of reuse of such Open Access shall be governed by a Creative Commons licence – details of these licences and which Creative Commons licence will apply to this Work are set out in our licence referred to above.

Other than as permitted in any relevant BMJ Author's Self Archiving Policies, I confirm this Work has not been accepted for publication elsewhere, is not being considered for publication elsewhere and does not duplicate material already published. I confirm all authors consent to publication of this Work and authorise the granting of this licence.

For peer review only

Temporal shifts in HIV-related risk factors among cohorts of adolescent girls and young women enrolled in DREAMS programming: Evidence from Kenya, Malawi, and Zambia

Authors:

Sanyukta Mathur[1]*, Craig J. Heck[2], Sangram Kishor Patel[3], Jerry Okal[4], Effie Chipeta[5], Victor Mwapasa[5], Wanangwa Chimwaza[5], Maurice Musheke[6], Bidhubhusan Mahapatra[3], Julie Pulerwitz[1], Nanlesta Pilgrim[7]

Affiliations:

1. Population Council, Washington, DC, United States of America
2. Population Council, New York, NY, United States of America
3. Population Council, New Delhi, India
4. Population Council, Nairobi, Kenya
5. Centre for Reproductive Health, College of Medicine, Blantyre, Malawi
6. Independent consultant, Lusaka, Zambia
7. ViiV Healthcare, Research Triangle Park, NC, United States of America

Corresponding author:

Sanyukta Mathur
4301 Connecticut Avenue, NW
Suite 280
Washington, DC 20008
E-mail: smathur@popcouncil.org
Telephone: +1 202–237–9447
Fax: +1 202-237-8410

For peer review only

Abstract

Objectives: To assess temporal shifts in HIV risk factors among adolescent girls (AG, aged 15-19 years) and young women (YW, aged 20-24 years) in Kenya, Malawi, and Zambia.

Design: Prospective cohorts with two timepoints (Kenya: 2016, 2018; Malawi: 2017, 2018; Zambia: 2016/2017, 2018)

Setting: Community-based programming

Participants: 1247 AG (Kenya: 389, Malawi: 371, Zambia: 487) and 1628 YW (Kenya: 347, Malawi: 883, Zambia: 398)

Intervention: DREAMS (Determined, Resilient, Empowered, AIDS-free, Mentored and Safe), a multi-sectoral approach to reduce AGYW's HIV vulnerability by delivering a package of tailored, multi-layered activities and services

Primary and secondary outcome measures: HIV testing, sexually transmitted infection (STI) symptom experience, number of sexual partners, condom use (consistently, at last sex), transactional sex, experience of physical violence (from intimate partners) and sexual violence (from intimate partners and strangers/non-partners)

Results: Changes in HIV-related risk behaviors among DREAMS participants varied by age group and country. Among AG, HIV testing increased (Kenya and Zambia) and sexual violence from partners (in Kenya and Malawi) and non-partners (in Malawi) decreased. Among YW, HIV testing increased and STI experience decreased in Malawi; consistent condom use decreased in Kenya; transactional sex increased in Kenya and Zambia; and physical violence (in Malawi) and

For peer review only

sexual violence from partners (in Kenya and Malawi) and non-partners (all 3 countries) decreased over time.

Conclusions: Improvements in HIV testing and reductions in experiences of sexual violence were coupled with variable shifts in HIV-related risk behaviors among DREAMS participants in Kenya, Malawi, and Zambia. Additional consideration of AGYW's risk circumstances during key life transitions may be needed to address the risk heterogeneity among AG and YW across different contexts.

For peer review only

Strengths and limitations of this study

- Longitudinal cohorts of adolescent girls and young women in three sub-Saharan countries were followed across two time points, over a 12-16-month time period
- Comprehensive quantitative survey captured knowledge, attitudes, and HIV-related risk behaviors and exposure to a community-based multi-sectoral HIV prevention program
- Multivariate analyses examined change over time in factors associated with HIV acquisition
- Due to a lack of a comparison group, our findings could have been confounded by unobserved changes or secular trends in the study sites and/or the aging of the cohort

For peer review only

INTRODUCTION

Four decades into the HIV epidemic, adolescent girls and young women (AGYW, aged 15–24 years) remain at high risk for HIV. Globally, over 7,000 AGYW seroconvert weekly, and even though they comprise one-tenth of the population, 20% of sub-Saharan Africa's (SSA) seroconversions occur amongst AGYW.[1] In eastern and southern Africa, which has the world's highest regional prevalence (7.0% [5.9%-7.9%]),[2] the HIV epidemic is magnified by gendered disparities. AGYW's prevalence and incidence rates in Kenya, Malawi, and Zambia are double that (or more) of their male counterparts,[3-5] and when compared with all AGYW in SSA, all three countries have a prevalence that is equal to or greater than the regional average.[6] Though adolescent girls (AG, aged 15-19 years) and YW (YW, aged 20-24 years) are a priority population, the breadth, severity, and context of their HIV risk is not homogenous, for among females aged 15-29, HIV incidence is highest among AG in Kenya and YW in Malawi and Zambia.[6]

Underscoring these disproportionate HIV rates, a range of behavioral, biological, psycho-social, and structural factors fuel AGYW's vulnerability to HIV.[6] For instance, AGYW who have multiple sex partners are at higher risk for HIV due to increased exposure, [7 8] and experience of sexually transmitted infection (STI) symptoms has been linked to risky behaviors and biological vulnerability.[7 9] Physical and sexual violence, which is known to cause physical and psychological trauma, is associated with AGYW's HIV risk when perpetrated by partners[10-13] and non-partners.[11 13] In addition, non-marital partnerships are associated with age-disparate relationships and inconsistent condom use among AGYW and sexual concurrency among their male partners.[14-16] Further, economic disenfranchisement and limited social capital push some AGYW to seek out sexual partners for financial support; these transactional arrangements often include relationships with older men[17 18] and increase AGYW's HIV risk.[19-21] Negative life and social transitions—such as experiencing paternal loss,[22-25] divorce/widowhood,[7 26] and school dropout[7 18 27]—have also been associated with heighted engagement in risky sexual behaviors and precarious decision-making.

For peer review only

AGYW's HIV risk factors are multi-faceted and ecological, making them difficult to change using siloed, uni-component interventions.[28] In response, the U.S. President's Emergency Plan for AIDS Relief (PEPFAR), the Bill & Melinda Gates Foundation, Girl Effect, Johnson & Johnson, Gilead Sciences, and ViiV Healthcare initiated the Determined, Resilient, Empowered, AIDS-free, Mentored and Safe (DREAMS) Partnership in 2015, with the goal to reduce HIV acquisition among AGYW in 14 sub-Saharan countries (including Kenya, Malawi, and Zambia) and Haiti. Using public and private delivery channels, DREAMS delivers a comprehensive package of evidence-based activities that aim to provide vulnerable AGYW with knowledge, life skills, and access to social protection interventions (e.g., educational support). DREAMS also aspires to create an enabling environment that supports AGYW's health and development by offering events and services that engage parents and the broader community.[29-32] However, there is limited evidence about how this AGYW-focused approach, which extends beyond behavioral or health sector interventions, is influencing HIV-related risk among AGYW.

To contribute to the growing body of literature around comprehensive multi-sectoral HIV prevention programs, we assess temporal shifts in factors that have been previously linked to HIV-acquisition among DREAMS participants across three country contexts. Using an age-stratified approach to illuminate risk heterogeneities, we examine change over time in proximal behavioral, biological, and experiential HIV-related risk factors to assess if participation in the DREAMS program positively influenced participants' vulnerability. This study provides initial insight on the change over time in HIV-related risk factors among cohorts of AG and YW DREAMS participants in Kenya, Malawi, and Zambia, with the aim to inform the refinement or development of comprehensive multi-sectoral HIV prevention efforts.

METHODS

Program description

For peer review only

The DREAMS program aims to empower AGYW and reduce their risk of HIV and gender-based violence by delivering a package of tailored, multi-layered activities, services, and interventions.[29-32] The DREAMS approach has been described in detail elsewhere.[29] Briefly, the core package of interventions includes four components: (1) empowering AGYW – including condom promotion, PrEP provision (only approved and provided in Kenya at the time of study), post-violence care, HIV testing services, increasing access to voluntary family planning services, and social asset building (e.g. knowledge and skills to increase self-efficacy); (2) strengthening families through parenting/caregiver programs, education subsidies and combination socioeconomic approaches; (3) mobilizing communities to address harmful community norms and practices, and school based HIV and violence prevention; and (4) reducing risk of AGYW's sexual partners by increasing access to HIV prevention and care services. DREAMS implementing partners were expected to reach one-half of all vulnerable AGYW in their study communities during the study implementation time period. [29] Country teams recruited AGYW based on a range of factors that constituted vulnerability (e.g., being out-of-school, orphaned, history of violence experience, STI experience, etc.). The program uses designated spaces within the community, where AGYW engage, often weekly, in the DREAMS activities. The safe spaces, which act as a programmatic hub and link AGYW peers and mentors, facilitated instruction on health and life skills, and referrals to youth-friendly health services and social protection interventions.

PEPFAR determined the target DREAMS program areas, with input from local officials and other stakeholders, based on their high HIV prevalence and additional factors, such as high poverty levels, population density, and/or population growth. In each site, implementing partners (IP) used systematic selection processes to identify (e.g., visiting and screening households in their community catchment area[33]), recruit, and enroll eligible AGYW beneficiaries.[34-39] In Malawi, an additional eligibility criterion was out-of-school status. In each country context, a primary package of interventions[37 39 40] was delivered, with the expectation that all DREAMS participants would receive this primary package and additional interventions, depending on individual need or vulnerability.

For peer review only

Study sites

In Kenya and Zambia, the sites for this study were purposively selected, in consultation with PEPFAR, local stakeholders, and implementing partners, to be representative of key geographic characteristics (e.g., urban/peri-urban/rural) of DREAMS program communities in each country. In Malawi, the study sites were selected from each of the two districts involved in DREAMS programming at the time. In total, the study was conducted in two sites in Kisumu County, Kenya; four sites in Malawi's Zomba and Machinga districts; and two sites in Lusaka and Ndola, Zambia.

Study population

Two rounds of data were collected with study participants. Round one data was collected in 2016/2017. Eligible survey participants were females aged 15–24 years residing in the study catchment area who were enrolled in the DREAMS program and intended to stay in the area for the subsequent year. At round one, study participants were identified using an age-stratified random sample from the DREAMS program beneficiary rosters (Kenya [n=5997], Malawi [proportionate to the differently sized districts, n=4738]) prepared by the program IPs, whereas a census of all AGYW enrolled in DREAMS was conducted in Zambia. Potential participants were approached by study staff and invited to take part in the study. Using a conservative estimate (i.e., 50%) of baseline prevalence for the primary outcomes (e.g., HIV testing), the samples were powered at 80% to detect a minimal 10-20% percentage change. In Kenya, 474 AG and 440 YW were interviewed from October 2016 to February 2017. In Malawi, 528 AG and 1134 YW were interviewed from July 2017 to September 2017. In Zambia, 585 AG and 479 YW were interviewed from November 2016 to April 2017. Twenty respondents in Kenya, 33 in Zambia, and 3 in Malawi refused to participate due to lack of parental consent, in ability to locate the participant or limited time availability/other events that prohibited participation.

For peer review only

A second round of data was collected with round one respondents in 2018 after approximately 12-16 months of exposure to DREAMS. At round 2, 80.5% of Kenya's respondents (389 AG and 347 YW) were re-interviewed from April-June 2018, 75.5% of Malawi's respondents (371 AG and 883 YW) were re-interviewed from September-November 2018, and 79.8% of Zambia's respondents (487 AG and 398 YW) were re-interviewed from April-May 2018. Overall, loss to follow-up occurred due to extended or permanent out-migration from the study communities, as confirmed by the program team and through repeated attempts to recontact participants by the research team. Those not re-interviewed had re-located to towns outside study settings due to family, business or school reasons. Comparison of baseline characteristics and outcome values between respondents who were followed-up and those who were lost show some differences by age group or country, but do not show any systematic patterns of loss (see Supplemental Table 1). For instance, Kenyan AG who could not be re-interviewed were more likely to be out of school ($p \leq 0.05$) and orphaned ($p \leq 0.01$), but this was not the case for YW in Kenya or AG and YW in Malawi and Zambia.

Study procedures

Respondent consent (or parent/guardian consent and minor assent) was obtained before each round of data collection. Using a tablet-based survey instrument in the respondent's chosen language (Kiswahili and Luo in Kenya, Chichewa and Yao in Malawi, Bemba and Nyanja in Zambia, or English in all countries), female interviewers captured respondents' sociodemographic characteristics; program engagement and exposure; and knowledge, attitudes, and behaviors related to HIV/AIDS. Interviews were conducted in private yet convenient locations to the respondents (e.g., room in respondent's home, nearby field, or nearby community center), and out of earshot of parents, guardians, or other community members.

Measures

For peer review only

Cohorts' characteristics

The study cohorts only included respondents who provided information at both round one and two. We explored the cohorts' socio-demographic characteristics—such age (15-19 vs. 20-24) and marital (ever [currently, formerly] vs. never married), schooling (in school vs. out of school), and orphanhood (both parents alive vs. at least one parent deceased) status, and using round 2 data only, we examined their participation in social asset building activities and exposure to (i.e., being offered the service/activity by DREAMS staff) various DREAMS program components—such as HIV testing services (HTS), health services (condoms, PrEP, post-violence care, contraception, STI testing, pregnancy consultation), educational support (money for fees, uniforms, transport, or other help with schooling expenses in Kenya and Zambia; back to school support in Malawi), and economic support (cash transfer in Kenya and Zambia, food support in Malawi). The study sites remained the same across the two rounds of data collection (Kenya: Nyalenda A, Kolwa East; Malawi: Zomba, Machinga; Zambia: Lusaka, Ndola).

We assessed engagement in DREAMS' primary intervention by asking respondents if they participated in the safe space groups (yes vs. no) by round 2. Additionally, to examine the level of exposure to all DREAMS activities, we asked participants about the frequency and duration (short [<1 week, 1 week] vs. long [1 month, several months]) of their program interruptions. Among those that experienced periods of non-participation, we used the median number of interruptions (3 in all countries) to classify respondents as experiencing few (<median) or many (≥median) interruptions.

Outcomes

We focused on factors that are known to facilitate or mitigate AGYW's HIV risk. HIV testing in the last 12 months and experiencing STI symptoms (genital warts, painful urination, vaginal discharge, genital ulcer) in the last 6 months were coded as binary (yes vs. no) variables. Among those who had sexually debuted, we truncated respondents' number of sexual partners in the last 12 months into a categorical variable (0, 1, 2+). We considered respondents to be consistent condom users if they were currently in a

For peer review only

sexual relationship and in the last 12 months, they always used condoms with their primary and (if applicable) secondary partner; using this same schema, we also assessed condom use at last sex. For Zambia only, the condom use variables were only asked of respondents who were currently sexually active with their primary and (if applicable) secondary partner.

Using a binary (yes vs. no) variable, we identified respondents as transactional sex engagers if they had engaged in sex with a stranger or casual partner to receive cash or kind; somewhere to stay; support or money for their children or family; drugs, food, cosmetics, clothes, a cellphone, airtime, transportation; somewhere to sleep for the night; financial support for bills or school fees; or anything else the they could not afford by themselves in the past 12 months.[41-43]

We measured intimate partner violence (IPV) among all respondents who reported having a sexual partner in the last 12 months and classified respondents as having experienced sexual IPV if they disclosed that in the last 12 months, they had sex or performed other sexual acts against their will because a current or previous boyfriend or partner used physical coercion, threats and intimation, or force. We coded respondents as having experienced physical IPV if they had been slapped or had something thrown at them; pushed or shoved; hit with a fist or another dangerous object; kicked, dragged, beaten, choked, or burnt; or threatened with a gun, knife, or other weapon by a current or previous boyfriend or partner in the last 12 months.[11]

Lastly, questions on non-partner sexual violence were asked of all AGYW. We classified respondents as having experienced non-partner sexual violence if they disclosed that in the last 12 months, a person other than their boyfriend or partner perpetrated unwanted sex by using persuasion or force—whether successful or unsuccessful; forced sex while they were under the influence of drugs or alcohol and too impaired to refuse; or forced sex by two or more men—when the respondent was either sober or under the influence of drugs or alcohol.[11]

Data analysis

For peer review only

We conducted bivariate chi-squared analyses to examine the change in outcome measures between data collection rounds. To account for intra-individual correlation, we used generalized estimating equations (with a Poisson distribution) to examine temporal changes, control for characteristics (study site and marital, schooling, and orphanhood status at round one), and report measures of association (Adjusted Incident Rate Ratio [95% Confidence Interval]). Adjusted models were run with and without adjusting for interruptions in program participation variable to assess if level of program exposure influenced outcomes. To achieve model convergence, we removed marital status from the model examining transactional sex among Zambian AG. We ran all analyses using Stata (version 15.0).

Patients and public involvement

This study actively engaged the study community and program implementing partners in the research process. Approval for the study was sought from community leaders and representatives in each setting during study planning. Research questions and tools were vetted with program implementing partners prior to administration. Post data collection, dissemination meetings were conducted with program implementing partners and community leaders to share findings and implications for HIV prevention and other programming for AGYW in the study communities. Short study results briefs and presentation slide decks were developed and shared with program implementing partners and other collaborators for dissemination of research results in each setting with key leaders and decision-makers.

RESULTS

Cohorts' characteristics

Table 1 presents the socio-demographic characteristics of the cohorts in Kenya, Malawi, and Zambia. Overall, the mean baseline age was between 17 (Kenya, Zambia) and 18 (Malawi) for AG and about 22 for YW (all countries). At round one, most (>50%) Kenyan and Zambian respondents were unmarried.

For peer review only

Conversely, marriage was common among Malawian AG (62%) and YW (89%) at round one. The majority of AG in Kenya and Zambia were in school (>60%) at round one, and over 50% of Zambian YW were still in school at Round 2. Across all socio-demographic characteristics, there were modest shifts over time, as only a few age groups in certain countries had significant increases in out-of-school (Kenyan AG, Zambian AG and YW) and ever-married (Malawian AG and YW) status.

Respondents had nearly universal exposure to DREAMS' primary social asset building intervention: safe space groups, and exposure to HTS was high among Kenyan AG (96.1%) and YW (91.4%) but lower among AG and YW in Malawi and Zambia. With the exception of Kenya, where approximately ≥50% of AG and YW were exposed to educational support or economic support, exposure to other health services, educational support, and economic support was low (e.g., no YW in Malawi and 17% in Zambia had received some economic support).

On average, 75% of respondents experienced some form of program interruption, with just under 2 in 3 reporting many (i.e., ≥3) interruptions that last less than a week or more than a month. In Zambia for instance, 75% of AG noted there were times when they were not able to participate in DREAMS with 29% reporting at least 5 interruptions lasting for a month or more. The main reasons for periods of non-participation included being away at school (Kenyan AG [58.9%]), sickness (Malawian AG [51.5%] and YW [47.1%]) and being away from the community (Kenyan YW [32.9%] and Zambian AG [29.0%] and YW [37.4%]).

For peer review only

Table 1: Characteristics of cohorts in Kenya, Malawi, and Zambia

Characteristics	Kenya				Malawi				Zambia			
	15-19 n=389		20-24 n=347		15-19 n=371		20-24 n=883		15-19 n=487		20-24 n=398	
	Round 1	Round 2	Round 1	Round 2	Round 1	Round 2	Round 1	Round 2	Round 1	Round 2	Round 1	Round 2
**Mean Age (SD)**	16.6 (1.3)	18.3 (1.8)	21.6 (1.4)	22.6 (1.9)	18.0 (1.2)	19.3 (1.3)	21.9 (1.4)	23.1 (1.6)	16.8 (1.4)	18.1 (1.6)	21.6 (1.3)	22.5 (1.4)
**Marital Status**					***		***					
Ever Married	4.4	6.4	44.4	49.6	62.3	74.4	88.9	93.7	2.5	3.1	8.5	11.8
Never married	95.6	93.6	55.6	50.4	37.7	25.6	11.1	6.3	97.5	96.9	91.5	88.2
**Current Schooling Status**	**								***		**	
In school	84.3	75.3	32.9	27.1	-	-	-	-	77.6	60.6	61.6	52.3
Out of school	15.7	24.7	67.1	72.9	-	-	-	-	22.4	39.4	38.4	47.7
**Orphanhood**												
Both parents alive	62.5	58.1	42.4	38.9	70.9	69.5	61.4	60.8	64.9	62.6	55.5	54.3
Lost at least one parent	37.5	41.9	57.6	61.1	29.1	30.5	38.6	39.2	35.1	37.4	44.5	45.7
**Program Participation/Exposure**												
Social Asset Building	-	91.3	-	92.2	-	96.5	-	97.3	-	90.4	-	91.5
Offered HTS	-	96.1	-	91.4	-	62.0	-	65.1	-	80.9	-	73.9
Offered other health service	-	40.2	-	44.4	-	29.1	-	30.5	-	36.2	-	34.4
Educational Support	-	66.8	-	57.6	-	1.4	-	1.1	-	46.4	-	21.9
Economic Support	-	49.6	-	57.6	-	1.0	-	0.0	-	25.1	-	16.6
**Interruptions**												
None	-	27.5	-	26.5	-	21.0	-	24.0	-	25.1	-	29.4
<3 interruptions of ≤1 week	-	6.9	-	6.3	-	9.7	-	7.1	-	5.5	-	6.5
<3 interruptions of ≥1 month	-	7.7	-	4.6	-	3.2	-	3.4	-	3.5	-	3.3
≥3 interruptions of ≤1 week	-	23.1	-	28.8	-	22.1	-	28.0	-	37.0	-	30.4
≥3 interruptions of ≥1 month	-	34.7	-	33.7	-	43.9	-	37.5	-	28.9	-	30.4

*p≤0.05; **p≤0.01; ***p≤0.001 for marital, schooling, and orphanhood status
Other health services includes condoms, PrEP (where available), post-violence care, contraception, STI testing, pregnancy consultation
Educational support includes money for fees, uniforms, transport or other help with schooling expenses (Kenya, Zambia) or back to school support (Malawi)
Economic support includes cash transfer (Kenya, Zambia) and food support (Malawi)
Program participation/exposure and interruptions measured only at Round 2
Malawi's sample only included out-of-school participants; hence, current schooling status is not reported for Malawi's cohorts

For peer review only

Kenya: Unadjusted and adjusted transitions in AG and YW's HIV risk factors

Table 2 displays the unadjusted and adjusted changes in HIV risk factors among Kenyan AG and YW. Among AG, HIV testing increased (81.5% to 96.4%, p≤0.001, aIRR=1.18 [1.02-1.37]) and reports of sexual IPV decreased (19.9% to 6.4%, p≤0.001, aIRR=0.32 [0.18-0.56]). YW reported a decrease in consistent condom use (31.0% to 19.0%, p=0.004, aIRR= 0.57 [0.40-0.81]), sexual IPV (20.2% to 10.5%, p=0.001, aIRR=0.51 [0.34-0.75]), and non-partner sexual violence (30.6% to 13.5%, p≤0.001, aIRR= 0.44 [0.32-0.61]) and an increase in transactional sex (5.7% to 10.0%, p=0.044, aIRR= 1.73 [1.04-2.89]).

For peer review only

Table 2: Temporal shifts in behavioral, biological, and experiential HIV risk factors among AGYW in Kenya

	Aged 15-19 n=389				Aged 20-24 n=347			
	Round 1	Round 2	p-value	aIRR (95% CI)	Round 1	Round 2	p-value	aIRR (95% CI)
**HIV Service Use**								
HIV Testing in last year	81.5	96.4	**<0.001**	**1.18 (1.02-1.37)**	96.3	97.1	0.525	1.01 (0.88-1.15)
**Sexual Behavior**								
STI Symptoms	14.4	14.9	0.839	1.04 (0.72-1.49)	23.1	21.0	0.522	0.91 (0.68-1.22)
Number of sex partners			0.531	1.00 (0.81-1.23)			0.259	1.00 (0.87-1.15)
0	11.1	8.7			5.1	2.8		
1	73.0	78.6			79.8	83.8		
2+	15.9	12.7			15.1	13.4		
Consistent condom use	52.5	45.8	0.395	0.78 (0.52-1.14)	30.7	19.0	**0.004**	**0.57 (0.40-0.81)**
Condom use at last sex	74.6	64.4	0.172	0.79 (0.54-1.15)	46.3	38.0	0.071	0.77 (0.59-1.01)
Transactional sex	9.5	8.1	0.664	0.91 (0.49-1.68)	5.7	10.0	**0.044**	**1.73 (1.04-2.89)**
**Violence Perpetrated by an Intimate Partner**								
Sexual	19.8	6.4	**<0.001**	**0.32 (0.18-0.56)**	20.2	10.5	**0.001**	**0.51 (0.34-0.75)**
Physical	15.2	14.7	0.866	0.96 (0.61-1.50)	31.1	25.0	0.099	0.78 (0.60-1.02)
**Violence Perpetrated by a Non-partner**								
Sexual	21.1	19.5	0.593	0.93 (0.69-1.25)	30.5	13.5	**<0.001**	**0.44 (0.32-0.61)**

aIRR = Adjusted Incidence Rate Ratios, 95% CI = 95% Confidence Interval

aIRR adjusts for location and round one marital, schooling, and orphanhood status

Bolded cells indicate p≤0.05

Malawi: Unadjusted and adjusted changes in AG and YW's HIV risk factors

Table 3 contains the unadjusted and adjusted shifts in HIV risk factors among Malawian AG and YW. AG reported more HIV testing (85.4% to 94.5%, p≤0.001); they also reported fewer sex partners (2+ sex partners: 9.4% to 1.8%, p≤0.001) and experiences of sexual violence from partners (16.9% to 5.9%, p≤0.001, aIRR=0.35 [0.21-0.57]) and non-partners (10.0% to 4.3%, p=0.003, aIRR= 0.43 [0.25-0.74]). However, the associations for HIV testing and number of sexual partners became null in the multivariable models. Among YW, increases in HIV testing (82.5% to 91.7%, p≤0.001, aIRR= 1.11 [1.01-1.22]) were coupled with decreases in STI symptoms (31.8% to 22.1%, p≤0.001, aIRR= 0.69 [0.60-0.81]), number of sexual partners (2+ sex partners: 6.2% to 3.2%, p≤0.001), and experiences of sexual IPV (16.4% to 4.5%, p≤0.001, aIRR= 0.27 [0.20-0.38]), physical IPV (17.2% to 11.8%, p=0.001, IRR= 0.68 [0.55-0.85]), and non-partner sexual violence (8.5% to 1.8%, p≤0.001, aIRR= 0.21 [0.13-0.36]). In the multivariable models, the association for number of sexual partners became null.

For peer review only

Table 3: Longitudinal changes in behavioral, biological, and experiential HIV risk factors among Malawian AGYW

	Aged 15-19 n=371				Aged 20-24 n=883			
	**Round 1**	**Round 2**	**p-value**	**aIRR (95% CI)**	**Round 1**	**Round 2**	**p-value**	**aIRR (95% CI)**
**HIV Service Use**								
HIV Testing in last year	85.4	94.5	**<0.001**	1.11 (0.95-1.29)	82.5	91.7	**<0.001**	**1.11 (1.01-1.22)**
**Sexual Behavior**								
STI Symptoms	25.6	22.4	0.302	0.87 (0.67-1.14)	31.8	22.1	**<0.001**	**0.69 (0.60-0.81)**
Number of sex partners			**<0.001**	1.00 (0.86-1.17)			**<0.001**	1.07 (0.98-1.18)
0	20.4	12.3			23.5	14.3		
1	70.2	86.0			70.3	82.5		
2+	9.4	1.8			6.2	3.2		
Consistent condom use	11.8	6.8	0.056	0.56 (0.31-1.01)	5.0	5.4	0.743	0.89 (0.57-1.38)
Condom use at last sex	20.2	20.3	0.967	0.98 (0.66-1.43)	14.0	15.7	0.395	1.01 (0.80-1.27)
Transactional sex	2.4	3.5	0.412	1.40 (0.62-3.17)	3.9	3.0	0.281	0.76 (0.48-1.22)
**Violence Perpetrated by an Intimate Partner**								
Sexual	16.9	5.9	**<0.001**	**0.35 (0.21-0.57)**	16.4	4.5	**<0.001**	**0.27 (0.20-0.38)**
Physical	13.9	11.2	0.29	0.80 (0.55-1.17)	17.2	11.8	**0.001**	**0.68 (0.55-0.85)**
**Violence Perpetrated by a Non-partner**								
Sexual	10.0	4.3	**0.003**	**0.43 (0.25-0.74)**	8.5	1.8	**<0.001**	**0.21 (0.13-0.36)**

aIRR = Adjusted Incidence Rate Ratios, 95% CI = 95% Confidence Interval

aIRR adjusts for location and round one marital and orphanhood status. We did not control for schooling because out-of-school status was a participation requirement in Malawi.

Bolded cells indicate p≤0.05

For peer review only

Zambia: Unadjusted and adjusted shifts in AG and YW's HIV risk factors

Table 4 presents the unadjusted and adjusted transitions in HIV risk factors among Zambian AG and YW. For AG, HIV testing increased over time (47.4% to 74.1%, p≤0.001, aIRR=1.56 [1.34-1.82]). YW reported more HIV testing (67.6% to 76.1%, p=0.007) and transactional sex (2.4% to 5.8%, p=0.049) and fewer experiences of non-partner sexual violence (23.4% to 14.1%, p=0.001, aIRR= 0.60 [0.44-0.82]). Increases in HIV testing and transactional sex exhibited a trend toward significance in the adjusted models.

Table 4: Changes over time in behavioral, biological, and experiential HIV risk factors among AGYW in Zambia

	**Aged 15-19 n=487**				**Aged 20-24 n=398**			
	**Round 1**	**Round 2**	**p-value**	**aIRR (95% CI)**	**Round 1**	**Round 2**	**p-value**	**aIRR (95% CI)**
**HIV Service Use**								
HIV Testing in last year	47.4	74.1	**<0.001**	**1.56 (1.34-1.82)**	67.6	76.1	**0.007**	1.13 (0.99-1.28)
**Sexual Behavior**								
STI Symptoms	12.7	11.5	0.556	0.90 (0.65-1.25)	15.8	15.8	1.000	1.00 (0.75-1.34)
Number of sex partners			0.111	0.92 (0.74-1.16)			0.450	0.94 (0.81-1.10)
0	7.6	16.1			15.0	17.0		
1	85.9	76.5			72.1	73.3		
2+	6.6	7.4			13.0	9.8		
Consistent condom use	42.5	38.8	0.706	0.78 (0.45-1.35)	42.3	33.3	0.167	0.72 (0.47-1.09)
Condom use at last sex	60.0	55.2	0.629	0.87 (0.52-1.44)	52.6	45.5	0.287	0.81 (0.57-1.16)
Transactional sex	0.9	3.7	0.163	3.76 (0.45-31.67)	2.4	5.8	**0.049**	2.50 (0.99-6.35)
**Violence Perpetrated by an Intimate Partner**								
Sexual	17.6	17.9	0.923	1.04 (0.73-1.47)	18.4	14.4	0.144	0.79 (0.56-1.11)
Physical	23.3	23.9	0.859	1.03 (0.77-1.36)	15.6	12.1	0.172	0.75 (0.52-1.09)
**Violence Perpetrated by a Non-partner**								
Sexual	12.5	10.9	0.425	0.87 (0.61-1.23)	23.4	14.1	**0.001**	**0.60 (0.44-0.82)**

aIRR = Adjusted Incidence Rate Ratios, 95% CI = 95% Confidence Interval

aIRR adjusts for location and round one marital, schooling, and orphanhood status

Bolded cells indicate p≤0.05

To achieve model convergence, the transactional sex model for AG does not include the respondent's marital status at round one.

For peer review only

DISCUSSION

We present one of the first multi-country analyses examining temporal changes in behaviors and experiences associated with HIV acquisition among cohorts of AG and YW enrolled in DREAMS in Kenya, Malawi, and Zambia. Our analysis uses prospective data and presents stratified analyses by age group. Our findings illuminate heterogeneous shifts in HIV related risk factors between AG and YW, and across countries. Over time, AG who were part of DREAMS report increases in HIV testing (Kenya and Zambia) and decreases in sexual IPV (Kenya and Malawi) and non-partner sexual violence (Malawi). HIV testing increased among YW in Malawi and Zambia (borderline significance); Malawian YW also had fewer experiences of STIs. Additionally, YW report significant or marginal increases in transactional sex (Kenya and Zambia) and decreases in consistent condom use (Kenya). YW engaged in DREAMS also report lower sexual IPV (Kenya and Malawi), lower physical IPV (Malawi), and non-partner sexual violence (Kenya, Malawi, and Zambia). Some emerging research in Uganda report that DREAMS programming contributed to the reduction of sexual risk behaviors; though, the effects differed by age group.[44] While the DREAMS programming models included considerations for participant age (e.g. providing education support to AG, and livelihoods support to YW), our findings indicate that additional tailoring of comprehensive HIV prevention packages may be needed to address the risk heterogeneity of AG and YW across different contexts.[43]

Increases in HIV testing rates among Kenyan and Zambian AG and Malawian YW, including a notable increase among Zambian YW, are promising as HIV testing is the first step in the HIV care cascade and remains a key prevention strategy. Our data show that close to 75% (or greater) of AG and YW self-reported testing for HIV in the last year. In comparison, recent national averages of self-reported HIV testing are much lower (Kenya: 35% of AG, 64% of YW; Malawi: 28% of AG, 52% of YW; Zambia: 29% of AG, 53% of YW).[3 4 45] These results likely point to PEPFAR, local national HIV programs and policies, and DREAMS' focus on expanded access to HIV testing services to AGYW in the DREAMS study communities.[29] Though only observed within one group, it is also encouraging to see that Malawian

For peer review only

YW reported fewer STI symptoms over time, as STIs increase AGs' and YW's biological vulnerability to HIV.[46 47] Within DREAMS, establishing, providing, and strengthening youth-friendly health services was a key part of the intervention package. AG and YW were primarily linked to these services through discussions and referrals provided in the safe space groups. This community-based platform may have been particularly helpful in bringing services to marginalized AGYW, who would otherwise be hard to reach with HIV and reproductive health programming.[48]

Decreases in experience of sexual IPV over time are also promising. Previous research supports the direct relationship between HIV acquisition and sexual IPV, primarily because it facilitates other risk behaviors and mechanisms (e.g., decrease in self-efficacy and increases in multiple partnerships, transactional sex, substance abuse, negative mental health outcomes, and biological vulnerability due to trauma).[10 11 49 50] Compared with data from nationally-representative surveys among ever-married women aged 15-49, the baseline prevalence for sexual IPV among AGYW in our cohorts are high.[45 51] The DREAMS programs were conducted in high-risk communities with the most-at-risk AGYW, which may account for the higher experiences of sexual violence than the national average. Over time, we find that AG and YW in Kenya and Malawi reported significantly fewer incidents of sexual IPV over time. Since it is also perpetrated by a trusted individual, physical IPV has similar mediating pathways.[10 52] In, almost, all countries and age groups, physical IPV reduced, yet the reduction was only significant among Malawian YW and could be because most Malawian YW were ever married at round one and, therefore, had the greatest opportunity for positive change; they also had the highest mean age, which could have translated into greater agency and relationship power. Additional evidence is needed to support or refute these hypotheses. Zambian AG and YW reported no significant shifts related to either IPV outcome; this warrants further investigation as to how IPV is perceived among Zambian AGYW and how programs are attempting to address it.

Sexual violence perpetrated by non-partners (assault and rape) is understudied; in our analysis, the baseline prevalence of non-partner sexual violence ranged from 10.0% to 21.1% among AG and 8.5% to 30.5% among YW. These totals are likely underestimated due to the topic's sensitive nature, which can

For peer review only

trigger recollections of sadness and trauma; regardless, compared with other non-partner sexual violence metrics (9.1% prevalence among Johannesburg AG,[13] 11.9% [8.5%-15.3%] lifetime prevalence among African women[52]), these baseline totals are high. Other studies have found that surviving non-partner sexual violence can lead to a myriad of behaviors or outcomes that heighten HIV risk (e.g., substance abuse, poorer sexual and reproductive health and mental health outcomes, and increased experience of STIs).[11 13 52] In our study, it is reassuring that we observed appreciable drops in non-partner sexual violence among DREAMS participants across all age groups and countries, which hopefully signifies community-wide shifts in accountability around violence and safer spaces for AGYW. DREAMS' multi-sectoral approach could explain some of the notable decrease in intimate and non-partner violence. Gender-inequitable social norms and patriarchal power structures, often, create environments where men are emboldened to perpetrate violence, both against their intimate and strangers/casual partners. To disrupt these norms, DREAMS programming made a concerted effort to create an enabling environment for AGYW by challenging existing power dynamics and addressing gender-based violence—both within the safe space groups and the broader programming community.[29-32] However, since all girls were exposed to anti-violence programming and our study does not include any data from men or other community members, this study cannot disentangle its effects from other activities. To strengthen our understanding, future research should consider collecting attitudes and behaviors related to non-partner violence from various ecological strata, and further examination is required to unpack the specific programming components and pathways that may have contributed to these significant changes.

For peer review only

We do not find substantial shifts in other preventive behaviors (i.e., increase in condom use, decrease in multiple partnerships). On average, consistent condom use and condom use at last sex decreased between survey rounds, with the greatest decrease occurring among Kenyan YW. While previous research shows that proper condom use is protective against HIV acquisition,[53-55] condom uptake among adolescent and youth populations is consistently suboptimal,[53 56-59] a pattern that is reinforced by our findings. A post-hoc trend decomposition analysis suggests that condom use behaviors may have shifted for YW as they

entered martial partnerships (results not shown). Previous literature suggests that low condom use among AG and YW could be a product of failed negotiations fueled by low self-efficacy, relationship power, and gender-inequitable norms and practices.[6 18 43 60 61] Alternatively, condom use could have decreased due to changes in contraceptive use—such as moving from condoms to hormonal contraceptives—or desires to have children with their spouse.[62-64] Multiple factors and motivations may be contributing to shifts in condom use among AG and YW, especially those in recent unions. For HIV prevention programming, it will be critical to consider approaches to ensure that AGYW and their partners employ strategies (e.g., couple HIV testing and counseling, safer conception practices) to mitigate HIV risk as they transition in and out of relationships and have children.

Among Kenyan and Zambian YW, we found significant or trending increases of transactional sex over time. Previous research shows that transactional sex can increase women's HIV risk.[19 20 41 65 66] To avert poverty, homelessness, and food insecurity, some may turn to transactional sex to survive or to obtain desired commodities.[17 18 67] AGYW's economic disenfranchisement is characterized by a constellation of factors—including gendered discrimination and lack of social and business capital[68]—that make it difficult to AGYW to access the economic resources or earn a living wage. While DREAMS provided some economic activities (e.g., financial literacy and education) and strengthened access to some economic resources (e.g., access to school bursary programs or savings accounts), it is possible that broader partnerships or deeper investments are needed to make gainful employment/entrepreneurship possible for AGYW. Additionally, it is possible that the implementation of the socio-economic interventions within DREAMS may have faltered. Research in South Africa on the DREAMS Partnership found that rollout of program components that required multi-sectoral collaborations (especially those beyond the health sector) were complex and challenging in a community-based setting.[69] Other structural intervention programs have reported reductions in economically-driven sex, risky sexual practices (e.g., condomless sex, multiple sex partners).[70-73] These positive shifts show that intersectional approaches can

For peer review only

lead to meaningful change and should continue be considered by future HIV prevention programs for AGYW.[74-76]

While we found some promising results, this study is not without limitations. Across the three countries, 17-25% of the respondents could not be re-interviewed at round two; however, this level of attrition is consistent with other community-based safe space programs.[77] While our original sample size calculations were estimated anticipating this loss to follow-up and the lost-to-follow-up analysis (Supplemental Table 1) shows similar distributions for most baseline characteristics and outcomes, this attrition could have introduced selection bias. To avoid this in future studies, robust retention techniques should be employed. In Zambia, we could only assess condom use among a specific subset of sexually active respondents, thus our findings should not be generalized to all sexually active AGYW. All the data were self-reported, which is prone to social desirability and recall bias. In our survey, we assessed level of program engagement/continuity of program exposure but adjusting for program exposure did not change the regression results; additional attention may be needed for more robust measures on program exposure. An additional challenge was the lack of a control group; since the DREAMS program was implemented over time, it continued to enroll AGYW from nearby communities and catchment areas, making it hard to find an appropriate counterfactual group. It also means our findings could be prone to confounding by secular trends and the aging of the cohorts in the study contexts. Subsequent analyses using modeling techniques using age as the timescale to create a control group could further help to reduce bias.

CONCLUSION

Recent large-scale HIV prevention efforts are currently underway to reduce the disproportionate burden of HIV among AGYW in sub-Saharan Africa. Our longitudinal analysis assessed temporal change in key behaviors and experiences associated with HIV acquisition among Kenyan, Malawian, and Zambian AGYW cohorts nested within DREAMS, a multi-sectoral HIV prevention program. These analyses found a positive effect on some HIV risk factors, such as HIV testing, non-partner sexual violence, and physical

For peer review only

and sexual intimate partner violence, but variable improvements in sexual risk behaviors. Our findings contribute to the growing body of evidence that is assessing the impact of community-based multi-sectoral HIV prevention programming for AGYW.

For peer review only

Acknowledgements

The authors would like to acknowledge the implementing partners in Kenya (Aphia Plus/PATH, I Choose Life, Make Me Smile), Malawi (OneCommunity), and Zambia (PACT, CMMB, Marie Stopes Zambia), and our research team coordinators and data collectors for their support during the data collection. We would like to acknowledge support from Population Council colleagues who provided support in the preparation of this manuscript, and our deepest gratitude goes to the study participants. This work would not have been possible without the active support of the PEPFAR DREAMS teams in Kenya, Malawi, and Zambia.

Contributors

SM, NP, and JP conceptualized the study. SM, NP, CJH, SKP, and BM devised and implemented the analysis plan. EC, VM, WC, JO, and MM supported the study design and led the data collection. SM and CJH led the manuscript writing. All authors reviewed and revised the manuscript.

Funding

Funding support for the Kenya and Zambia studies was provided by the Bill & Melinda Gates Foundation (OPP1136778, DREAMS Implementation Science Research). Funding for the Malawi study was provided by the generous support of the American people through the United States President's Emergency Plan for AIDS Relief (PEPFAR) and the United States Agency for International Development (USAID) under Project SOAR (Cooperative Agreement AID–OAA–A–14–00060). The contents of this manuscript are the sole responsibility of the authors and do not necessarily reflect the views of PEPFAR, USAID, or the United States Government.

Competing interests

For peer review only

NP works full-time for ViiV Healthcare. ViiV Healthcare did not fund the study. The contents of the manuscript do not reflect the views of ViiV Healthcare. SM, CJH, SKP, JO, EC, VM, WC, MM, BM, and JP declare no conflicts of interest.

Patient consent for publication

Not required

Provenance and peer review

Not commissioned, externally peer reviewed

Data availability statement

All the underlying data relevant to the study are fully available on Dataverse.

Ethics statement

At each survey round, we collected written informed consent from adult participants (those above the age of consent in each country) and emancipated minors (when applicable). If the participant was considered a minor, we obtained parental consent and participant assent. The protocols for these studies were reviewed and approved by Population Council's Institutional Review Board (protocol # 744 and 745); Kenya's Kenyatta National Hospital/University of Nairobi Ethics (P384/05/2016) and Research Committee and National Commission for Science, Technology, and Innovation; Malawi's College of Medicine Research and Ethics Committee at the University of Malawi (PP.01/17/2095); and Zambia's ERES Converge Institutional Review Board (2016- May-016) and National Health Research Authority. Participants were compensated 300 Kenyan Shilling (~USD $3), 1500 Malawian Kwacha (~ USD $2), or

For peer review only

50 Zambian Kwacha (~USD $5) for their time, per local ethical guidelines. Participation in this study did not influence access to or participation in any of the DREAMS interventions.

For peer review only

REFERENCES

1. UNAIDS. Women and HIV: A Spotlight on Adolescent Girls and Young Women: UNAIDS, 2019.
2. UNAIDS. People Living With HIV: HIV Prevalence 2018 [Available from: https://aidsinfo.unaids.org/ accessed June 12 2020.
3. Ministry of Health Zambia. Zambia Population-based HIV Impact Assessment (ZAMPHIA) 2016: Final Report: Ministry of Health, 2019.
4. Ministry of Health Malawi. Malawi Population-Based HIV Impact Assessment (MPHIA) 2015-2016: Final Report: Ministry of Health, 2018.
5. Ministry of Health Kenya: National AIDS Control Council. Kenya HIV Estimates: Report 2018, 2018.
6. UNAIDS. HIV prevention among adolescent girls and young women. Geneva, Switzerland, 2016.
7. Santelli JS, Edelstein ZR, Mathur S, et al. Behavioral, biological, and demographic risk and protective factors for new HIV infections among youth, Rakai, Uganda. *J Acquir Immune Defic Syndr* 2013;63(3):393.
8. Mabaso M, Sokhela Z, Mohlabane N, et al. Determinants of HIV infection among adolescent girls and young women aged 15–24 years in South Africa: a 2012 population-based national household survey. *BMC Public Health* 2018;18(1):183.
9. Pettifor AE, Rees HV, Kleinschmidt I, et al. Young people's sexual health in South Africa: HIV prevalence and sexual behaviors from a nationally representative household survey. *Aids* 2005;19(14):1525-34.
10. Jewkes RK, Dunkle K, Nduna M, et al. Intimate partner violence, relationship power inequity, and incidence of HIV infection in young women in South Africa: a cohort study. *Lancet* 2010;376(9734):41-48.
11. Mathur S, Okal J, Musheke M, et al. High rates of sexual violence by both intimate and non-intimate partners experienced by adolescent girls and young women in Kenya and Zambia: Findings around violence and other negative health outcomes. *PLoS One* 2018;13(9)
12. Li Y, Marshall CM, Rees HC, et al. Intimate partner violence and HIV infection among women: a systematic review and meta-analysis. *Journal of the international AIDS society* 2014;17(1):18845.
13. Decker MR, Peitzmeier S, Olumide A, et al. Prevalence and health impact of intimate partner violence and non-partner sexual violence among female adolescents aged 15–19 years in vulnerable urban environments: a multi-country study. *Journal of Adolescent Health* 2014;55(6):S58-S67.
14. Balkus JE, Brown E, Palanee T, et al. An empiric HIV risk scoring tool to predict HIV-1 acquisition in African women. *J Acquir Immune Defic Syndr* 2016;72(3):333.
15. Mathur S, Wei Y, Zhong X, et al. Partner characteristics associated with HIV acquisition among youth in Rakai, Uganda. *J Acquir Immune Defic Syndr* 2015;69(1):75.
16. Harrison A, Cleland J, Frohlich J. Young people's sexual partnerships in KwaZulu-Natal, South Africa: patterns, contextual influences, and HIV risk. *Stud Fam Plann* 2008;39(4):295-308.
17. Wamoyi J, Buller AM, Nyato D, et al. "Eat and you will be eaten": a qualitative study exploring costs and benefits of age-disparate sexual relationships in Tanzania and Uganda: implications for girls' sexual and reproductive health interventions. *Reprod Health* 2018;15(1):207.
18. Chapman J, do Nascimento N, Mandal M. Role of male sex partners in HIV risk of adolescent girls and young women in Mozambique. *Global Health: Science and Practice* 2019;7(3):435-46.
19. Jewkes R, Dunkle K, Nduna M, et al. Transactional sex and HIV incidence in a cohort of young women in the stepping stones trial. *Journal of AIDS and Clinical research* 2012;3(5)

For peer review only

20. Kilburn K, Ranganathan M, Stoner MC, et al. Transactional sex and incident HIV infection in a cohort of young women from rural South Africa. *AIDS* 2018;32(12):1669.
21. Price JT, Rosenberg NE, Vansia D, et al. Predictors of HIV, HIV risk perception, and HIV worry among adolescent girls and young women in Lilongwe, Malawi. *J Acquir Immune Defic Syndr* 2018;77(1):53.
22. Birdthistle IJ, Floyd S, Machingura A, et al. From affected to infected? Orphanhood and HIV risk among female adolescents in urban Zimbabwe. *AIDS* 2008;22(6):759-66.
23. Karim QA, Kharsany AB, Leask K, et al. Prevalence of HIV, HSV-2 and pregnancy among high school students in rural KwaZulu-Natal, South Africa: a bio-behavioural cross-sectional survey. *Sex Transm Infect* 2014;90(8):620-26.
24. Nyirenda M, McGrath N, Newell M-L. Gender differentials in the impact of parental death: adolescent's sexual behaviour and risk of HIV infection in rural South Africa. *Vulnerable Child Youth Stud* 2010;5(3):284-96.
25. Operario D, Pettifor A, Cluver L, et al. Prevalence of parental death among young people in South Africa and risk for HIV infection. *J Acquir Immune Defic Syndr* 2007;44(1):93-98.
26. Nyato D, Materu J, Kuringe E, et al. Prevalence and correlates of partner violence among adolescent girls and young women: Evidence from baseline data of a cluster randomised trial in Tanzania. *PLoS One* 2019;14(10)
27. Hargreaves JR, Bonell CP, Boler T, et al. Systematic review exploring time trends in the association between educational attainment and risk of HIV infection in sub-Saharan Africa. *AIDS* 2008;22(3):403-14.
28. Toska E, Gittings L, Hodes R, et al. Resourcing resilience: social protection for HIV prevention amongst children and adolescents in Eastern and Southern Africa. *Afr J AIDS Res* 2016;15(2):123-40.
29. Saul J, Bachman G, Allen S, et al. The DREAMS core package of interventions: a comprehensive approach to preventing HIV among adolescent girls and young women. *PLoS One* 2018;13(12):e0208167.
30. Gourlay A, Birdthistle I, Mthiyane NT, et al. Awareness and uptake of layered HIV prevention programming for young women: analysis of population-based surveys in three DREAMS settings in Kenya and South Africa. *BMC Public Health* 2019;19(1):1417.
31. Office of the U.S. Global AIDS Coordinator and Health Diplomacy. Preventing HIV in adolescent girls and young women: guidance for PEPFAR country teams on the DREAMS partnership: President's Emergency Plan for AIDS Relief, 2016.
32. Office of the U.S. Global AIDS Coordinator and Health Diplomacy. DREAMS core package of interventions summary: President's Emergency Plan for AIDS Relief, 2017.
33. The Girl Roster: A Practical Tool for Strengthening Girl-Centered Programming. New York, NY: The Population Council, 2019.
34. The United States Department of State. Kenya: DREAMS Overview (FY 2016-2021), 2020.
35. The United States Department of State. Malawi: DREAMS Overview (FY 2016-2021), 2020.
36. The United States Department of State. Zambia: DREAMS Overview (FY 2016-2021), 2020.
37. PATH. The Power of Dreams: HIV Prevention for AGYW in Western Kenya, 2019.
38. USAID. USAID/Zambia Health Office: Zambia Community HIV Prevention Project (Z-CHPP) 2020 [Available from: https://www.usaid.gov/zambia/fact-sheets/usaidzambia-health-office-zambia-community-hiv-prevention-project-z.
39. One Community. What We Do: DREAMS 2016 [Available from: http://www.onecommunitymw.org/index.php/what-we-do/dreams.
40. Pact. DREAMS and OVC, 2020.

For peer review only

41. Dunkle KL, Jewkes RK, Brown HC, et al. Transactional sex among women in Soweto, South Africa: prevalence, risk factors and association with HIV infection. *Soc Sci Med* 2004;59(8):1581-92.

42. Jewkes R, Nduna M, Levin J, et al. A cluster randomized-controlled trial to determine the effectiveness of Stepping Stones in preventing HIV infections and promoting safer sexual behaviour amongst youth in the rural Eastern Cape, South Africa: trial design, methods and baseline findings. *Trop Med Int Health* 2006;11(1):3-16.

43. Mathur S, Pilgrim N, Patel SK, et al. HIV vulnerability among adolescent girls and young women: a multi-country latent class analysis approach. *Int J Public Health* 2020:1-13.

44. Hadijja Nakawooya, Victor Ssempijja, Dorean Nabukalu, et al. PEPFAR DREAMS Intervention Among Adolescent Girls and Young Women in Rakai, Uganda 2020 [Available from: https://www.croiconference.org/abstract/pepfar-dreams-intervention-among-adolescent-girls-and-young-women-in-rakai-uganda/.

45. Kenya National Bureau of Statistics, Ministry of Health/Kenya, National AIDS Control Council/Kenya, et al. Kenya Demographic and Health Survey 2014. Rockville, MD, USA, 2015.

46. Kalichman SC, Pellowski J, Turner C. Prevalence of sexually transmitted co-infections in people living with HIV/AIDS: systematic review with implications for using HIV treatments for prevention. *Sex Transm Infect* 2011;87(3):183-90.

47. Mathur S, Mullinax M, Santelli JS. Prevention of sexually transmitted infections among adolescents. In: Cherry A., Baltag V., M. D, eds. International handbook on adolescent health and development: Springer 2017:183-205.

48. Denno DM, Chandra-Mouli V, Osman M. Reaching youth with out-of-facility HIV and reproductive health services: a systematic review. *J Adolesc Health* 2012;51(2):106-21.

49. Jewkes R, Gibbs A, Jama-Shai N, et al. Stepping Stones and Creating Futures intervention: shortened interrupted time series evaluation of a behavioural and structural health promotion and violence prevention intervention for young people in informal settlements in Durban, South Africa. *BMC Public Health* 2014;14(1):1325.

50. Stockman JK, Lucea MB, Campbell JC. Forced sexual initiation, sexual intimate partner violence and HIV risk in women: a global review of the literature. *AIDS Behav* 2013;17(3):832-47.

51. National Statistical Office/Malawi, ICF. Malawi Demographic and Health Survey 2015-16. Zomba, Malawi: National Statistical Office and ICF, 2017.

52. García-Moreno C, Pallitto C, Devries K, et al. Global and regional estimates of violence against women: prevalence and health effects of intimate partner violence and non-partner sexual violence: World Health Organization, 2013.

53. Thurman T, Kidman R, Carton T, et al. Psychological and behavioral interventions to reduce HIV risk: evidence from a randomized control trial among orphaned and vulnerable adolescents in South Africa. *AIDS Care* 2016;28(sup1):8-15.

54. Hallman K, Mubayiwa R, Madya S, et al. Program versus Comparison Endline Survey of the Mabinti Tushike Hatamu! (Girls Lets Be Leaders!) Programme in Tanzania: Population Council, The CSR Group Africa Limited, Restless Development Tanzania, UNICEF Tanzania, Tanzania AIDS Commission, 2016.

55. Davis KR, Weller SC. The effectiveness of condoms in reducing heterosexual transmission of HIV. *Fam Plann Perspect* 1999:272-79.

56. Erulkar AS, Ettyang LI, Onoka C, et al. Behavior change evaluation of a culturally consistent reproductive health program for young Kenyans. *Int Fam Plan Perspect* 2004:58-67.

57. Erulkar A, Chong E. Evaluation of a savings & micro-credit program for vulnerable young women in Nairobi. Nairobi, Kenya, 2005.

58. Jewkes R, Nduna M, Levin J, et al. Impact of stepping stones on incidence of HIV and HSV-2 and sexual behaviour in rural South Africa: cluster randomised controlled trial. *BMJ* 2008;337:a506.

For peer review only - http://bmjopen.bmj.com/site/about/guidelines.xhtml

59. George G, Maughan-Brown B, Beckett S, et al. Coital frequency and condom use in age-disparate partnerships involving women aged 15 to 24: evidence from a cross-sectional study in KwaZulu-Natal, South Africa. *BMJ Open* 2019;9(3):e024362.
60. Pulerwitz J, Mathur S, Woznica D. How empowered are girls/young women in their sexual relationships? Relationship power, HIV risk, and partner violence in Kenya. *PLoS One* 2018;13(7)
61. Kalichman SC, Simbayi LC, Cain D, et al. Sexual assault, sexual risks and gender attitudes in a community sample of South African men. *AIDS Care* 2007;19(1):20-27.
62. Kosugi H, Shibanuma A, Kiriya J, et al. Consistent condom use among highly effective contraceptive users in an HIV-endemic area in rural Kenya. *PLoS One* 2019;14(5):e0216208.
63. Brahmbhatt H, Santelli J, Kaagayi J, et al. Pregnancy Incidence and Fertility Desires Among Couples by HIV Status in Rakai, Uganda. *JAIDS Journal of Acquired Immune Deficiency Syndromes* 2019;80(5):494-502.
64. Osei IF, Mayhew SH, Biekro L, et al. Fertility decisions and contraceptive use at different stages of relationships: windows of risk among men and women in accra. *International perspectives on sexual and reproductive health* 2014;40(3):135-43.
65. Rositch A, Cherutich P, Brentlinger P, et al. HIV infection and sexual partnerships and behaviour among adolescent girls in Nairobi, Kenya. *Int J STD AIDS* 2012;23(7):468-74.
66. Ranganathan M, Heise L, Pettifor A, et al. Transactional sex among young women in rural South Africa: prevalence, mediators and association with HIV infection. *J Int AIDS Soc* 2016;19(1):20749.
67. Gichane MW, Moracco KE, Pettifor AE, et al. Socioeconomic Predictors of Transactional Sex in a Cohort of Adolescent Girls and Young Women in Malawi: A Longitudinal Analysis. *AIDS Behav* 2020
68. Stoebenau K, Wamoyi J, Fielding-Miller R, et al. Transactional sex and HIV risk, 2017.
69. Chimbindi N, Birdthistle I, Floyd S, et al. Directed and target focused multi-sectoral adolescent HIV prevention: insights from implementation of the ‘DREAMS Partnership’in rural South Africa. *J Int AIDS Soc* 2020;23:e25575.
70. Cluver LD, Orkin MF, Yakubovich AR, et al. Combination social protection for reducing HIV-risk behavior amongst adolescents in South Africa. *J Acquir Immune Defic Syndr* 2016;72(1):96.
71. Dunbar MS, Dufour M-SK, Lambdin B, et al. The SHAZ! project: results from a pilot randomized trial of a structural intervention to prevent HIV among adolescent women in Zimbabwe. *PLoS One* 2014;9(11):e113621.
72. Cluver L, Toska E, Orkin F, et al. Achieving equity in HIV-treatment outcomes: can social protection improve adolescent ART-adherence in South Africa? *AIDS Care* 2016;28(sup2):73-82.
73. Bandiera O, Buehren N, Burgess R, et al. Women’s empowerment in action: evidence from a randomized control trial in Africa: World Bank, 2018.
74. STRIVE Research Consortium. Why STRIVE?: London School of Hygiene & Tropical Medicine, 2016.
75. STRIVE Research Consortium. About STRIVE 2020 [Available from: http://strive.lshtm.ac.uk/about.
76. Delany-Moretlwe S, Hargreaves J, Stangly A, et al. Biomedical and structural prevention: STRIVE in the era of ‘cascades’, 2018.
77. Temin M, Heck CJ. Close to Home: Evidence on the Impact of Community-Based Girl Groups. *Glob Health Sci Pract* 2020;8(2):300-24.

For peer review only

For peer review only - http://bmjopen.bmj.com/site/about/guidelines.xhtml

Supplemental Table 1. Loss-to-follow-up analysis of adolescent girls and young women interviewed at Round 1 in Kenya, Malawi, and Zambia

Characteristics/Outcomes	Kenya				Malawi				Zambia			
	15-19		20-24		15-19		20-24		15-19		20-24	
	Followed n=389	Lost n=85	Followed n=347	Lost n=93	Followed n=371	Lost n=159	Followed n=883	Lost n=250	Followed n=487	Lost n=98	Followed n=398	Lost n=81
**Age**												
Mean (SD)	16.6 (1.3)	16.7 (1.3)	21.6 (1.4)	21.4 (1.4)	18.0 (1.2)	17.9 (1.2)	21.9 (1.4)	22.0 (1.5)	16.8 (1.4)	16.7 (1.4)	21.6 (1.3)	21.5 (1.3)
**Marital Status**												
Ever Married	4.4	4.7	44.4	35.5	62.3	55.4	88.9	86.8	2.5	2.0	8.5	6.2
Never married	95.6	95.3	55.6	64.5	37.7	44.6	11.1	13.2	97.5	98.0	91.5	93.8
**Current Schooling Status**	*											
In school	84.3	75.3	32.9	28.0	-	-	-	-	77.6	82.7	61.6	60.5
Out of school	15.7	24.7	67.1	72.0	-	-	-	-	22.4	17.3	38.4	39.5
**Orphanhood**	**											
Both parents alive	62.5	45.9	42.4	37.6	70.9	74.8	61.4	61.4	64.9	61.2	55.5	51.9
Lost at least one parent	37.5	54.1	57.6	62.4	29.1	25.2	38.6	38.8	35.1	38.8	44.5	48.1
**Site**					**		*		**			
Location 1	49.6	56.5	47.8	55.9	33.7	19.5	31.7	23.2	37.0	55.1	64.6	71.6
Location 2	50.4	43.5	52.2	44.1	66.3	80.5	68.3	76.8	63.0	44.9	35.4	28.4
**HIV Service Use**												
HIV test in last 12 months	**											
No	18.5	5.9	3.7	0.0	14.6	14.1	17.5	19.4	52.6	53.1	32.4	33.3
Yes	81.5	94.1	96.3	100.0	85.4	85.9	82.5	80.6	47.4	46.9	67.6	66.7
**Sexual Behavior**												

For peer review only

Experience of Sexually transmitted infection symptoms									*			
No	85.6	82.3	76.9	78.5	74.4	69.2	68.2	65.6	87.3	78.6	84.2	82.7
Yes	14.4	17.7	23.1	21.5	25.6	30.8	31.8	34.4	12.7	21.4	15.8	17.3
Number of sex partners									***			
	n=126	n=35	n=317	n=87	n=329	n=130	n=876	n=245	n=106	n=32	n=254	n=46
0	11.1	8.6	5.1	4.6	20.4	21.5	23.5	24.5	7.6	6.3	15.0	8.7
1	73.0	80.0	79.8	80.5	70.2	67.7	70.3	72.6	85.8	62.5	72.0	73.9
2+	15.9	11.4	15.1	14.9	9.4	10.8	6.2	2.9	6.6	31.3	13.0	17.4
Consistent condom use												
	n=59	n=19	n=218	n=59	n=238	n=92	n=684	n=184	n=40	n=8	n=97	n=16
No	47.5	47.4	69.3	66.1	88.2	91.3	95.0	94.0	57.5	50.0	57.7	56.3
Yes	52.5	52.6	30.7	33.9	11.8	8.7	5.0	6.0	42.5	50.0	42.3	43.8
Condom use at last sex												
	n=59	n=19	n=218	n=59	n=238	n=92	n=684	n=184	n=40	n=8	n=97	n=16
No	25.4	26.3	53.7	44.1	79.8	76.1	86.0	84.2	40.0	25.0	47.4	43.8
Yes	74.6	73.7	46.3	55.9	20.2	23.9	14.0	15.8	60.0	75.0	52.6	56.3
Transactional sex									*			
	n=126	n=35	n=317	n=87	n=329	n=130	n=876	n=245	n=107	n=32	n=254	n=46
No	90.5	85.7	94.3	94.3	97.6	96.2	96.1	96.7	99.1	90.6	97.6	97.8
Yes	9.5	14.3	5.7	5.7	2.4	3.9	3.9	3.3	0.9	9.4	2.4	2.2
**Violence perpetrated by an intimate partner**	n=197	n=48	n=257	n=72	n=338	n=143	n=878	n=244	n=296	n=65	n=347	n=68

For peer review only

Experience of sexual violence from partners							*		*			
No	80.2	87.5	79.8	81.9	83.1	81.1	83.6	77.1	82.4	69.2	81.6	72.1
Yes	19.8	12.5	20.2	18.1	16.9	18.9	16.4	22.9	17.6	30.8	18.4	27.9
Experience of physical violence from partners												
No	84.8	83.3	68.9	70.8	86.1	86.0	82.8	85.3	76.7	75.4	84.4	76.5
Yes	15.2	16.7	31.1	29.2	13.9	14.0	17.2	14.8	23.3	24.6	15.6	23.5
**Violence perpetrated by a non-partner**												
Experience of sexual violence from non-partners			*									
No	78.9	78.8	69.5	81.7	90.0	88.1	91.5	91.6	87.5	84.7	76.6	76.5
Yes	21.1	21.2	30.5	18.3	10.0	12.0	8.5	8.4	12.5	15.3	23.4	23.5

*p≤0.05; **p≤0.01; ***p≤0.001

For peer review only

STROBE Statement—Checklist of items that should be included in reports of ***cohort studies***

	Item No	Recommendation	Page No
**Title and abstract**	1	(*a*) Indicate the study's design with a commonly used term in the title or the abstract	Title
		(*b*) Provide in the abstract an informative and balanced summary of what was done and what was found	1-3
**Introduction**			
Background/rationale	2	Explain the scientific background and rationale for the investigation being reported	5-6
Objectives	3	State specific objectives, including any prespecified hypotheses	6
**Methods**			
Study design	4	Present key elements of study design early in the paper	7
Setting	5	Describe the setting, locations, and relevant dates, including periods of recruitment, exposure, follow-up, and data collection	8-9
Participants	6	(*a*) Give the eligibility criteria, and the sources and methods of selection of participants. Describe methods of follow-up<br>(*b*) For matched studies, give matching criteria and number of exposed and unexposed	8-9
Variables	7	Clearly define all outcomes, exposures, predictors, potential confounders, and effect modifiers. Give diagnostic criteria, if applicable	9-11
Data sources/ measurement	8*	For each variable of interest, give sources of data and details of methods of assessment (measurement). Describe comparability of assessment methods if there is more than one group	9-11
Bias	9	Describe any efforts to address potential sources of bias	
Study size	10	Explain how the study size was arrived at	8-9
Quantitative variables	11	Explain how quantitative variables were handled in the analyses. If applicable, describe which groupings were chosen and why	9-11
Statistical methods	12	(*a*) Describe all statistical methods, including those used to control for confounding<br>(*b*) Describe any methods used to examine subgroups and interactions<br>(*c*) Explain how missing data were addressed<br>(*d*) If applicable, explain how loss to follow-up was addressed<br>(*e*) Describe any sensitivity analyses	12
**Results**			
Participants	13*	(a) Report numbers of individuals at each stage of study—eg numbers potentially eligible, examined for eligibility, confirmed eligible, included in the study, completing follow-up, and analysed<br>(b) Give reasons for non-participation at each stage<br>(c) Consider use of a flow diagram	12-14
Descriptive data	14*	(a) Give characteristics of study participants (eg demographic, clinical, social) and information on exposures and potential confounders<br>(b) Indicate number of participants with missing data for each variable of interest<br>(c) Summarise follow-up time (eg, average and total amount)	12-14
Outcome data	15*	Report numbers of outcome events or summary measures over time	15-18

For peer review only

Main results	16	(*a*) Give unadjusted estimates and, if applicable, confounder-adjusted estimates and their precision (eg, 95% confidence interval). Make clear which confounders were adjusted for and why they were included<br>(*b*) Report category boundaries when continuous variables were categorized<br>(*c*) If relevant, consider translating estimates of relative risk into absolute risk for a meaningful time period	15-18
Other analyses	17	Report other analyses done—eg analyses of subgroups and interactions, and sensitivity analyses	
**Discussion**			
Key results	18	Summarise key results with reference to study objectives	19
Limitations	19	Discuss limitations of the study, taking into account sources of potential bias or imprecision. Discuss both direction and magnitude of any potential bias	23
Interpretation	20	Give a cautious overall interpretation of results considering objectives, limitations, multiplicity of analyses, results from similar studies, and other relevant evidence	19-23
Generalisability	21	Discuss the generalisability (external validity) of the study results	19-23
**Other information**			
Funding	22	Give the source of funding and the role of the funders for the present study and, if applicable, for the original study on which the present article is based	25

*Give information separately for exposed and unexposed groups.

Note: An Explanation and Elaboration article discusses each checklist item and gives methodological background and published examples of transparent reporting. The STROBE checklist is best used in conjunction with this article (freely available on the Web sites of PLoS Medicine at http://www.plosmedicine.org/, Annals of Internal Medicine at http://www.annals.org/, and Epidemiology at http://www.epidem.com/). Information on the STROBE Initiative is available at http://www.strobe-statement.org.

For peer review only

For peer review only - http://bmjopen.bmj.com/site/about/guidelines.xhtml
